# The utility of a type 2 diabetes polygenic score in addition to clinical variables for prediction of type 2 diabetes incidence in birth, youth and adult cohorts in an Indigenous study population

**DOI:** 10.1007/s00125-023-05870-2

**Published:** 2023-03-02

**Authors:** Lauren E. Wedekind, Anubha Mahajan, Wen-Chi Hsueh, Peng Chen, Muideen T. Olaiya, Sayuko Kobes, Madhumita Sinha, Leslie J. Baier, William C. Knowler, Mark I. McCarthy, Robert L. Hanson

**Affiliations:** 1 Phoenix Epidemiology and Clinical Research Branch, National Institute of Diabetes and Digestive and Kidney Diseases, National Institutes of Health, Phoenix, AZ USA; 2 Nuffield Department of Medicine, University of Oxford, Oxford, UK; 3 Wellcome Centre for Human Genetics, University of Oxford, Oxford, UK; 4 Present Address: Genentech, San Francisco, CA USA; 5 College of Basic Medical Sciences, Jilin University, Changchun, China; 6 School of Clinical Sciences, Monash University, Clayton, VIC Australia; 7 Oxford Centre for Diabetes, Endocrinology and Metabolism, University of Oxford, Headington, UK

**Keywords:** Clinical prediction, Decision curve analysis, Incidence analysis, Polygenic score, Type 2 diabetes

## Abstract

**Aims/hypothesis:**

There is limited information on how polygenic scores (PSs), based on variants from genome-wide association studies (GWASs) of type 2 diabetes, add to clinical variables in predicting type 2 diabetes incidence, particularly in non-European-ancestry populations.

**Methods:**

For participants in a longitudinal study in an Indigenous population from the Southwestern USA with high type 2 diabetes prevalence, we analysed ten constructions of PS using publicly available GWAS summary statistics. Type 2 diabetes incidence was examined in three cohorts of individuals without diabetes at baseline. The adult cohort, 2333 participants followed from age ≥20 years, had 640 type 2 diabetes cases. The youth cohort included 2229 participants followed from age 5–19 years (228 cases). The birth cohort included 2894 participants followed from birth (438 cases). We assessed contributions of PSs and clinical variables in predicting type 2 diabetes incidence.

**Results:**

Of the ten PS constructions, a PS using 293 genome-wide significant variants from a large type 2 diabetes GWAS meta-analysis in European-ancestry populations performed best. In the adult cohort, the AUC of the receiver operating characteristic curve for clinical variables for prediction of incident type 2 diabetes was 0.728; with the PS, 0.735. The PS’s HR was 1.27 per SD (*p*=1.6 × 10^−8^; 95% CI 1.17, 1.38). In youth, corresponding AUCs were 0.805 and 0.812, with HR 1.49 (*p*=4.3 × 10^−8^; 95% CI 1.29, 1.72). In the birth cohort, AUCs were 0.614 and 0.685, with HR 1.48 (*p*=2.8 × 10^−16^; 95% CI 1.35, 1.63). To further assess the potential impact of including PS for assessing individual risk, net reclassification improvement (NRI) was calculated: NRI for the PS was 0.270, 0.268 and 0.362 for adult, youth and birth cohorts, respectively. For comparison, NRI for HbA_1c_ was 0.267 and 0.173 for adult and youth cohorts, respectively. In decision curve analyses across all cohorts, the net benefit of including the PS in addition to clinical variables was most pronounced at moderately stringent threshold probability values for instituting a preventive intervention.

**Conclusions/interpretation:**

This study demonstrates that a European-derived PS contributes significantly to prediction of type 2 diabetes incidence in addition to information provided by clinical variables in this Indigenous study population. Discriminatory power of the PS was similar to that of other commonly measured clinical variables (e.g. HbA_1c_). Including type 2 diabetes PS in addition to clinical variables may be clinically beneficial for identifying individuals at higher risk for the disease, especially at younger ages.

**Graphical abstract:**

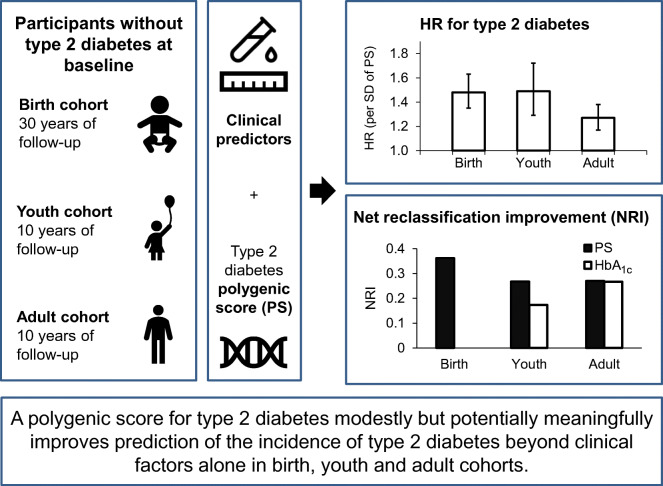

**Supplementary Information:**

The online version of this article (10.1007/s00125-023-05870-2) contains peer-reviewed but unedited supplementary material.

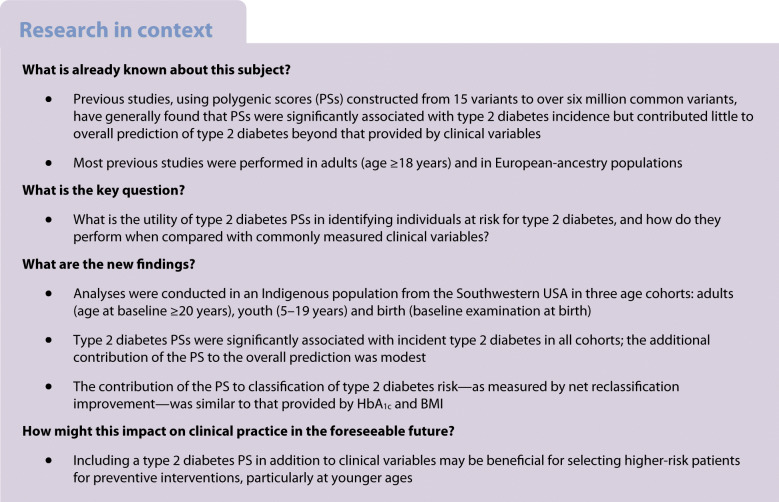



## Introduction

Type 2 diabetes-associated genetic variants, derived from genome-wide association studies (GWASs), have largely been reproducible across populations. There is limited information on how polygenic scores (PSs) based on these variants add to clinical variables for predicting type 2 diabetes incidence. Such prediction could help identify individuals at increased risk of type 2 diabetes for targeted prevention efforts.

Previous studies assessing contributions of a type 2 diabetes PS for prediction of type 2 diabetes incidence have mostly been conducted in European-ancestry populations [1, 2, 3, 4, 5, 6]. These studies, using PSs constructed from 15 variants to over six million common variants, have generally found that PSs were significantly associated with type 2 diabetes incidence but contributed little beyond clinical variables to overall prediction of type 2 diabetes [1, 2, 3, 4, 5, 6].

Previous studies were largely conducted in adults, but the utility of PSs for prediction of subsequent type 2 diabetes may be greater earlier in life (in youth or even at birth). The present study employed a PS for prediction of type 2 diabetes incidence in an Indigenous population from the Southwestern USA with a high prevalence of type 2 diabetes and obesity, and in which long-term follow-up data are available. In this population, the age-adjusted prevalence of diabetes is approximately six times higher than in non-Hispanic white people in the USA [7]. We aimed to analyse how genetic and clinical variables could inform strategies for screening and prevention in three cohorts of individuals in different age groups (birth, youth and adulthood) at baseline.

## Methods

### Study design and participants

A longitudinal study of diabetes (1965–2007) was conducted in an Indigenous study population from the Southwestern USA; methods for this study have been described previously [8]. Before participation, volunteers were fully informed of the nature and purpose of the study and adult participants provided written informed consent, including consent for genetic studies; minor participants provided written assent. Protocols were approved by the institutional review board of the National Institute of Diabetes and Digestive and Kidney Diseases, and research was conducted in accordance with the principles of the Declaration of Helsinki.

Briefly, individuals at least 5 years old were invited for health examinations every 2 years. At each exam, a 75 g oral glucose tolerance test was administered with measurement of HbA_1c_ and fasting and 2 h plasma glucose (FPG, 2hPG). Diabetes was diagnosed using 1997 American Diabetes Association criteria (FPG ≥7.0 mmol/l, 2hPG ≥11.1 mmol/l or clinical diagnosis) [9]. Height and weight were measured to calculate BMI and birthweight was collected from clinical information and Arizona state birth certificates. Participants had not been directly asked to report parental diabetes; however, since many participants’ parents had also participated in the study, we were able to approximate the information that would be available in clinical encounters by using information from direct examination in the parents. We defined parental diabetes using three categories (yes, no or unknown) per parent. Characteristics of participants are summarised in electronic supplementary material (ESM) Tables 1 and 2.

### Genotypic data

Of the study participants, 7701 had genotypes available from previous GWASs, generated using a custom Axiom array designed to capture common variation in members of this community (minor allele frequency (MAF) ≥0.05, or ≥0.01 for coding variants), using methods described previously (Affymetrix, Santa Clara, CA, USA) [10]. Missing and ungenotyped variants were imputed with whole genome sequence data for 266 community members as a reference panel using Impute 2, resulting in 6.6 million variants with MAF >0.01 and imputation quality score >0.5 (median 0.95) [11]. Previous work in this population suggests that a population-specific reference panel is optimal for imputing common variants, with little value from including samples from outside populations [12]. Variants were excluded from analyses if they had an imputation quality score <0.5 or MAF <0.01 (ESM Method 1).

### Study cohorts

Of the 7701 individuals with genotypes available, we constructed three cohorts based on age at baseline examination for those who had data for at least two exams with availability of clinical variables. There were 2333 participants followed from first examination in adulthood (age ≥20 years); 640 cases of type 2 diabetes occurred over 16,686 person-years of follow-up. There were 2229 participants followed from first examination in youth (age 5–19 years); 228 cases of type 2 diabetes occurred over 17,803 person-years of follow-up. There were 2894 participants with birthweight data available who were considered to be followed from birth; 438 cases of type 2 diabetes occurred over 61,591 person-years of follow-up. Individuals were included in multiple cohorts if suitable data were available.

### Construction of type 2 diabetes PSs

We compared associations of ten different constructions of type 2 diabetes PS, derived from GWASs conducted for populations from various world regions. We used ‘pruning and thresholding’ methods to select variants for the PS, selecting independent genome-wide significant variants from the GWASs for other populations (ESM Tables 3–6). These PSs included the following, each named for the meta-analysis from which it was derived: Diabetes Genetics Replication And Meta-analysis consortium (DIAGRAM) 2018 (constituting 293 variants derived from European populations) [13], Asian Genetic Epidemiology Network consortium (AGEN) 2020 (125 variants derived from East Asian populations) [14] and Diabetes Meta-Analysis of Trans-Ethnic association studies consortium (DIAMANTE) 2022. The seven DIAMANTE PSs are constructions of multi-ancestry PSs (287 variants) with weights taken from meta-analyses of populations representing the following ancestry groups: multi-ancestry, African, East Asian, European, Hispanic/Latino and South Asian [15], in addition to a ‘population-specific weight’ PS from these same DIAMANTE 2022 variants with weights derived from the present population, using tenfold cross-validation to address overfitting. Finally, we also derived a ‘population-specific variant’ PS by selecting 287 type 2 diabetes-associated variants from the 515,692 variants typed in the type 2 diabetes GWAS in the study population, using twofold cross-validation (ESM Method 2). While PSs can be constructed using a larger number of variants, by using less stringent significance thresholds or accounting for linkage disequilibrium, applicability across populations with different linkage disequilibrium patterns is uncertain. We, thus, employed the widely used method of selecting significant variants.

We constructed each PS using imputed genotypes available in the present study population. The products of the number of risk alleles for each individual with the effect size (logarithm of the OR) from the corresponding GWAS were summed across variants. PSs were standardised across the entire study population to have mean of 0 and SD of 1: HRs for PSs were expressed in terms of SD of that PS.

### Statistical analyses

Analyses were completed in SAS 9.4 (SAS Institute, Cary, NC, USA). For each cohort, individuals were followed from inception (first examination with clinical data available for youth and adult cohorts; birth for birth cohort) until they developed type 2 diabetes or until their last examination, whichever came first. We evaluated the relative contributions of various combinations of clinical variables and/or the PS in the following analyses: cumulative incidence, survival, AUC of the receiver operating characteristic curve, net reclassification improvement (NRI) and decision curve. Cumulative incidence, survival, decision curve and NRI analyses required calculation of the predicted occurrence of type 2 diabetes at a specified follow-up time for all individuals to ensure comparability: a follow-up of 10 years was used for the adult and youth cohorts, and 30 years for the birth cohort.

The variables available for the adult cohort included: age, sex, parental diabetes, BMI, HbA_1c_, FPG, 2hPG and the type 2 diabetes PS [16]. Those for the youth cohort included: age, sex, parental diabetes, modified BMI *z* score [17], HbA_1c_, FPG, 2hPG and PS. Those for the birth cohort included: sex, parental diabetes, birthweight and PS. This specific set of clinical variables was chosen because the US Preventive Services Task Force focuses on measures of obesity, family history and hyperglycaemia in recommendations for screening and prevention of type 2 diabetes [18]. Most previous studies have assessed prediction with control for a similar set of clinical predictors as used here, but many studies have also included measurement of lipids [1, 2, 3, 4, 5, 6]. Measurements of serum HDL and triglycerides/triacylglycerols were available in a subset of the present cohort (measured since 1993) and adjustment for these variables yielded similar results to those observed in primary analyses (ESM Table 7).

Since HbA_1c_ was only measured for examinations after 1989, we conducted additional analyses that did not require HbA_1c_ to allow for longer follow-up and greater sample size: these analyses returned similar findings to analyses that included HbA_1c_ (ESM Table 8). Given the U-shaped relationship between birthweight and type 2 diabetes in this population [19], we analysed birthweight using two binary variables, one denoting birthweight <3000 g and another denoting birthweight >4000 g. We also conducted analyses that included a continuous birthweight variable and its squared term to capture the quadratic relationship between birthweight and type 2 diabetes. While these analyses gave similar results, the dichotomised birthweight variables yielded a better fit according to Akaike’s information criterion.

We also conducted analyses including stated admixture as a covariate; its inclusion returned virtually the same results as without. To further control for population stratification, we conducted additional analyses after adjustment of the PS for the first ten genetic principal components derived from the GWAS, with separate estimation of principal components in each of the three target cohorts; results were similar to those of the primary analyses (ESM Table 9). Previous studies in this population demonstrated that genetic variants at *KCNQ1* rs2237895 (risk allele frequency=0.49, OR 1.31; exhibits parent-of-origin effects) [20] and *ABCC8* rs1272388614 (risk allele frequency=0.017, OR 2.02) [21] are significantly and strongly associated with type 2 diabetes. We conducted further analyses to assess the contributions of these genotypes in addition to the type 2 diabetes PS for prediction of type 2 diabetes incidence.

### Cumulative incidence and survival analyses

We used Cox proportional hazards regression to evaluate associations of clinical variables and PS with type 2 diabetes incidence. Cumulative incidence of type 2 diabetes was calculated as the proportion of individuals that developed type 2 diabetes over the specified follow-up time, using Breslow’s method (PROC PHREG in SAS). To assess separate contributions of PS and clinical risk, we calculated predicted cumulative incidence according to different levels of PS and of clinical risk, as determined by linear predictors from the clinical variables in the proportional hazards model.

### AUC analyses

We compared the Harrell’s C statistic [22] of models that included clinical variables alone with the C statistic of those that included clinical variables and the PS. The C statistic expresses the probability within a pair of individuals, one who developed type 2 diabetes and one who did not, that the individual who developed type 2 diabetes had a higher predicted probability of doing so [23]. In the context of survival analysis (e.g. in the proportional hazards models used here), the C statistic is equivalent to the AUC of the receiver operating characteristic curve [23], and we refer to it as ‘AUC’ throughout the manuscript.

### NRI analyses

Continuous-variable NRI quantifies the amount of correct reclassification introduced by using a model with an additional variable [24]. We analysed NRI by calculating the net proportion of events reclassified correctly (assigned a higher probability value) plus the net proportion of nonevents reclassified correctly (assigned a lower probability value) [25]. Confidence intervals for the NRI were calculated by a bootstrap method.

### Decision curve analyses

We employed decision-analytic methods to assess consequences of clinical decisions and expected outcomes of alternative clinical management (i.e. including various combinations of clinical variables with and without the PS in prediction models). These analyses assume that the threshold probability (*p*_*t*_) of developing type 2 diabetes at which one would opt for an intervention is informative of how one weighs the relative benefits and harms of true-positive and false-positive predictions, and the net benefit of using a predictive model to select individuals above a given *p*_*t*_ is calculated accordingly [26]. We used extensions to decision curve methods for survival analysis to plot net benefit across a range of *p*_*t*_ values to evaluate for which *p*_*t*_ ranges and what corresponding proportion of the population the PS had marginal net benefit [27].

### Comparisons of associations of PSs among cohorts

To compare the effects of the PSs (e.g. HRs) among the different age cohorts, a bootstrap analysis was conducted as previously described [28]. In brief, the 4770 individuals who were included in at least one cohort were resampled 2000 times, and the analyses were repeated for each iteration. The resulting differences in the logarithm of the HR between each pair of cohorts and their standard errors were calculated and used to test statistical significance of the differences. Since the availability and predictive power of different clinical covariates may affect the HR estimates, these analyses were conducted without any covariates.

## Results

### Ten constructions of type 2 diabetes PSs

All ten PSs for type 2 diabetes, constructed using the overlap of published type 2 diabetes GWAS summary statistics and genotypes available in this study population, had significant associations with type 2 diabetes incidence in the study population. HRs for the PSs in models adjusted for clinical variables (age, sex, BMI, FPG, HbA_1c_ and parental diabetes for the adult cohort; age, sex, modified BMI *z* score, FPG, HbA_1c_ and parental diabetes for the youth cohort; and sex, birthweight and parental diabetes for the birth cohort) ranged from 1.13 to 1.27 per SD for the adult cohort, from 1.19 to 1.49 for the youth cohort and from 1.27 to 1.48 for the birth cohort (ESM Table 10). The PS that consistently had the strongest associations with type 2 diabetes incidence (largest HRs) was constructed using the DIAGRAM 2018 GWAS. Thus, for the rest of this text, we present results for the DIAGRAM 2018 PS. Calibration plots for models for this PS for each of the three cohorts show that these models are well-calibrated (ESM Fig. 1). The DIAMANTE 2022 multi-ancestry PS and the population-specific variant PS also had strong associations with type 2 diabetes incidence, though not as strong (ESM Tables 10–12 and ESM Figs 2–7).

### Association of PS with incidence of type 2 diabetes

**Fig. 1:**
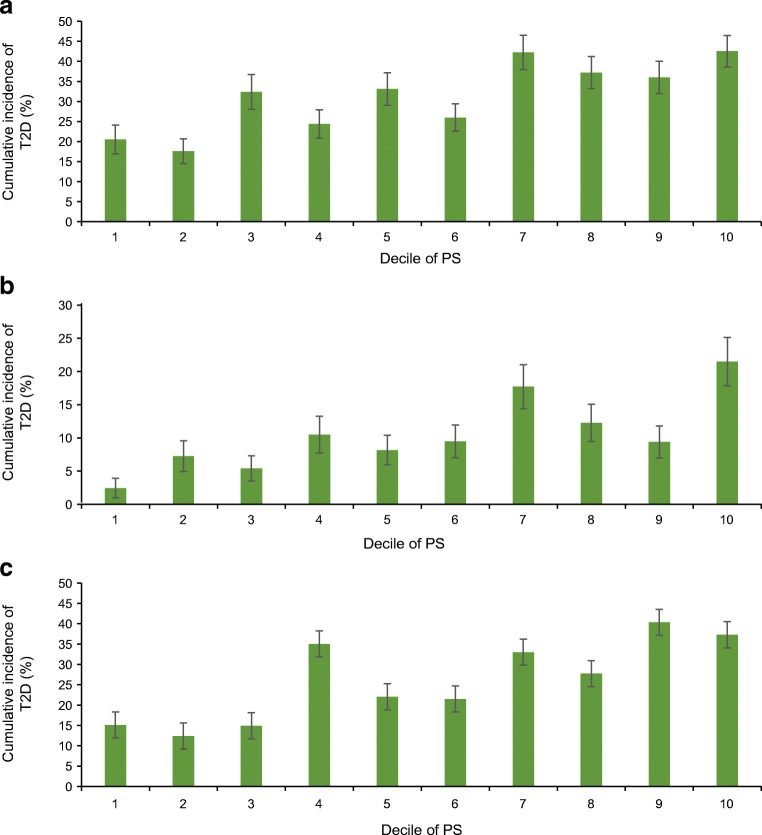
Cumulative incidence of type 2 diabetes by decile of the DIAGRAM 2018 PS. The PS was significantly associated with type 2 diabetes incidence in adult, youth and birth cohorts. (**a**) Cumulative incidence in the adult cohort at 10 year follow-up; at 10 years, 504 individuals had developed type 2 diabetes and 635 remained at risk. (**b**) Cumulative incidence in the youth cohort at 10 year follow-up; at 10 years, 152 individuals had developed type 2 diabetes and 745 remained at risk. (**c**) Cumulative incidence in the birth cohort at 30 year follow-up; at 30 years, 340 individuals had developed type 2 diabetes and 474 remained at risk. T2D, type 2 diabetes

The best-performing PS was significantly associated with type 2 diabetes incidence in adult, youth and birth cohorts (Fig. 1). In the adult cohort, 10 year cumulative incidence of type 2 diabetes in the lowest decile of PS was 20.5%; in the highest, 42.5% (unadjusted HR=1.31 per SD, *p*=6.9 × 10^−11^). In the youth cohort, 10 year cumulative incidence of type 2 diabetes in the lowest decile of PS was 2.4%; in the highest, 21.5% (HR=1.59 per SD, *p*=6.8 × 10^−12^). In the birth cohort, 30 year cumulative incidence of type 2 diabetes in the lowest decile of PS was 15.1%; in the highest, 37.3% (HR=1.47 per SD, *p*=1.7 × 10^−15^). The clinical predictors were also strongly associated with incidence of type 2 diabetes (ESM Fig. 8).

### Survival analyses with adjustment for clinical predictors

We conducted survival analyses to assess associations of individual clinical variables and the PS with type 2 diabetes incidence. In the adult cohort, in the model with clinical variables, the HR of the PS was 1.27 per SD (*p*=1.6 × 10^−8^; 95% CI 1.17, 1.38; Table 1). In the youth cohort, in the model with clinical variables, the HR of the PS was 1.49 (*p*=4.3 × 10^−8^; 95% CI 1.29, 1.72) (Table 2). In the birth cohort, in the model with clinical variables, the HR of the PS was 1.48 (*p*=2.8 × 10^−16^; 95% CI 1.35, 1.63) (Table 3). Adding 2hPG to adult and youth cohorts’ models did not substantially alter the HRs of the PS. In general, the HRs associated with clinical variables were only modestly affected with the addition of the PS.

**Table 1:** Results of survival, AUC and NRI analyses for the adult cohort

Variable	Clinical variables (AUC=0.728)			Clinical variables + PS (AUC=0.735)			Clinical variables + 2hPG (AUC=0.760)			Clinical variables + 2hPG + PS (AUC=0.765)		
	HR (95% CI)	*p* value	NRI	HR (95% CI)	*p* value	NRI	HR (95% CI)	*p* value	NRI	HR (95% CI)	*p* value	NRI
Age (decades)	1.01 (0.93, 1.10)	0.869	0.011	1.04 (0.95, 1.13)	0.393	0.067	1.00 (0.92, 1.09)	0.992	−0.045	1.03 (0.94, 1.12)	0.540	0.112
Sex (F/M)	1.35 (1.14, 1.59)	5.48 × 10^−4^	0.115	1.34 (1.13, 1.58)	7.70 × 10^−4^	0.116	1.13 (0.953, 1.34)	0.157	0.110	1.13 (0.954, 1.35)	0.154	0.106
Mother diabetic/NonDb	1.52 (1.21, 1.90)	2.75 × 10^−4^	0.161	1.46 (1.17, 1.83)	2.08 × 10^−3^	0.140	1.43 (1.14, 1.80)	2.60 × 10^−3^	0.178	1.38 (1.10, 1.73)	0.0131	0.126
Mother unknown/NonDb	1.45 (1.15, 1.82)			1.38 (1.09, 1.74)			1.39 (1.10, 1.74)			1.33 (1.06, 1.67)		
Father diabetic/NonDb	1.45 (1.06, 1.96)			1.38 (1.01, 1.87)			1.38 (1.02, 1.88)			1.33 (0.975, 1.81)		
Father unknown/NonDb	1.20 (0.921, 1.56)			1.17 (0.900, 1.52)			1.20 (0.93, 1.56)			1.17 (0.900, 1.52)		
BMI (kg/m^2^)	1.02 (1.01, 1.03)	1.22 × 10^−4^	0.158	1.03 (1.01, 1.03)	1.03 × 10^−5^	0.201	1.02 (1.01, 1.03)	2.11 × 10^−5^	0.242	1.03 (1.02, 1.04)	1.88 × 10^−6^	0.269
FPG (mmol/l)	1.99 (1.70, 2.33)	2.78 × 10^−17^	0.303	1.95 (1.66, 2.29)	3.13 × 10^−16^	0.295	1.44 (1.22, 1.71)	2.51 × 10^−4^	0.176	1.44 (1.21, 1.71)	2.93 × 10^−5^	0.191
HbA_1c_ (mmol/mol)	1.08 (106, 1.10)	1.18 × 10^−18^	0.272	1.08 (1.06, 1.10)	1.14 × 10^−17^	0.267	1.06 (1.05, 1.08)	9.64 × 10^−13^	0.215	1.06 (1.04, 1.08)	5.67 × 10^−12^	0.223
2hPG (mmol/l)	–	–	–	–	–	–	1.32 (1.25, 1.40)	3.74 × 10^−24^	0.420	1.31 (1.24, 1.38)	6.71 × 10^−23^	0.426
PS (SD)	–	–	–	1.27 (1.17, 1.38)	1.61 × 10^−8^	0.270	–	–	–	1.24 (1.15, 1.35)	2.90 × 10^−7^	0.252

‘Clinical variables’ refers to: age, sex, parental diabetes, BMI and FPG for the adult cohort

NRI is calculated for each predictor by comparing the model including the predictor with a model that does not include the predictor

Db, diabetic with regard to parental diabetes; F, female; M, male

**Table 2:** Results of survival, AUC and NRI analyses for the youth cohort

Variable	Clinical variables (AUC=0.805)			Clinical variables + PS (AUC=0.812)			Clinical variables + 2hPG (AUC=0.820)			Clinical variables + 2hPG + PS (AUC=0.825)		
	HR (95% CI)	*p* value	NRI	HR (95% CI)	*p* value	NRI	HR (95% CI)	*p* value	NRI	HR (95% CI)	*p* value	NRI
Age (decades)	2.11 (1.46, 3.04)	6.50 × 10^−5^	0.373	2.21 (1.53, 3.18)	2.02 × 10^−5^	0.372	2.15 (1.49, 3.11)	4.96 × 10^−5^	0.367	2.26 (1.57, 3.27)	1.37 × 10^−5^	0.382
Sex (F/M)	1.40 (1.06, 1.84)	0.0170	0.046	1.32 (1.01, 1.74)	0.0458	0.046	1.09 (0.816, 1.45)	0.562	0.020	1.04 (0.78, 1.39)	0.794	0.032
Mother diabetic/NonDb	2.46 (1.83, 3.29)	1.21 × 10^−11^	0.524	2.27 (1.70, 3.05)	1.28 × 10^−9^	0.514	2.26 (1.68, 3.04)	2.38 × 10^−9^	0.457	2.10 (1.56, 2.83)	1.29 × 10^−7^	0.472
Mother unknown/NonDb	1.84 (1.27, 2.68)			1.87 (1.29, 2.72)			1.82 (1.26, 2.64)			1.83 (1.26, 2.65)		
Father diabetic/NonDb	1.88 (1.27, 2.78)			1.72 (1.16, 2.55)			1.74 (1.18, 2.57)			1.61 (1.09, 2.39)		
Father unknown/NonDb	1.01 (0.740, 1.37)			0.972 (0.714, 1.32)			1.01 (0.743, 1.37)			0.977 (0.719, 1.33)		
Modified BMI *z* score	1.50 (1.37, 1.66)	1.43 × 10^−16^	0.607	1.53 (1.39, 1.69)	4.48 × 10^−17^	0.587	1.44 (1.30, 1.59)	6.63 × 10^−13^	0.523	1.47 (1.33, 1.63)	1.39 × 10^−13^	0.578
FPG (mmol/l)	2.32 (1.66, 3.23)	7.87 × 10^−7^	0.261	2.06 (1.48, 2.88)	2.10 × 10^−5^	0.220	1.46 (1.01, 2.10)	0.0435	0.004	1.31 (0.904, 1.88)	0.155	−0.031
HbA_1c_ (mmol/mol)	1.06 (1.03, 1.10)	2.77 × 10^−4^	0.182	1.06 (1.03, 1.09)	6.29 × 10^−4^	0.173	1.05 (1.01, 1.08)	6.19 × 10^−3^	0.099	1.04 (1.01, 1.08)	0.00910	0.077
2hPG (mmol/l)	–	–	–	–	–	–	1.35 (1.21, 1.49)	1.71 × 10^−8^	0.303	1.34 (1.21, 1.48)	4.20 × 10^−8^	0.310
PS (SD)	–	–	–	1.49 (1.29, 1.72)	4.31 × 10^−8^	0.268	–	–	–	1.48 (1.28, 1.71)	1.06 × 10^−7^	0.277

‘Clinical variables’ refers to: age, sex, parental diabetes, modified BMI *z* score and FPG for the youth cohort

NRI is calculated for each predictor by comparing the model including the predictor with a model that does not include the predictor

Db, diabetic with regard to parental diabetes; F, female; M, male

**Table 3:** Results of survival, AUC and NRI analyses for the birth cohort

Variable	Sex, parental diabetes (AUC=0.597)			Sex, parental diabetes + PS (AUC=0.683)			Sex, parental diabetes, birthweight (AUC=0.613)			Sex, parental diabetes, birthweight + PS (AUC=0.685)		
	HR (95% CI)	*p* value	NRI	HR (95% CI)	*p* value	NRI	HR (95% CI)	*p* value	NRI	HR (95% CI)	*p* value	NRI
Sex (F/M)	1.18 (0.974, 1.43)	0.0915	0.090	1.18 (0.972, 1.43)	0.0947	0.090	1.15 (0.951, 1.40)	0.146	0.090	1.14 (0.939, 1.38)	0.184	0.090
Mother diabetic/NonDb	7.28 (5.00, 10.6)	6.79 × 10^−30^	0.212	7.86 (5.39, 11.5)	1.12 × 10^−30^	0.209	7.40 (5.08, 10.77)	4.00 × 10^−30^	0.349	7.95 (5.44, 11.6)	5.20 × 10^−31^	0.317
Mother unknown/NonDb	0.871 (0.711, 1.07)			0.887 (0.724, 1.09)			0.875 (0.715, 1.07)			0.886 (0.723, 1.09)		
Father diabetic/NonDb	3.93 (2.36, 6.54)			3.98 (2.39, 6.64)			3.98 (2.38, 6.65)			4.05 (2.42, 6.78)		
Father unknown/NonDb	1.01 (0.818, 1.26)			1.07 (0.863, 1.33)			0.977 (0.787, 1.21)			1.03 (0.829, 1.28)		
Low birthweight	–	–	–	–	–	–	1.56 (1.24, 1.96)	6.11 × 10^−4^	0.164	1.16 (0.877, 1.53)	5.99 × 10^−4^	0.164
High birthweight	–	–	–	–	–	–	1.17 (0.887, 1.55)			1.57 (1.25, 1.97)		
PS (SD)	–	–	–	1.48 (1.35, 1.63)	2.83 × 10^−16^	0.345	–	–	–	1.48 (1.35, 1.63)	2.77 × 10^−16^	0.362

NRI is calculated for each predictor by comparing the model including the predictor with a model that does not include the predictor

Db, diabetic with regard to parental diabetes; F, female; M, male

### AUC analyses

We conducted AUC analyses to evaluate the predictive accuracy of models containing combinations of clinical variables and the PS. In the adult cohort, the AUC for the model with age and sex was 0.590 (95% CI 0.566, 0.615); with the PS, 0.619 (95% CI 0.596, 0.643); the difference in AUC (i.e. ∆AUC) was 0.029 (*p*=0.003). In the youth cohort, corresponding AUCs were 0.625 (95% CI 0.587, 0.663) and 0.682 (95% CI 0.648, 0.716); the ∆AUC was 0.057 (*p*=3.96 × 10^−4^). In the birth cohort, AUC for the model with sex was 0.537 (95% CI 0.512, 0.562); with the PS, 0.638 (95% CI 0.610, 0.666); the ∆AUC was 0.101 (*p*<10^−5^).

Though the PS was strongly associated with incident type 2 diabetes, the improvement in AUC compared with clinical variables alone was modest. In the adult cohort, AUC for the full clinical model was 0.728 (95% CI 0.706, 0.750); with the PS, 0.735 (95% CI 0.714, 0.757); and the ∆AUC was 0.007 (*p*=0.023) (Table 1). In the youth cohort, AUC for the full clinical model was 0.805 (95% CI 0.778, 0.832); with the PS, 0.812 (95% CI 0.785, 0.839); and the ∆AUC was 0.007 (*p*=0.173) (Table 2). For the birth cohort, the increment in AUC with addition of the PS was greater: the AUC for the model including clinical variables was 0.613 (95% CI 0.582, 0.644); with the PS, 0.685 (95% CI 0.657, 0.713); the ∆AUC was 0.071 (*p*<10^−5^) (Table 3).

### NRI analyses

**Fig. 2:**
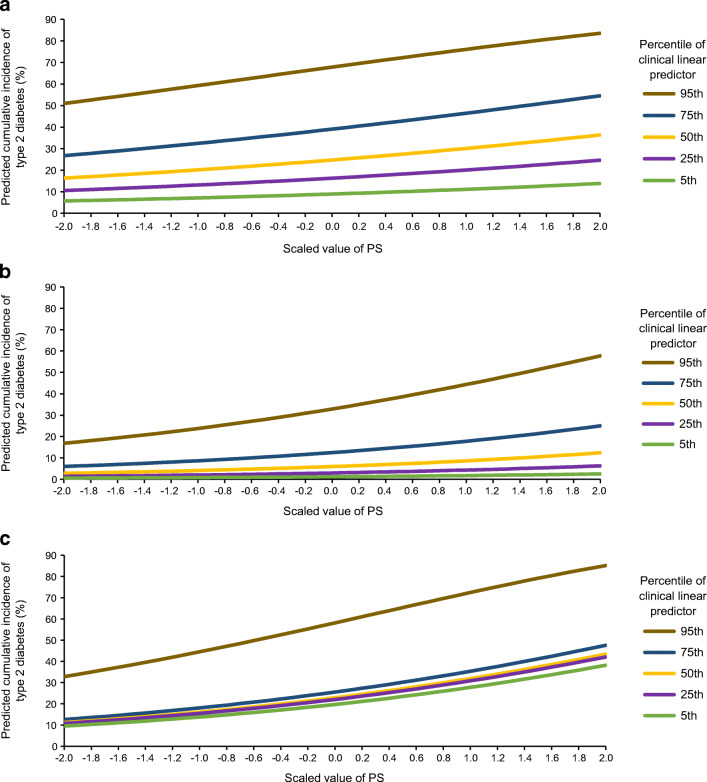
Predicted cumulative incidence of type 2 diabetes for the scaled DIAGRAM 2018 PS and specified percentiles of the clinical linear predictor. Cumulative incidence was calculated for various combinations of the PS and clinical risk (based on the linear predictor derived from the clinical variables in the models). Across all cohorts, greater type 2 diabetes PS and greater percentiles of clinical linear predictor were both directly and separately associated with predicted cumulative incidence of type 2 diabetes. (**a**) Predicted cumulative incidence of type 2 diabetes over 10 years of follow-up in the adult cohort: clinical variables include age, sex, parental diabetes, BMI, FPG, HbA_1c_. At 10 year follow-up, 504 individuals had developed type 2 diabetes and 635 remained at risk. (**b**) Predicted cumulative incidence of type 2 diabetes over 10 years of follow-up in the youth cohort: clinical variables include age, sex, parental diabetes, modified BMI *z* score, FPG, HbA_1c_. At 10 year follow-up, 152 had developed type 2 diabetes and 745 remained at risk. (**c**) Predicted cumulative incidence of type 2 diabetes over 30 years of follow-up in the birth cohort: clinical variables include sex, parental diabetes, birth weight. At 30 year follow-up, 340 had developed type 2 diabetes and 474 remained at risk

While AUC provides a measure of overall predictive accuracy, it does not fully capture the extent to which addition of a variable can affect individual risk estimates. To examine this, we calculated predicted cumulative incidence of type 2 diabetes according to PS for various levels of clinical risk. Across all cohorts, greater type 2 diabetes PS and greater percentiles of clinical linear predictor were both directly and separately associated with predicted cumulative incidence of type 2 diabetes (Fig. 2).

To further quantify the contribution of each variable to the model’s risk classification, we calculated the NRI of each variable. NRI quantifies the extent to which type 2 diabetes cases and non-cases are consequently reclassified upon inclusion of an additional variable. The NRI for adding the PS to clinical variables was 0.270 (95% CI 0.149, 0.392; 0.092 for events, 0.178 for nonevents) in the adult cohort (Table 1); in the youth cohort, 0.268 (95% CI 0.073, 0.464; 0.085 for events, 0.183 for nonevents) (Table 2); in the birth cohort, 0.362 (95% CI 0.222, 0.502; 0.106 for events, 0.256 for nonevents) (Table 3). In comparison, the NRI for HbA_1c_ was 0.267 in the adult cohort and 0.173 in the youth cohort.

### Additional genotypic analyses

The effects of some variants strongly associated with type 2 diabetes in this Indigenous study population were not captured in the DIAGRAM 2018 PS. To address this, we assessed the contribution of genotypes for *KCNQ1* rs2237895 (which exhibits parent-of-origin effects) and *ABCC8* rs1272388614 in the adult cohort. For each genotype, associations were significant; however, they contributed modestly to the model of clinical variables and the PS, as assessed by AUC and NRI analyses (ESM Table 13).

### Decision curve analyses

We employed decision curve analyses to estimate the net benefit of including the PS at a range of threshold probabilities (i.e. minimum probabilities of disease that would warrant intervention). When the costs of false-positives are low (i.e. as *p*_*t*_ approaches 0), population-wide interventions may be favoured; thus, screening by clinical or genetic means would have little net benefit. When false-positive costs are higher (i.e. at higher *p*_*t*_ values), net clinical benefit can be increased by screening to target the intervention to higher-risk individuals.

**Fig. 3:**
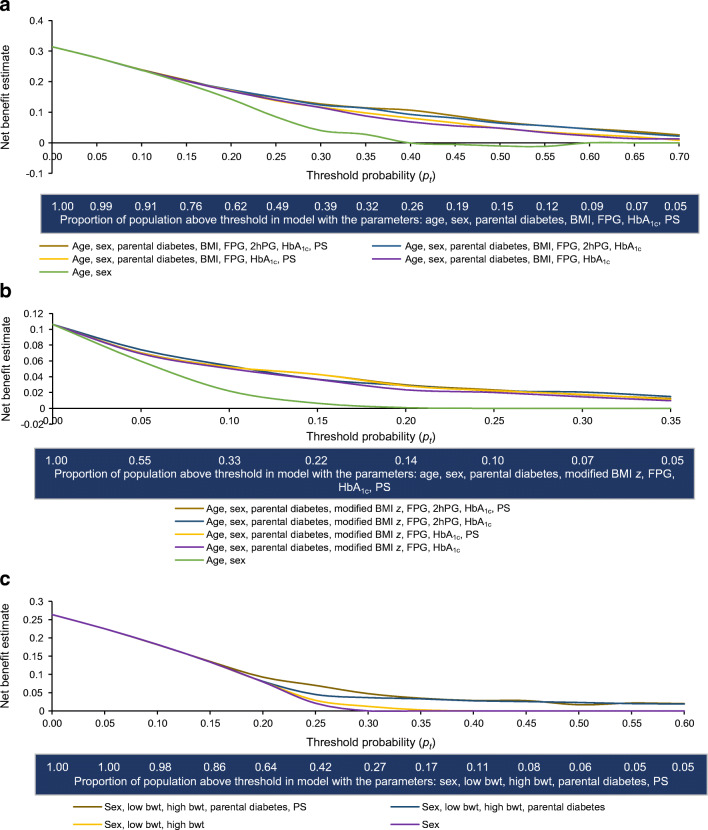
Net benefit of predictive models with or without the DIAGRAM 2018 PS for type 2 diabetes prediction. For each model in each cohort, the net benefit is plotted on the *y*-axis against the *p*_*t*_ for implementing an intervention on the *x*-axis. The proportion of the population that would be selected at each *p*_*t*_ is shown below the *x*-axis. (**a**) In the adult cohort, the net benefit of including the PS in addition to clinical variables was most pronounced at *p*_*t*_ values 0.3–0.5 (up to 18% improvement); this corresponded to 15–40% of the highest-risk individuals selected for the intervention. (**b**) In the youth cohort, the net benefit of including the PS was most pronounced at *p*_*t*_ values 0.05–0.35 (up to 21% improvement). (**c**) In the birth cohort, the net benefit of including the PS was most pronounced at *p*_*t*_ values 0.15–0.35 (up to 56% improvement). Bwt, birthweight

In the adult cohort, the net benefit of including the PS in addition to clinical variables was most pronounced at *p*_*t*_ values 0.3 to 0.5 (up to 18% improvement); this corresponded to 15–40% of the highest-risk individuals selected for the intervention (Fig. 3). In the youth cohort, the net benefit of including the PS was most pronounced at *p*_*t*_ values 0.05 to 0.35 (up to 21% improvement) (Fig. 3). In the birth cohort, the net benefit of including the PS was most pronounced at *p*_*t*_ values 0.15 to 0.35 (up to 56% improvement) (Fig. 3).

## Discussion

PSs potentially have utility for identification of individuals with higher risk of type 2 diabetes. Previous studies generally reported significant associations between type 2 diabetes PS and diabetes incidence and modest prediction improvement as measured by AUC: ∆AUC from 0.005 to 0.02 [1, 2, 3, 4, 5, 6]. A limited number of studies include measures of reclassification: continuous NRIs ranged from 0.044 to 0.285 [4, 5, 6]. Most previous studies have been done in European-ancestry populations, but some have been done in non-European populations, including Korean [5], African American [29] and Iranian [30]. Findings in these populations have generally been similar to those in European-ancestry groups. In the present study, the DIAGRAM 2018 PS was strongly statistically significant in predicting type 2 diabetes incidence in adult, youth and birth cohorts in an Indigenous study population from the Southwestern USA.

Results of AUC analyses are consistent with findings of previous studies: improvement in prediction contributed by the type 2 diabetes PS was statistically significant but modest. However, ∆AUC does not fully capture the contribution of a single variable to individual risk [31]. We calculated NRIs for individual variables to address this limitation. NRIs for the PS across all cohorts ranged from 0.2 to 0.3, which is considered intermediate power for identifying type 2 diabetes risk [24], and were comparable to those of commonly measured clinical variables (e.g. HbA_1c_ and FPG). Our findings are consistent with the evidence that for most chronic diseases PSs generally provide additional predictive information beyond that provided by traditional risk factors [32].

### Implications of decision curve analyses

Ultimately, clinical utility may depend on how the type 2 diabetes PS affects the decision to implement preventive interventions. Across adult, youth and birth cohorts, results of our decision curve analyses suggest modest increases in clinical benefit for using the PS at moderately stringent *p*_*t*_ values. There are few data on optimal *p*_*t*_ values for type 2 diabetes prevention: they depend upon preferences of individual patients and clinicians, healthcare system characteristics and the nature of the interventions considered. Many clinicians would recommend lifestyle prevention for individuals with impaired glucose regulation (e.g. FPG ≥5.5 mmol/l or HbA_1c_ ≥39 mmol/mol (5.7%)); in the adult cohort, the prevalence of impaired glucose regulation at baseline was 35%, and this would correspond to *p*_*t*_=0.32 (0.21–0.49 based on cumulative incidence). This is in the range in which our analyses suggest meaningful, albeit modest, improvement in clinical benefit from incorporating the type 2 diabetes PS.

Decision curve analysis assumes that the intervention will be equally effective regardless of how risk is determined. There are limited data on how type 2 diabetes PS affects response to preventive interventions. However, a study within the Diabetes Prevention Program Outcomes Study suggested that lifestyle and metformin interventions were both effective, even in those with greater type 2 diabetes PS [33].

### Construction of PS

While all type 2 diabetes PSs we examined were significantly associated with type 2 diabetes incidence across all cohorts, the DIAGRAM 2018 PS, derived from European-ancestry populations, performed slightly better than the others. While we have previously shown modest heterogeneity in effects of established type 2 diabetes variants between Europeans and this study population [7], the DIAGRAM 2018 PS even out-performed a population-specific variant PS with a comparable number of variants, derived by twofold cross-validation in the present population (*n*≈3850). The expectation is that a PS derived from a GWAS in a more closely matched ancestry group would perform better than one from a different ancestry group, if GWAS sample sizes are equal [34], but PSs derived in a large European-ancestry group can outperform ancestry-specific PSs when the sample size available for deriving the ancestry-specific PS is small [15]. In the present study, the DIAGRAM 2018 PS likely performed well due to the large sample size and extensive fine-mapping in the DIAGRAM type 2 diabetes meta-analysis. Achieving adequate sample sizes for GWASs to derive ancestry-specific PSs in Indigenous study populations is challenging, but many Indigenous populations have extensive linkage disequilibrium which may facilitate the ability of PSs to capture causal variants [35]. While further work is needed to optimise type 2 diabetes PSs across diverse populations, the present study suggests that PSs constructed using results of GWASs in larger populations may be suitable for translation across study populations in which well-powered GWASs are not available. Studies in additional populations are needed.

### Optimal age for preventive interventions

Genetic effects of the PSs with respect to type 2 diabetes incidence were greatest in the youth and birth cohorts. This is consistent with the hypothesis that genetic effects for many chronic diseases are strongest earlier in life [36], and consistent with the finding that familial recurrence risk of diabetes in this population is higher when it occurs at younger ages [37]. The present findings could also reflect the limited availability of phenotypic data for study participants at birth or young ages. However, when analysed without any clinical covariates, the HRs associated for the birth cohort (HR=1.47) and the youth cohort (HR=1.59) were significantly higher than that for the adult cohort (HR=1.31); tests for differences in the HRs between the adult and youth cohorts and adult and birth cohorts yielded *p=*0.037 and *p=*0.006, respectively, while differences between birth and youth cohorts were not significant (*p*=0.15). The improvements in AUC and net benefit upon adding the PS to clinical variables were greatest in the birth cohort. The use of type 2 diabetes PS at birth could be particularly beneficial as phenotypic manifestations of risk (e.g. hyperglycaemia and obesity) are less apparent. However, some relevant clinical measures that may be readily obtained at birth (e.g. birth length for calculation of adiposity measures) were not available in the present study. In adults, there is strong evidence that type 2 diabetes can be prevented by lifestyle modification, pharmacologic treatment or bariatric surgery, but there are few data on the efficacy of preventive efforts initiated in youth or infancy [38]. Thus, while our analyses suggest that the type 2 diabetes PS has the strongest contribution to prediction of type 2 diabetes incidence in the birth cohort, adults may be a more appropriate target population for preventive interventions in the near term.

### Future research

This study shows that type 2 diabetes PSs, as currently constructed, can provide utility for assessing type 2 diabetes risk; as measured by NRI analyses, information from the PS for classifying type 2 diabetes risk is comparable to that from widely used clinical variables (e.g. HbA_1c_ and BMI) in this study population. Further optimisation of the PS is expected to provide better prediction in the future [30]. Such investigations could assess whether differences in population genetic characteristics, obesity and incidence of type 2 diabetes are paralleled by differences in performance of PSs. Results from the present study were derived from an Indigenous population from the Southwestern USA with a relatively high prevalence of type 2 diabetes.

Beyond the scientific issues, however, technical, logistical and cultural issues need consideration before PSs can be incorporated into clinical practice. For example, advances in laboratory methods and informatics are required to make PSs and risk algorithms available to clinicians and patients. Health economics studies are needed to investigate which clinical settings and constructions of type 2 diabetes PS would maximise net benefit for prediction of type 2 diabetes incidence. With such knowledge, more informed decisions about the use of genetic information in prevention of type 2 diabetes could be made.

## Supplementary information

ESM 1 (PDF 2.08 kb)

## Data Availability

The data that support the findings of this study are not publicly available due to privacy concerns. Data may be made available upon reasonable request; for more information, refer to dbGAP (https://www.ncbi.nlm.nih.gov/gap/) accession number phs002490.v1.p1.

## Abbreviations

DIAGRAM: Diabetes Genetics Replication And Meta-analysis consortium
DIAMANTE: Diabetes Meta-Analysis of Trans-Ethnic association studies consortium
FPG: Fasting plasma glucose
GWAS: Genome-wide association study
2hPG: 2 h plasma glucose
MAF: Minor allele frequency
NRI: Net reclassification improvement
PS: Polygenic score
*p*_*t*_: Threshold probability
