# Supplementary material for: The utility of a type 2 diabetes polygenic score in addition to clinical variables for prediction of type 2 diabetes incidence in birth, youth and adult cohorts in an Indigenous study population
Source: Diabetologia. 2023 Mar 2;66(5):847–60. doi: 10.1007/s00125-023-05870-2 (PMC10036431; doi:10.1007/s00125-023-05870-2)
Supplement: Supplementary file 1 — (PDF 2.08 kb) [file 125_2023_5870_MOESM1_ESM.pdf]

Supplementary Appendix

Supplement to: Wedekind LE, Mahajan A, Hsueh W-C, et al. The utility of a type 2 diabetes polygenic score in addition to clinical variables for prediction of T2D incidence in adult, youth, and birth cohorts in an Indigenous study population.

Table of contents

Supplementary methods **4**

Method S1. Genotypic data. 4

Method S2. Construction of type 2 diabetes polygenic scores. 5

Supplementary figures **6**

Figure S1. Calibration plots for models of clinical variables and the type 2 diabetes PS for birth, youth, and adult cohorts. 6

Figure S2. Cumulative incidence of T2D by decile of the DIAMANTE 2022 multi-ancestry composite PS, in adult cohort. 7

Figure S3. Cumulative incidence of T2D by decile of the population-specific variant PS, in adult cohort. 8

Figure S4. Plot of predicted cumulative incidence of T2D by scaled DIAMANTE 2022 multi-ethnic composite PS and various percentiles of clinical linear predictor, in adult cohort. 9

Figure S5. Plot of predicted cumulative incidence of T2D by scaled population-specific variant PS and various percentiles of clinical linear predictor, in adult cohort. 10

Figure S6. Net benefit of predictive models with or without PS for T2D prediction for DIAMANTE 2022 multi-ethnic composite PS over 10-year follow-up time, in adult cohort. 11

Figure S7. Net benefit of predictive models with or without PS for T2D prediction for population-specific variant PS, in adult cohort. 12

Figure S8. Cumulative incidence of T2D over specified years of follow-up, stratified by decile of linear predictor of clinical variables. 13

Supplementary tables **14**

Table S1. Summary of continuous and binary traits in adult, youth, and birth cohorts. 14

Table S2. Summary of continuous and binary traits in adult, youth, and birth cohorts, for cases versus non-cases. 15

Table S3. SNPs included in the construction of the AGEN 2020[7] PS. 16

Table S4. Summary statistics for SNPs included in the construction of the DIAGRAM 2018[8] PS. 21

Table S5. SNPs included in the PSs derived from various ancestry-specific GWAS meta-analyses in DIAMANTE 2022[9] (including the population-specific weight PS). 28

Table S6. SNPs included in the population-specific variant PS. 38

Table S7. Results of survival, area under the ROC curve (AUC), and net reclassification improvement (NRI) analyses for the adult cohort with DIAGRAM 2018 T2D PS[a], with the inclusion of lipids traits. 57

Table S8. Results of survival, area under the ROC curve (AUC), and net reclassification improvement (NRI) analyses for adult and youth cohorts with DIAGRAM 2018 T2D PS, without inclusion of HbA_{1c}. 58

Table S9. Cox regressions comparing associations of the DIAGRAM 2018 PS (N=293 SNPs) with T2D incidence after adjustment for the first 10 genetic principal components[a]: birth, youth, and adult cohorts. 59

Table S10. Comparing analyses of PS constructions in birth, youth, and adult cohorts. 60

Table S11. Cox regressions, AUC and NRI analyses of the multi-ancestry composite DIAMANTE 2022 PS: adult cohort (N=2333). 61

Table S12. Cox regressions, AUC and NRI analyses of the population-specific variant PS: adult cohort (N=2333). 61

Table S13. AUC analyses for additional genotypes with DIAGRAM 2018 PS: adult cohort 62
Supplementary appendix references 63

Supplementary methods

Method S1. Genotypic data.

A longitudinal study of diabetes was conducted in an Indigenous study population from the Southwestern US. 7701 of the participants in the longitudinal study had genotypes available: genotypes were generated for previous genome-wide association studies using a custom Axiom genome-wide array (Affymetrix, Santa Clara, CA) that was designed to capture common variation in members of this community (minor allele frequency i.e., MAF≥0.05, or ≥0.01 for coding variants), using methods described previously.[1] The custom array identified tag single-nucleotide polymorphisms (SNPs) for approximately 5 million variants; 515,692 SNPs were directly genotyped. This genotyping captured approximately 92% of variation with MAF≥0.05 and 50% of variation with MAF 1-5%.

Missing and ungenotyped variants were imputed with whole genome sequence data for 266 members of the study population as a reference panel using Impute 2.[2] Variants were excluded if imputation quality score<0.5 or MAF<0.01. A total of 4,589,902 variants were imputed.

Method S2. Construction of type 2 diabetes polygenic scores.

Method S2a. Cross-validating population-specific weights for the DIAMANTE 2022 PS.

To estimate population-specific weights for variants constituting the T2D PSs, we used cross-validation to reduce overfitting associated with deriving the weights in the same dataset in which they are applied. We used a 10-fold cross-validation procedure to estimate weights for the 287 DIAMANTE 2022 multi-ancestry composite PS's variants in this Indigenous population. The 7701 participants in the GWAS were randomly divided into 10 equally sized groups, and the weights for each group were calculated by analyzing the association of each variant with T2D in the other 9 groups. Associations were analyzed with prevalent T2D at last examination (2571 cases in 7659 with data available) with adjustment for age, sex, birth year and the first 5 genetic principal components (to account for population structure). Analyses were conducted with a linear mixed model, which was fit with SOLAR,[3] that accounted for pairwise relationships among individuals, estimated with BEAGLE as previously described.[1,4] T2D was analyzed as a continuous (0,1) variable, and the regression coefficient was converted to an odds ratio by the method of Haggstrom.[5] The population-specific weights, derived from the average logarithm of the odds ratio across all 10 sub-analyses, are shown in Table S5, along with weights for each ethnicity derived from the DIAMANTE 2022 meta-analyses.

Method S2b. Selecting variants for population-specific variant PS.

We also derived a population-specific variant T2D PS by selecting 287 T2D-associated variants from the 515,692 variants typed in the T2D GWAS in the Indigenous study population. For computational efficiency and to reduce overfitting with assessments over a large number of variants, we used 2-fold cross-validation in this situation, since lower values of k in k-fold cross-validation can help reduce overfitting when a large number of features is assessed.[6] The GWAS participants were randomly divided into two equally-sized groups, and a GWAS of T2D was conducted in each group (the "discovery" group) to select the variants and weights used to construct the PS for the other group (the "target" group). To reduce the influence of linkage disequilibrium among variants in close physical proximity, the genome was divided into bins of ~200kb using the k-means procedure (PROC FASTCLUS in SAS), resulting in 25,763 bins across the genome. Within each discovery group, the variant with the lowest p-value for association with T2D was taken within each bin, and the 287 variants with the lowest *p*-values representing each bin were taken for constructing the PS for the target group. We chose 287 variants for constructing this population-specific variant score to obtain a PS with number of variants and information comparable to those from the DIAMANTE 2022 and DIAGRAM 2018 PSs. To avoid loss of statistical power associated with further splitting of the sample, we did not attempt to optimize the number of variants; we also did not attempt further validation in additional samples. Thus, generalizability of this score is not certain. The variants selected for this population-specific variant PS are shown in Table S6.

Supplementary figures

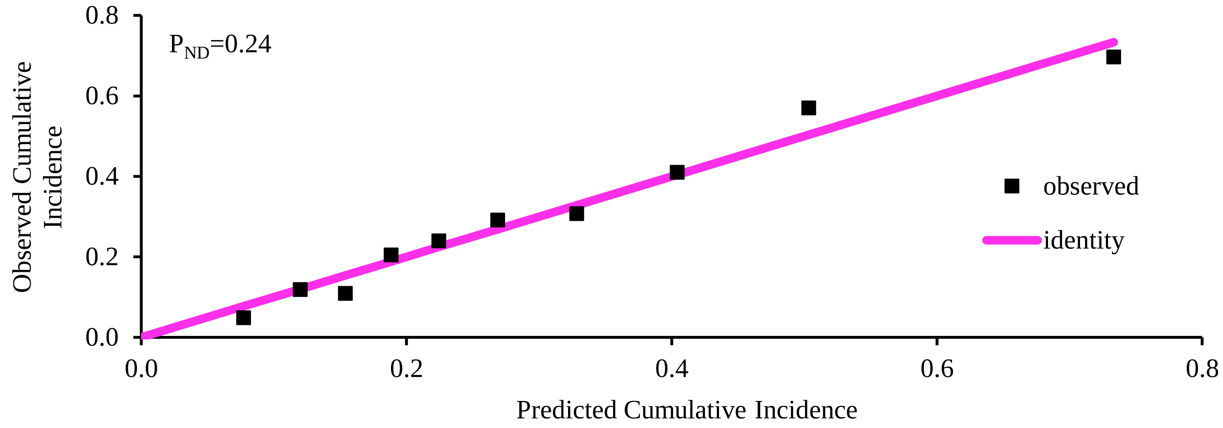

Figure S1a. Calibration plot for adult cohort with model of age, sex, parental diabetes, BMI, FPG, HbA1c, and PS.

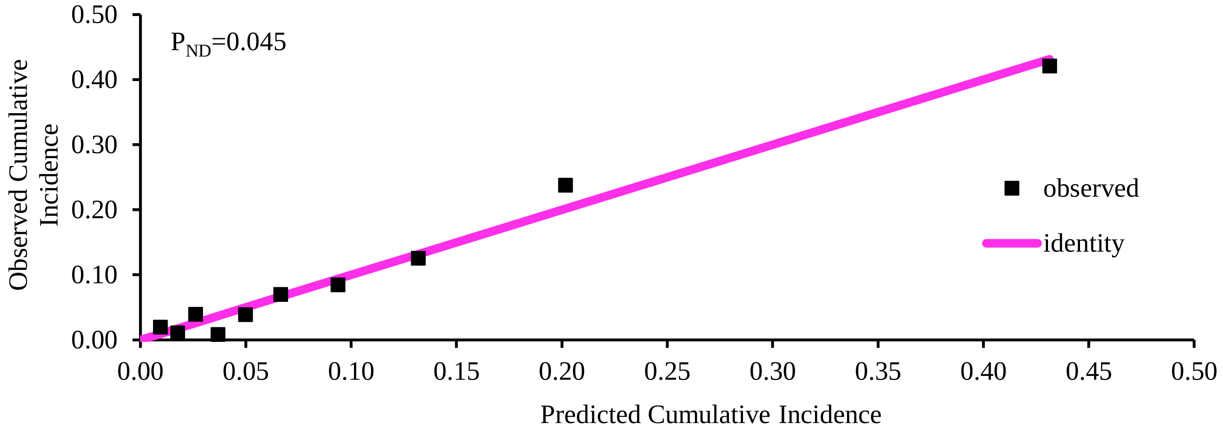

Figure S1b. Calibration plot for adult cohort with model of age, sex, parental diabetes, BMI z, FPG, HbA1c, and PS.

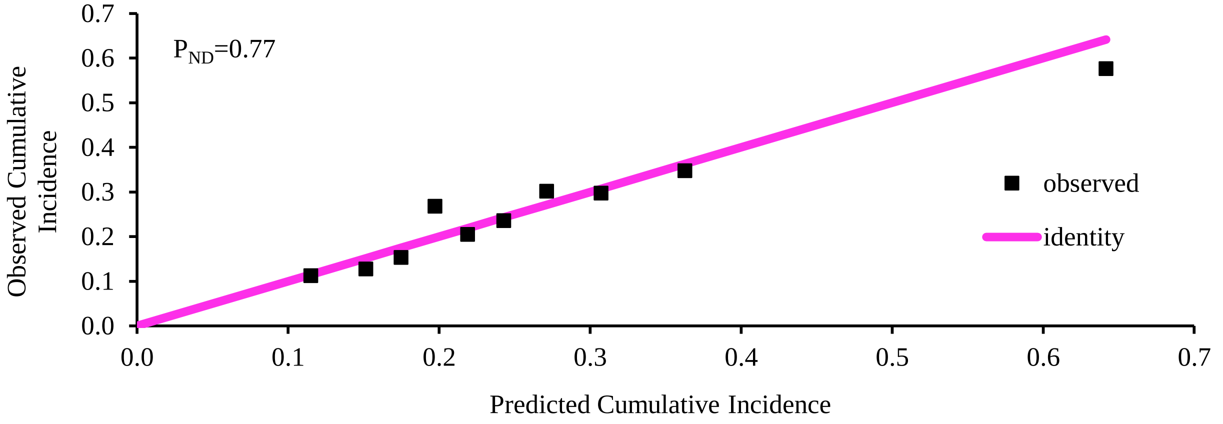

Figure S1c. Calibration plot for adult cohort with model of sex, parental diabetes, birth weight, and PS.

Figure S1. Calibration plots for models of clinical variables and the type 2 diabetes PS for birth, youth, and adult cohorts. P_{ND} is p-value from Nam-D'Agostino goodness of fit test

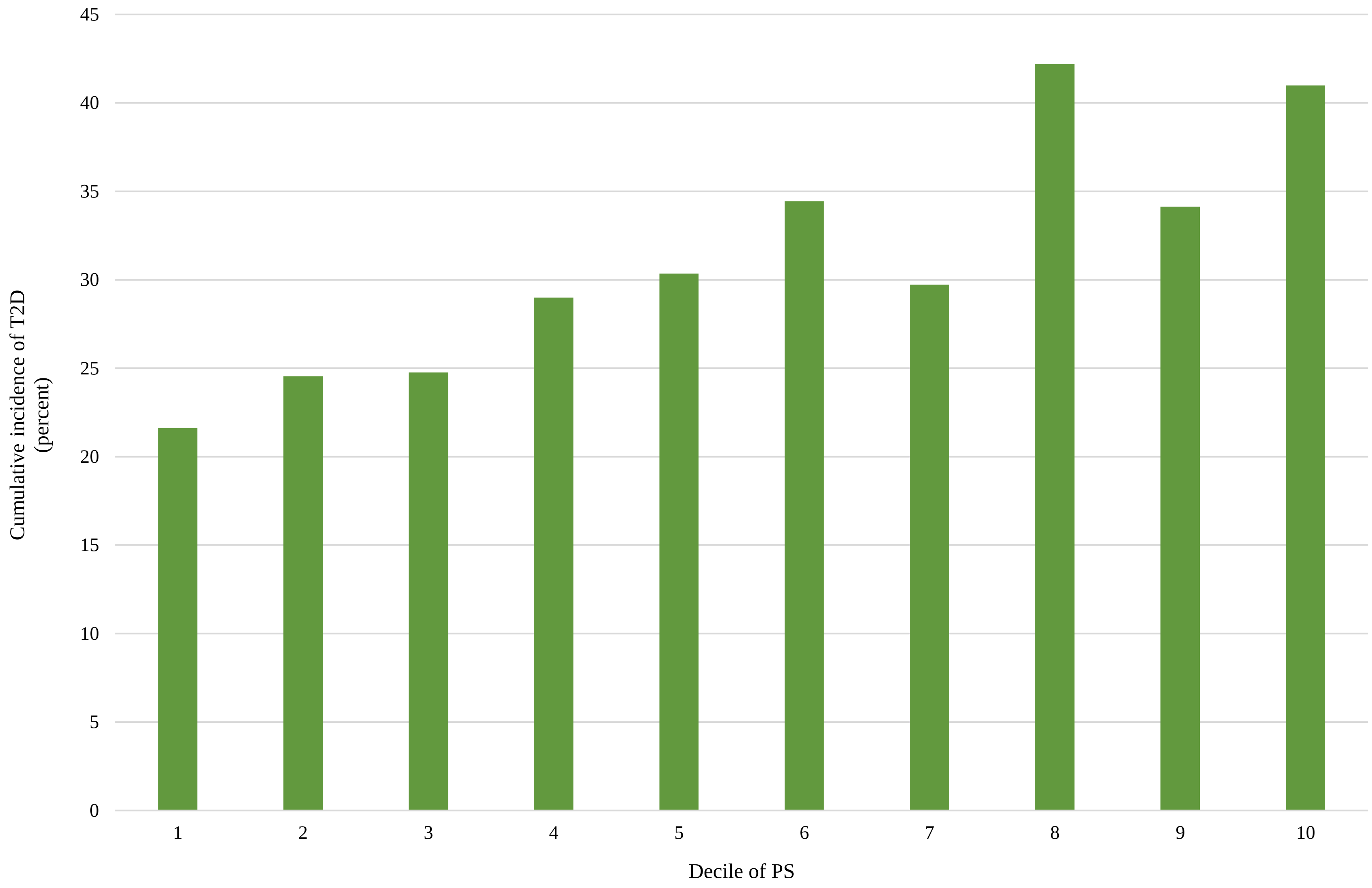

Figure S2. Cumulative incidence of T2D by decile of the DIAMANTE 2022 multi-ancestry composite PS, in adult cohort. At 10 years follow-up, 504 individuals had developed T2D and 635 remained at risk.

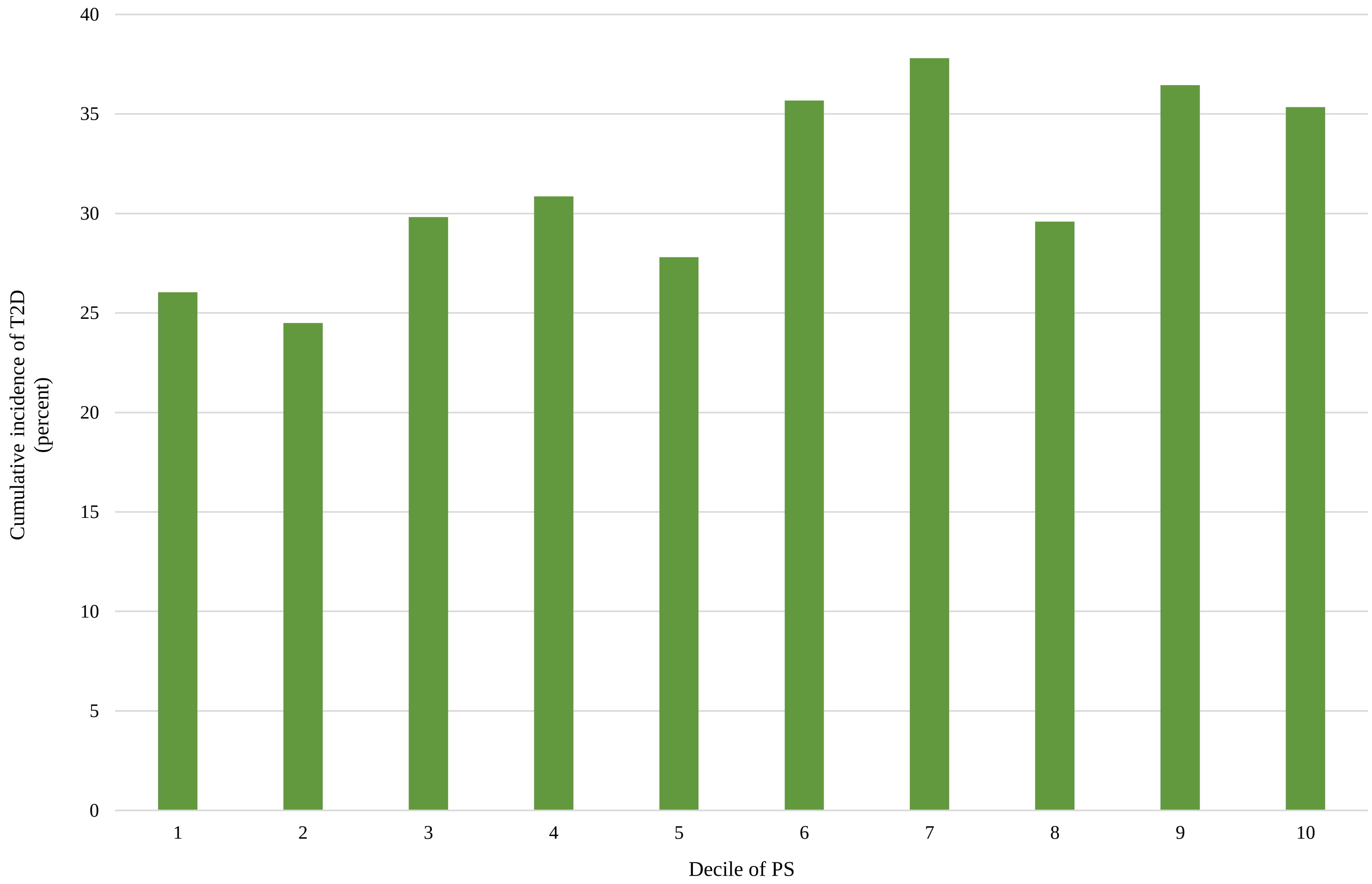

Figure S3. Cumulative incidence of T2D by decile of the population-specific variant PS, in adult cohort. At 10 years follow-up, 504 individuals had developed T2D and 635 remained at risk.

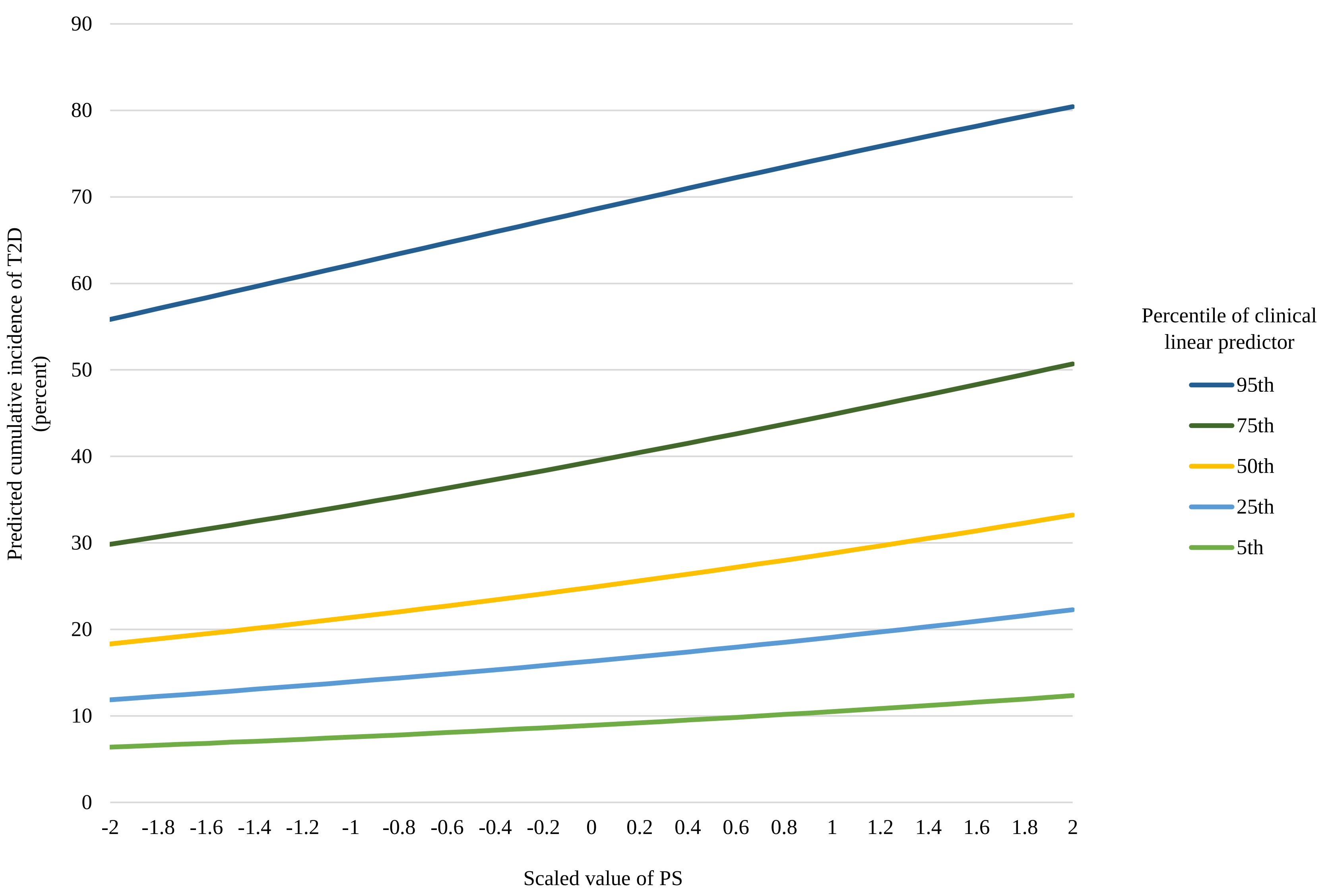

Figure S4. Plot of predicted cumulative incidence of T2D by scaled DIAMANTE 2022 multi-ethnic composite PS and various percentiles of clinical linear predictor, in adult cohort. At 10 years follow-up, 504 individuals had developed T2D and 635 remained at risk.

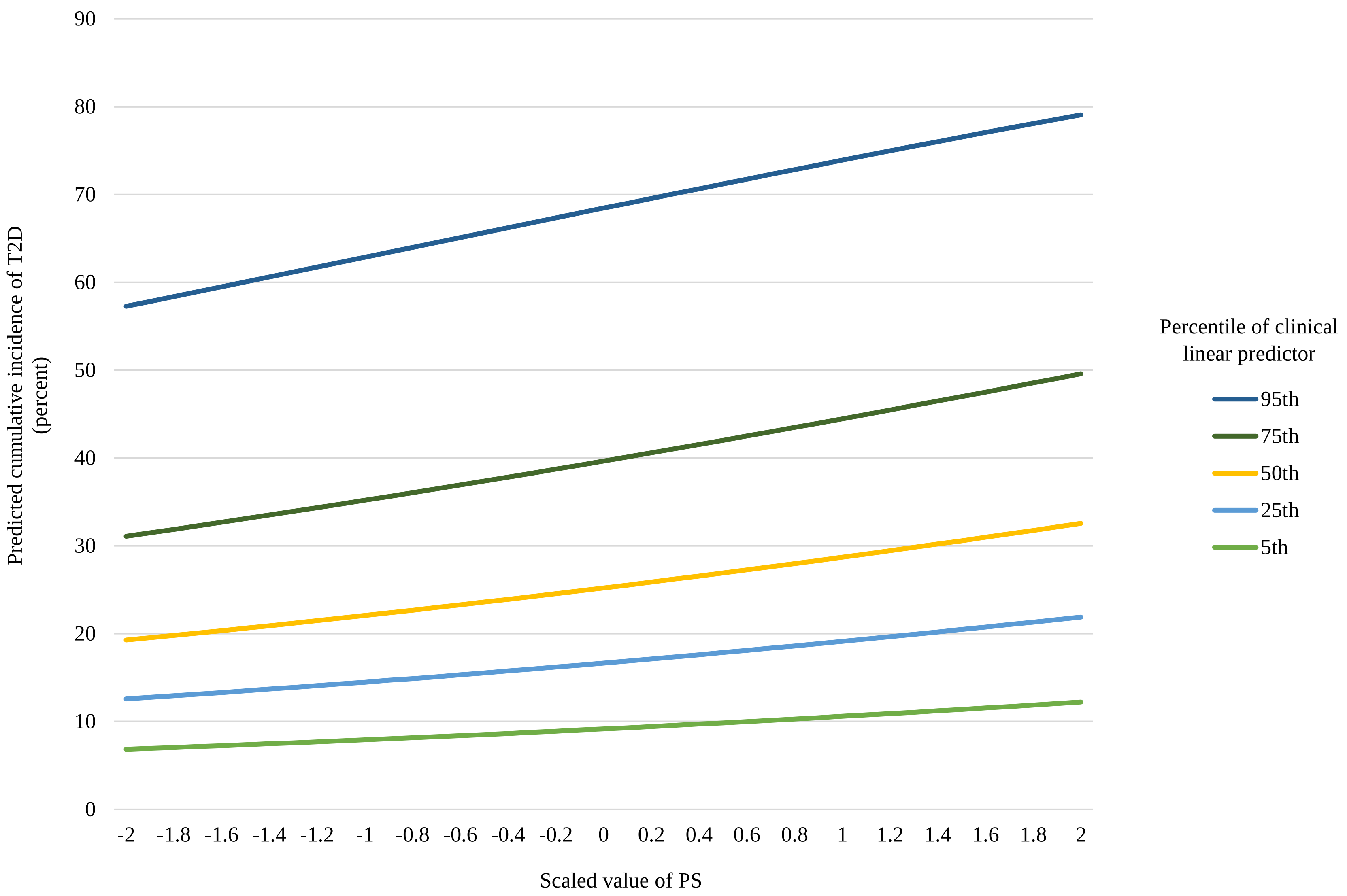

Figure S5. Plot of predicted cumulative incidence of T2D by scaled population-specific variant PS and various percentiles of clinical linear predictor, in adult cohort. At 10 years follow-up, 504 individuals had developed T2D and 635 remained at risk.

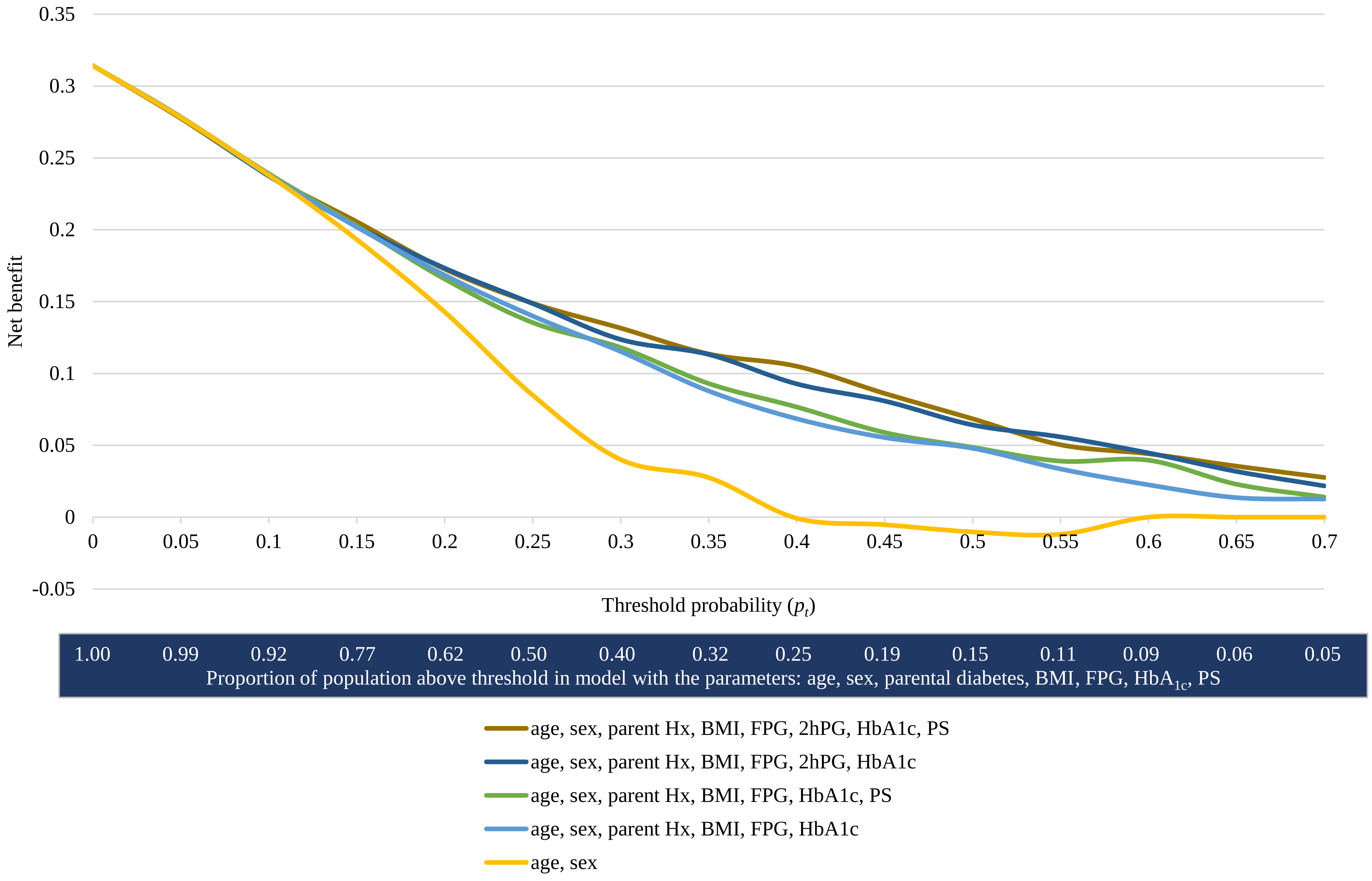

Figure S6. Net benefit of predictive models with or without PS for T2D prediction for DIAMANTE 2022 multi-ethnic composite PS over 10-year follow-up time, in adult cohort.

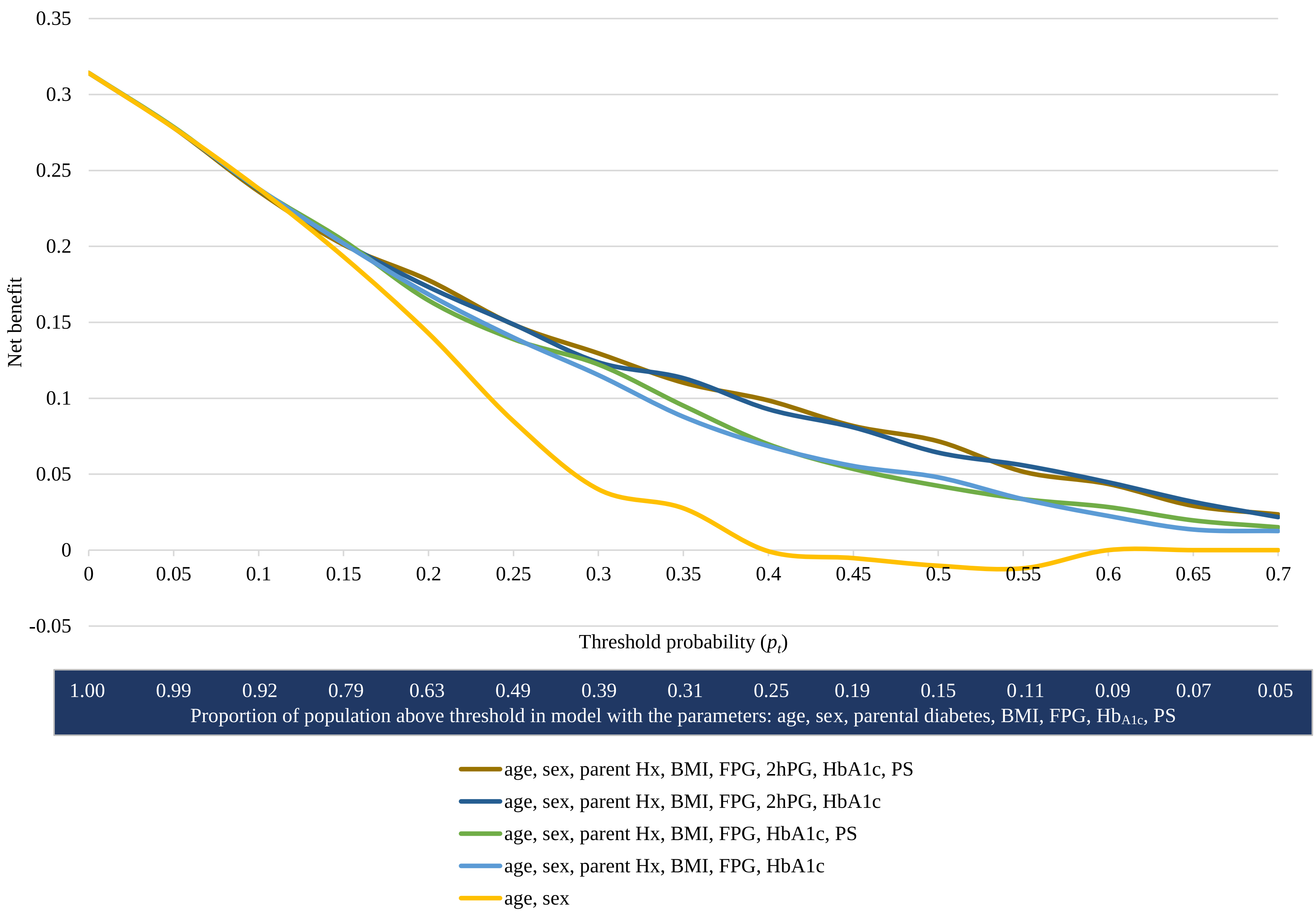

Figure S7. Net benefit of predictive models with or without PS for T2D prediction for population-specific variant PS, in adult cohort.

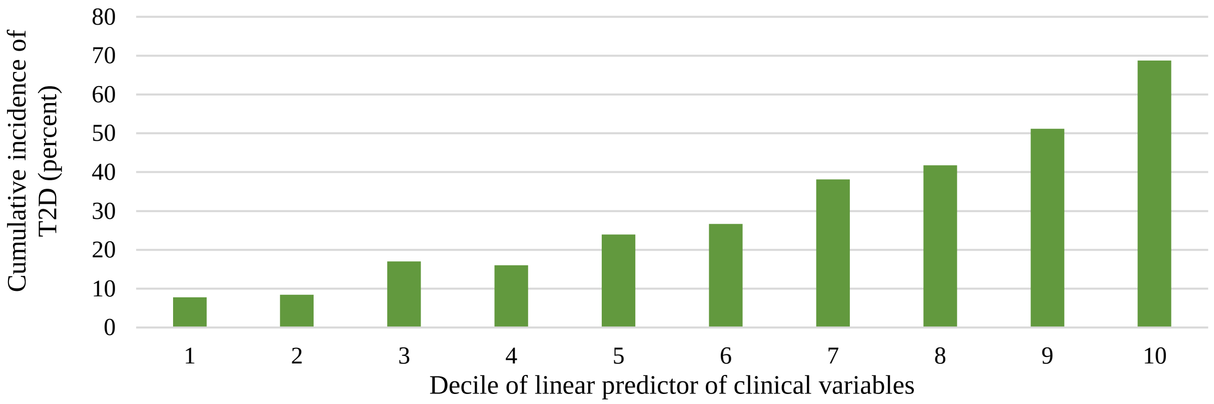

Figure S8a. Cumulative incidence of T2D over 10 years of follow-up, stratified by decile of linear predictor of clinical variables (age, sex, parental diabetes, BMI, FPG, HbA_{1c}), in the adult cohort. At 10 years follow-up, 504 individuals had developed T2D and 635 remained at risk.

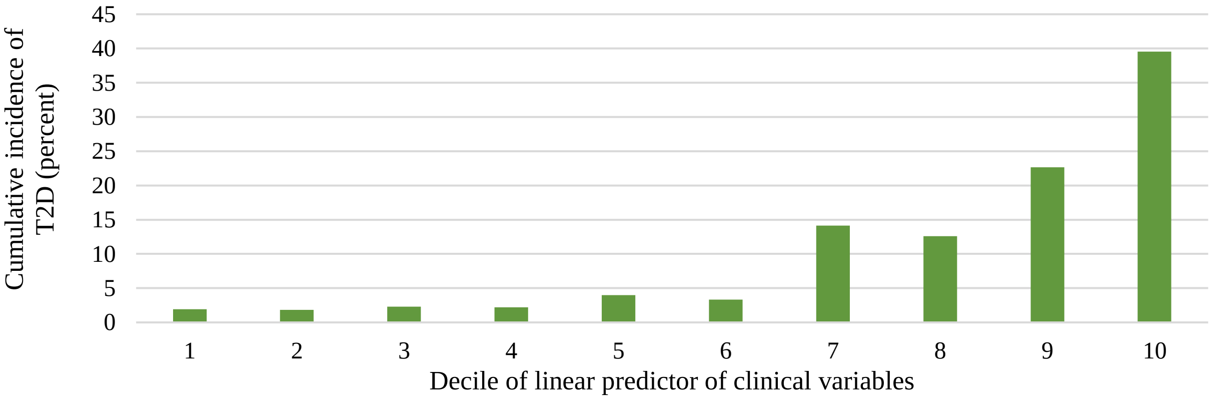

Figure S8b. Cumulative incidence of T2D over 10 years of follow-up, stratified by decile of linear predictor of clinical variables (age, sex, parental diabetes, modified BMI z, FPG, HbA_{1c}), in the youth cohort. At 10 years follow-up, 152 had developed T2D and 745 remained at risk.

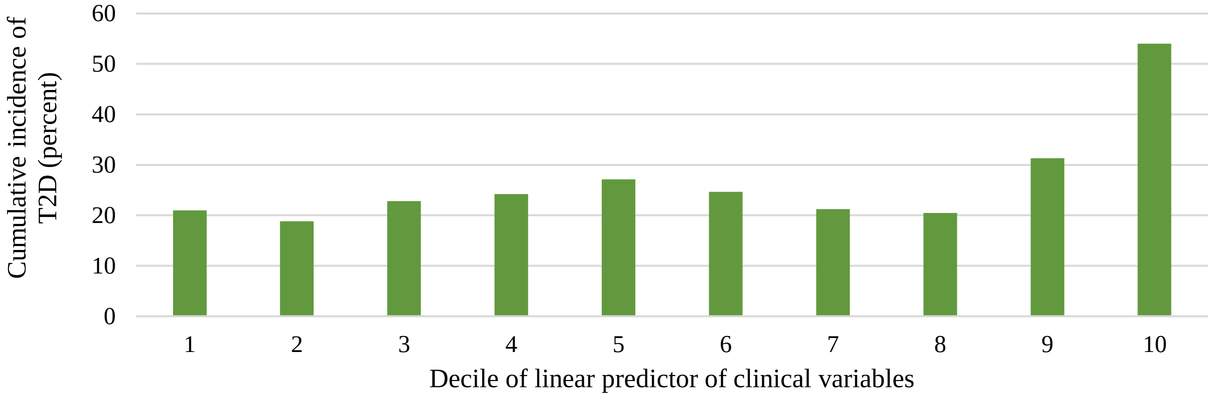

Figure S8c. Cumulative incidence of T2D over 30 years of follow-up, stratified by decile of linear predictor of clinical variables (sex, parental diabetes, birth weight), in the birth cohort. At 30 years follow-up, 340 had developed T2D and 474 remained at risk.

Figure S8. Cumulative incidence of T2D over specified years of follow-up, stratified by decile of linear predictor of clinical variables.

Supplementary tables

Table S1. Summary of continuous and binary traits in adult, youth, and birth cohorts.

Table S1a. Summary of continuous traits of participants in adult, youth, and birth cohorts.

	Birth cohort (N=2894)		Youth cohort (N=2229)		Adult cohort (N=2333)[c]	
**Variable**	**Mean**	**SD**	**Mean**	**SD**	**Mean**	**SD**
**follow-up time (years)**	21.28	7.81	7.99	4.34	7.15	4.45
**age (years)**			12.05	3.73	31.04	10.43
**BMI (kg/m^2)**			24.83	7.02	34.33	7.68
**FPG (mmol/l)**			4.93	0.40	5.21	0.54
**2hPG (mmol/l)**			5.64	1.28	6.29	1.68
**HbA$_{1c}$ (mmol/mol)**			31.75	4.21	33.84	5.04
**HbA$_{1c}$ (%)**			5.06	0.38	5.25	0.46
**birth weight (g)**	3456.86	532.09				
**unweighted DIAGRAM 2018 T2D PS[a]**	306.88	9.77	306.41	9.68	306.97	9.34

[a] The unweighted DIAGRAM 2018 PS is the sum of the number of T2D risk alleles across the 293 SNPs in the DIAGRAM 2018 PS (Table S4).

Table S1b. Summary of binary traits of participants in adult, youth, and birth cohorts.

	Birth cohort (N=2894)		Youth cohort (N=2229)		Adult cohort (N=2333)[c]	
**Variable**	**Frequency**	**Percent**	**Frequency**	**Percent**	**Frequency**	**Percent**
**sex female**	1569	54.22	1205	54.06	1395	59.79
**maternal diabetes prior to participant's entry into study**						
**with diabetes**	96	3.32	490	21.98	771	33.05
**without diabetes**	1837	63.48	1417	63.57	655	28.08
**diabetes status unknown**	961	33.21	322	14.45	907	38.88
**paternal diabetes prior to participant's entry into study**						
**with diabetes**	51	1.76	220	9.87	327	14.02
**without diabetes**	1015	35.07	764	34.28	350	15.00
**diabetes status unknown**	1828	63.17	1245	55.85	1656	70.98
**low birth weight (<3000g)**	493	17.04				
**high birth weight (≥4000g)**	391	13.51				

Table S2. Summary of continuous and binary traits in adult, youth, and birth cohorts, for cases versus non-cases.

Table S2a. Summary of continuous traits of participants in adult, youth, and birth cohorts.

	Birth cohort (N=2894)				Youth cohort (N=2229)				Adult cohort (N=2333)			
	Cases (N=438)		Non-cases (N=2456)		Cases (N=228)		Non-cases (N=2001)		Cases (N=640)		Non-cases (N=1693)	
**Variable**	**Mean**	**SD**	**Mean**	**SD**	**Mean**	**SD**	**Mean**	**SD**	**Mean**	**SD**	**Mean**	**SD**
**follow-up time (years)**	23.53	7.66	20.88	7.77	7.76	4.09	8.01	4.37	6.39	4.18	7.44	4.52
**age (years)**					13.89	3.89	11.84	3.65	32.85	10.07	30.36	10.48
**BMI (kg/m^2) (modified BMI *z* for youth)**					1.88	0.74	1.33	1.36	36.98	7.63	33.32	7.46
**FPG (mmol/l)**					5.19	0.49	4.90	0.38	5.51	0.58	5.10	0.48
**2hPG (mmol/l)**					6.64	1.60	5.53	1.18	7.21	1.78	5.94	1.51
**HbA_{1c} (mmol/mol)**					33.67	4.76	31.53	4.08	36.17	5.35	32.96	4.63
**HbA_{1c} (%)**					5.23	0.44	5.04	0.37	5.46	0.49	5.17	0.42
**birth weight (g)**	3384.28	591.51	3469.80	519.85								
**unweighted DIAGRAM 2018 T2D PS[a]**	309.97	9.24	306.33	9.77	309.73	8.84	306.034	9.70	306.97	9.34	306.30	9.30

[a] The unweighted DIAGRAM 2018 PS is the sum of the number of T2D risk alleles across the 293 SNPs in the DIAGRAM 2018 PS (Table S4).

Table S2b. Summary of binary traits of participants in adult, youth, and birth cohorts.

	Birth cohort (N=2894)				Youth cohort (N=2229)				Adult cohort (N=2333)			
	Cases (N=438)		Non-cases (N=2456)		Cases (N=228)		Non-cases (N=2001)		Cases (N=640)		Non-cases (N=1693)	
**Variable**	**Frequency**	**Percent**	**Frequency**	**Percent**	**Frequency**	**Percent**	**Frequency**	**Percent**	**Frequency**	**Percent**	**Frequency**	**Percent**
**sex female**	267	60.96	1302	53.01	141	61.84	1064	53.17	226	31.31	981	57.94
**maternal diabetes prior to participant's entry into study**												
**with diabetes**	33	7.53	63	2.57	93	40.79	397	19.84	238	37.19	533	31.48
**without diabetes**	405	92.47	2393	97.43	135	59.21	1604	80.16	402	62.81	1160	68.52
**diabetes status unknown**	187	42.69	774	31.51	42	18.42	280	13.99	284	44.38	623	36.80
**paternal diabetes prior to participant's entry into study**												
**with diabetes**	17	3.88	34	1.38	44	19.30	176	8.80	108	16.88	219	12.94
**without diabetes**	421	96.12	2422	98.62	184	80.70	1825	91.20	532	83.13	1474	87.06
**diabetes status unknown**	298	68.04	1530	62.30	117	51.32	1128	56.37	463	72.34	1193	70.47
**low birth weight (<3000g)**	105	23.97	388	15.80								
**high birth weight (≥4000g)**	62	14.16	329	13.40								

Table S3. SNPs included in the construction of the AGEN 2020[7] PS.

chr[a]	position[b]	SNP	a1[c]	a2	a1f[d]	OR[e]	*p*-value
**1**	20688352	rs60573766	C	T	0.6347	1.04	4.30×10^{-10}
**1**	39942242	rs371894931	CA	C	0.1572	1.06	2.68×10^{-11}
**1**	46244900	rs562138031	C	CT	0.7256	1.06	3.99×10^{-12}
**1**	51191935	rs11205766	T	A	0.9031	1.09	7.49×10^{-15}
**1**	64107893	rs2269245	G	A	0.8153	1.06	5.40×10^{-10}
**1**	177878933	rs532504	A	G	0.2133	1.06	7.39×10^{-12}
**1**	184014593	rs1327123	C	G	0.4596	1.04	7.00×10^{-9}
**1**	204474581	rs201297151	CAAAAAAAA	C	0.4404	1.04	3.37×10^{-8}
**1**	214155398	rs12403994	C	A	0.6228	1.05	6.05×10^{-12}
**1**	229642499	rs238763	T	A	0.6783	1.05	5.04×10^{-11}
**2**	632789	rs10634531	CTTG	C	0.9064	1.1	2.37×10^{-17}
**2**	27730940	rs1260326	C	T	0.4562	1.07	1.01×10^{-21}
**2**	45192080	rs12712928	C	G	0.4022	1.06	1.83×10^{-14}
**2**	60586707	rs243018	G	C	0.6656	1.06	1.55×10^{-15}
**2**	120231070	rs3731600	C	G	0.9675	1.13	6.86×10^{-9}
**2**	149568261	rs200576292	CT	C	0.6603	1.05	1.29×10^{-9}
**2**	165381518	rs75536691	A	G	0.976	1.2	1.16×10^{-15}
**2**	213687103	rs75179644	T	C	0.8992	1.08	5.36×10^{-10}
**2**	234191103	rs117809958	A	T	0.0181	1.25	1.98×10^{-15}
**3**	12385357	rs3963364	C	A	0.95	1.12	3.49×10^{-11}
**3**	23258614	rs11926494	G	A	0.82	1.12	2.69×10-37
**3**	63904715	rs67114627	AAG	A	0.6459	1.09	9.10×10^{-32}
**3**	114968018	rs6806156	T	C	0.6107	1.05	1.59×10^{-11}
**3**	121965199	rs9859381	G	T	0.486	1.04	2.86×10^{-9}
**3**	123174832	rs60054445	C	T	0.6643	1.05	5.62×10^{-12}
**3**	124921920	rs12497133	A	G	0.3231	1.04	1.14×10^{-8}
**3**	152382352	rs1850421	A	C	0.2777	1.05	1.41×10^{-9}
**3**	170643788	rs201018682	T	C	0.8162	1.06	1.01×10^{-11}
**3**	185495320	rs13092876	A	G	0.3122	1.13	1.91×10^{-66}
**3**	186649931	rs11332772	C	CG	0.5571	1.05	1.07×10^{-10}
**3**	187698333	rs13086331	T	C	0.8187	1.05	7.29×10^{-9}
**3**	195830310	rs9866168	T	A	0.6394	1.05	1.55×10^{-9}
**4**	1254535	rs7656416	C	T	0.6833	1.11	9.01×10^{-42}
**4**	1784605	rs6831006	G	C	0.2736	1.07	2.92×10^{-16}
**4**	6303731	rs147834269	G	A	0.9784	1.23	9.10×10^{-12}
**4**	45186139	rs10938398	A	G	0.2921	1.05	3.84×10^{-10}
**4**	71844118	rs28599782	A	G	0.209	1.07	4.64×10^{-16}
**4**	85339618	rs117624659	T	C	0.9765	1.23	1.95×10^{-16}
**4**	153520279	rs10011838	G	A	0.493	1.08	1.43×10^{-27}
**5**	14768092	rs6885132	C	G	0.556	1.04	2.59×10^{-9}

**5**	36257018	rs16902871	G	A	0.149	1.06	3.34×10^{-9}
**5**	50079603	rs74334916	C	A	0.0748	1.07	4.33×10^{-8}
**5**	51751574	rs12109081	T	A	0.3608	1.04	1.08×10^{-8}
**5**	55810305	rs256904	T	A	0.4923	1.08	3.56×10^{-29}
**5**	74574984	rs2126736	A	G	0.4279	1.04	1.84×10^{-8}
**5**	95848503	rs6556925	C	A	0.4155	1.04	3.12×10^{-9}
**5**	133864599	rs329122	A	G	0.3865	1.04	2.22×10^{-8}
**5**	176513896	rs3135911	A	C	0.4324	1.05	1.51×10^{-12}
**6**	7231843	rs9379084	G	A	0.7986	1.07	2.20×10^{-14}
**6**	20683164	rs9350271	A	G	0.4225	1.21	4.96×10^{-183}
**6**	31026236	rs76541615	T	G	0.7959	1.08	1.08×10^{-17}
**6**	34214670	rs4711389	A	G	0.8925	1.1	7.28×10^{-17}
**6**	39046644	rs742762	A	C	0.7118	1.08	1.79×10^{-22}
**6**	50787459	rs62405419	T	G	0.2682	1.05	3.79×10^{-9}
**6**	117996631	rs80196932	T	C	0.7859	1.06	7.57×10^{-13}
**6**	126964510	rs4273712	G	A	0.4688	1.05	2.56×10^{-12}
**6**	131954797	rs7739842	G	T	0.3564	1.05	1.55×10^{-11}
**6**	137294771	rs35389258	T	TA	0.45	1.05	9.49×10^{-14}
**6**	139205386	rs9376382	C	T	0.5989	1.04	1.50×10^{-8}
**6**	143056556	rs9390022	T	C	0.7997	1.05	6.35×10^{-9}
**7**	13886654	rs7787720	T	C	0.4209	1.06	2.26×10^{-15}
**7**	14898282	rs17168486	T	C	0.4181	1.07	8.23×10^{-22}
**7**	28219310	rs3735567	G	A	0.7776	1.06	3.05×10^{-12}
**7**	44174857	rs2908279	G	T	0.627	1.05	8.42×10^{-11}
**7**	55984953	rs565050730	GA	G	0.3341	1.04	4.43×10^{-8}
**7**	69189726	rs610930	A	G	0.2868	1.07	1.38×10^{-19}
**7**	69696905	rs12698877	G	A	0.3362	1.07	6.96×10^{-22}
**7**	89752238	rs62469016	C	G	0.2231	1.07	1.52×10^{-15}
**7**	93107093	rs2074120	A	C	0.323	1.04	8.38×10^{-9}
**7**	102336979	rs75990271	T	C	0.8152	1.07	3.22×10^{-11}
**7**	126526991	rs117737118	G	A	0.0934	1.18	3.31×10^{-31}
**7**	127253550	rs2233580	T	C	0.0858	1.34	2.73×10^{-132}
**7**	127761917	rs61342118	A	C	0.0831	1.34	4.78×10^{-105}
**7**	140579350	rs71170768	TA	T	0.3159	1.07	2.17×10^{-10}
**7**	157024510	rs1182444	G	A	0.4947	1.05	1.67×10^{-12}
**8**	17927609	rs34642578	T	C	0.0533	1.09	1.60×10^{-9}
**8**	36832310	rs56687477	A	G	0.3227	1.05	1.53×10^{-10}
**8**	37391203	rs4739515	G	C	0.5383	1.05	1.68×10^{-11}
**8**	38343012	rs328301	T	C	0.3278	1.04	4.11×10^{-8}
**8**	41512648	rs33981001	GGT	G	0.3888	1.08	5.27×10^{-28}
**8**	73503743	rs349359	C	A	0.2424	1.04	3.05×10^{-8}
**8**	75214398	rs149265787	G	A	0.0239	1.14	5.68×10^{-10}
**8**	95960886	rs896852	G	T	0.2998	1.04	6.42×10^{-9}

**8**	118184783	rs13266634	C	T	0.5857	1.12	3.73×10^{-67}
**8**	126471274	rs60089934	A	G	0.3777	1.04	3.33×10^{-9}
**8**	132879795	rs73708054	C	T	0.2523	1.04	4.41×10^{-8}
**9**	1032567	rs1016565	A	G	0.4209	1.04	2.18×10^{-8}
**9**	4290085	rs4237150	C	G	0.4262	1.07	4.46×10^{-27}
**9**	22132878	rs10965248	T	C	0.563	1.2	4.42×10^{-164}
**9**	81917127	rs1328412	T	C	0.9448	1.1	6.41×10^{-11}
**9**	84308948	rs2796441	G	A	0.3897	1.08	1.43×10^{-28}
**9**	98278413	rs113154802	C	T	0.8885	1.06	3.51×10^{-8}
**9**	107597527	rs201375651	CA	C	0.3948	1.04	2.56×10^{-8}
**9**	136149500	rs529565	C	T	0.4445	1.04	1.68×10^{-10}
**9**	139246588	rs376993806	G	A	0.8821	1.14	4.52×10^{-26}
**10**	12309139	rs11257657	G	C	0.4832	1.12	9.77×10^{-62}
**10**	23487778	rs77065181	A	G	0.0468	1.09	1.57×10^{-8}
**10**	63712602	rs141583966	G	GGTGT	0.9093	1.08	7.65×10^{-10}
**10**	64976133	rs148928116	T	TA	0.7945	1.06	2.53×10^{-13}
**10**	71273357	rs1955163	G	A	0.5409	1.05	1.68×10^{-11}
**10**	77323643	rs34907385	C	T	0.4914	1.05	5.37×10^{-12}
**10**	80951130	rs34204798	C	CG	0.5681	1.06	5.02×10^{-19}
**10**	89684214	rs1236816	A	C	0.499	1.04	4.31×10^{-10}
**10**	93592703	rs147689733	T	C	0.0209	1.31	1.21×10^{-27}
**10**	94435673	rs35906730	G	A	0.2645	1.15	1.27×10^{-71}
**10**	95009180	rs565236700	C	T	0.0235	1.23	5.54×10^{-19}
**10**	99056921	rs10736116	C	G	0.3058	1.05	9.21×10^{-11}
**10**	112678657	rs7895872	T	G	0.5787	1.05	1.37×10^{-11}
**10**	114754088	rs7901695	C	T	0.0382	1.32	8.18×10^{-62}
**10**	122929493	rs10886863	C	T	0.6641	1.06	5.28×10^{-17}
**10**	124150342	rs112820281	C	CTGGA	0.4098	1.05	1.42×10^{-10}
**11**	2203154	rs11043003	C	T	0.0819	1.11	1.03×10^{-16}
**11**	2858546	rs2237897	C	T	0.6321	1.28	1.88×10^{-245}
**11**	17415190	rs4148646	C	G	0.3846	1.08	1.70×10^{-26}
**11**	27729505	rs4922793	A	G	0.5655	1.04	1.62×10^{-10}
**11**	69462642	rs602652	A	G	0.8088	1.06	5.32×10^{-9}
**11**	72463435	rs7109575	G	A	0.9448	1.15	5.46×10^{-21}
**11**	92708710	rs10830963	G	C	0.421	1.04	4.49×10^{-8}
**12**	4381981	rs7304270	C	T	0.7614	1.07	1.04×10^{-12}
**12**	27963402	rs3751236	G	A	0.6717	1.07	6.58×10^{-21}
**12**	31441179	rs80234489	C	A	0.1699	1.11	4.33×10^{-32}
**12**	50269863	rs77978149	T	C	0.0898	1.08	5.70×10^{-9}
**12**	66232810	rs2583934	T	G	0.3396	1.06	4.95×10^{-16}
**12**	71449521	rs7313668	T	G	0.3742	1.05	4.91×10^{-11}
**12**	97850215	rs10860209	C	A	0.589	1.04	5.67×10^{-9}
**12**	108629780	rs1426371	G	A	0.4911	1.05	7.76×10^{-12}

**12**	111836771	rs149212747	A	AC	0.7948	1.07	2.07×10^{-11}
**12**	112736118	rs77768175	A	G	0.8068	1.07	1.39×10^{-10}
**12**	114123722	rs7307263	G	C	0.4272	1.04	3.64×10^{-8}
**12**	118400856	rs111246699	A	G	0.2572	1.06	1.54×10^{-15}
**12**	121363506	rs118074491	G	A	0.0323	1.21	8.83×10^{-20}
**13**	22589883	rs9316706	A	G	0.3505	1.04	3.33×10^{-9}
**13**	26781367	rs568052023	C	CA	0.5812	1.07	2.62×10^{-22}
**13**	33557173	rs7983505	T	A	0.1572	1.08	3.22×10^{-18}
**13**	51088809	rs123378	G	A	0.1951	1.05	2.22×10^{-10}
**13**	80707429	rs1215468	A	G	0.7185	1.09	1.32×10^{-31}
**13**	91949562	rs9515905	A	G	0.831	1.08	7.24×10^{-18}
**14**	24878370	rs12437434	C	T	0.7127	1.05	1.02×10^{-9}
**14**	38809661	rs61975988	A	G	0.4569	1.04	1.97×10^{-9}
**14**	77382503	rs58524310	G	A	0.3268	1.05	8.41×10^{-11}
**14**	101255172	rs73347525	A	G	0.7556	1.06	7.46×10^{-11}
**14**	103237952	rs55700915	A	G	0.4344	1.04	1.50×10^{-8}
**15**	28546173	rs76704029	T	C	0.7322	1.06	3.39×10^{-8}
**15**	38828140	rs8043085	T	G	0.4493	1.05	2.06×10^{-14}
**15**	40615872	rs12907887	C	G	0.229	1.08	1.74×10^{-20}
**15**	52587740	rs149336329	G	T	0.9494	1.11	1.70×10^{-9}
**15**	62394264	rs8037894	G	C	0.5856	1.08	7.30×10^{-33}
**15**	68080886	rs4776970	A	T	0.2214	1.04	3.41×10^{-8}
**15**	75742095	rs8038760	A	C	0.6081	1.05	1.81×10^{-11}
**15**	77776562	rs952472	C	A	0.3949	1.07	1.62×10^{-26}
**15**	90428894	rs10852123	A	C	0.201	1.06	8.38×10^{-13}
**15**	91522253	rs8026714	A	G	0.486	1.07	1.06×10^{-22}
**15**	93825384	rs61021634	A	G	0.4379	1.05	1.41×10^{-11}
**15**	99366409	rs79826452	A	G	0.8904	1.07	3.16×10^{-8}
**16**	3647098	rs2240885	A	G	0.4027	1.04	2.79×10^{-9}
**16**	20323168	rs117267808	A	G	0.0782	1.11	4.85×10^{-17}
**16**	53800954	rs1421085	C	T	0.1668	1.14	1.55×10^{-48}
**16**	72022534	rs12600132	T	C	0.4316	1.04	5.95×10^{-9}
**16**	73100308	rs6416749	C	T	0.3746	1.05	3.40×10^{-12}
**16**	81534790	rs2925979	T	C	0.3635	1.04	1.51×10^{-9}
**17**	6953781	rs186568031	T	C	0.0942	1.12	8.99×10^{-24}
**17**	29642430	rs7502556	T	C	0.5346	1.05	3.79×10^{-11}
**17**	36101586	rs8064454	A	C	0.3049	1.13	6.47×10^{-61}
**17**	65641651	rs2706710	T	C	0.0813	1.07	1.67×10^{-8}
**17**	73187031	rs35559984	CA	C	0.6519	1.05	7.88×10^{-9}
**18**	7076836	rs9948462	T	C	0.7048	1.05	8.70×10^{-10}
**18**	57852587	rs476828	C	T	0.243	1.09	4.81×10^{-27}
**18**	60845884	rs12454712	T	C	0.5122	1.06	1.40×10^{-15}
**19**	7293119	rs8101064	T	C	0.1277	1.07	3.53×10^{-8}

**19**	7986638	rs475002	G	C	0.5184	1.04	9.80×10^{-10}
**19**	12509536	rs4804181	A	C	0.5139	1.04	1.54×10^{-8}
**19**	21529576	rs145389767	G	A	0.9794	1.22	2.42×10^{-13}
**19**	22100706	rs142395395	A	G	0.9691	1.24	6.95×10^{-23}
**19**	33894846	rs10422861	C	T	0.5358	1.06	2.29×10^{-16}
**19**	46157928	rs113036890	CAAAAAAAA	C	0.7313	1.1	1.03×10^{-32}
**20**	22430241	rs73085586	G	A	0.6437	1.04	1.66×10^{-9}
**20**	42994812	rs12625671	C	T	0.442	1.07	2.25×10^{-21}
**20**	48830772	rs13040225	A	T	0.5132	1.05	1.64×10^{-14}
**20**	50155386	rs6021276	T	C	0.4101	1.04	6.66×10^{-10}
**20**	57477177	rs11477757	TC	TCC	0.6687	1.06	1.27×10^{-8}
**22**	29380119	rs147413364	T	TA	0.3571	1.04	3.38×10^{-8}
**22**	46313618	rs28637892	T	G	0.2153	1.05	3.66×10^{-9}
**22**	50356302	rs28691713	C	T	0.5552	1.07	1.79×10^{-17}

[a] Chr is chromosome.
[b] Position is in base pairs (bp) on Genome Reference Consortium Human Build 37.
[c] A1 and a2 are the alleles from AGEN 2020, with the effect allele given first.
[d] A1f is the frequency of a1 in AGEN 2020 study population.
[e] OR is reported per copy of the effect allele, and is also reported with the 95% confidence interval (CI) of the OR.

Table S4. Summary statistics for SNPs included in the construction of the DIAGRAM 2018[8] PS.

chr[a]	position[b]	SNP	a1[c]	a2	a1f[d]	OR[e]	*p*-value
**1**	40035928	rs3768321	T	G	0.2	1.09	2.6×10^{-26}
**1**	117532790	rs1127215	C	T	0.584	1.05	1.6×10^{-13}
**1**	120526982	rs1493694	T	C	0.109	1.09	2.7×10^{-16}
**1**	150786038	rs10305745	A	G	0.015	1.28	4.4×10^{-6}
**1**	177889025	rs539515	C	A	0.198	1.05	1.6×10^{-10}
**1**	205114873	rs12048743	G	C	0.442	1.04	3.5×10^{-9}
**1**	206593900	rs9430095	C	G	0.494	1.04	1.9×10^{-8}
**1**	214159256	rs340874	C	T	0.556	1.07	1.6×10^{-22}
**1**	219748818	rs2820446	C	G	0.706	1.06	3.3×10^{-16}
**1**	229672955	rs348330	G	A	0.361	1.05	2.7×10^{-14}
**1**	235690800	rs291367	G	A	0.632	1.04	4.7×10^{-10}
**2**	422144	rs62107261	T	C	0.954	1.12	3.8×10^{-12}
**2**	653575	rs35913461	C	T	0.829	1.06	1.6×10^{-11}
**2**	16574669	rs11680058	A	G	0.863	1.06	1.4×10^{-8}
**2**	25643221	rs17802463	G	T	0.731	1.04	2.9×10^{-8}
**2**	27730940	rs1260326	C	T	0.607	1.07	6.5×10^{-25}
**2**	43207872	rs28525376	G	T	0.422	1.03	2.7×10^{-6}
**2**	43430440	rs6708643	A	G	0.501	1.04	3.9×10^{-8}
**2**	58981064	rs10193538	T	G	0.61	1.04	8.9×10^{-9}
**2**	59307725	rs6545714	G	A	0.392	1.04	8.9×10^{-9}
**2**	60583665	rs243024	A	G	0.46	1.06	2.5×10^{-20}
**2**	65287896	rs2249105	A	G	0.634	1.1	2.2×10^{-14}
**2**	65355270	rs2052261	G	A	0.304	1.07	2.5×10^{-6}
**2**	65655012	rs2028150	C	G	0.598	1.05	2.3×10^{-12}
**2**	121318166	rs11688931	C	G	0.849	1.04	8.2×10^{-6}
**2**	121347612	rs11688682	G	C	0.728	1.05	4.2×10^{-9}
**2**	147861633	rs35999103	T	C	0.155	1.05	9.7×10^{-9}
**2**	158339550	rs13426680	A	G	0.937	1.09	6.7×10^{-10}
**2**	161135544	rs3772071	T	C	0.714	1.05	1.2×10^{-11}
**2**	165513091	rs10195252	T	C	0.586	1.07	6.0×10^{-25}
**2**	227101411	rs2972144	G	A	0.639	1.1	$2.1\times10^{-}46$
**3**	12336507	rs11709077	G	A	0.877	1.14	1.8×10^{-36}
**3**	12489342	rs17819328	G	T	0.425	1.06	4.8×10^{-16}
**3**	23510044	rs17013314	G	A	0.031	1.11	8.4×10^{-9}
**3**	46925539	rs11926707	C	T	0.626	1.27	2.1×10^{-8}
**3**	47242923	rs75423501	G	A	0.101	1.05	7.5×10^{-6}
**3**	49980596	rs4688760	T	C	0.684	1.04	3.5×10^{-10}
**3**	53127677	rs2581787	T	G	0.563	1.04	2.4×10^{-8}
**3**	63962339	rs3774723	G	A	0.844	1.07	1.6×10^{-13}
**3**	64701146	rs9860730	A	G	0.704	1.06	4.9×10^{-15}

**3**	72865183	rs13085136	C	T	0.928	1.08	1.5×10^{-8}
**3**	77671721	rs2272163	C	A	0.618	1.04	9.6×10^{-9}
**3**	123065778	rs11708067	A	G	0.772	1.09	5.2×10^{-32}
**3**	124926637	rs649961	T	C	0.465	1.04	9.9×10^{-10}
**3**	152086533	rs147579559	A	G	0.6	1.05	8.1×10^{-13}
**3**	152417881	rs74653713	C	A	0.957	1.1	1.2×10^{-8}
**3**	152433628	rs35497231	C	T	0.317	1.04	7.6×10^{-8}
**3**	168218841	rs7629630	A	T	0.857	1.05	2.5×10^{-8}
**3**	170733076	rs9873618	G	A	0.71	1.07	4.8×10^{-21}
**3**	183738460	rs2872246	A	C	0.454	1.04	1.5×10^{-8}
**3**	185503456	rs6780171	A	T	0.314	1.14	9.0×10^{-56}
**3**	185541213	rs11717959	G	T	0.621	1.04	3.0×10^{-6}
**3**	185829891	rs1516728	A	T	0.759	1.03	6.3×10^{-6}
**3**	186665645	rs3887925	T	C	0.547	1.07	3.1×10^{-22}
**3**	186675277	rs7645517	A	G	0.058	1.08	2.5×10^{-8}
**4**	744972	rs1531583	T	G	0.046	1.13	3.5×10^{-14}
**4**	1010077	rs35654957	C	T	0.367	1.03	4.2×10^{-7}
**4**	1784403	rs56337234	C	T	0.503	1.06	8.6×10^{-18}
**4**	3241845	rs362307	T	C	0.077	1.08	1.1×10^{-9}
**4**	6302519	rs1801212	A	G	0.709	1.05	4.5×10^{-6}
**4**	6306763	rs10937721	C	G	0.588	1.06	1.5×10^{-8}
**4**	17792869	rs12640250	C	A	0.715	1.04	3.7×10^{-8}
**4**	45186139	rs10938398	A	G	0.429	1.05	3.6×10^{-12}
**4**	52818664	rs2102278	G	A	0.319	1.04	3.7×10^{-8}
**4**	89740894	rs1903002	G	C	0.501	1.04	2.7×10^{-8}
**4**	104140848	rs1580278	C	A	0.473	1.04	2.2×10^{-10}
**4**	137083193	rs1296328	A	C	0.446	1.04	3.5×10^{-8}
**4**	153513369	rs7669833	T	A	0.705	1.06	1.2×10^{-14}
**4**	157652753	rs28819812	C	A	0.677	1.04	2.2×10^{-8}
**4**	185717759	rs58730668	T	C	0.858	1.07	1.3×10^{-13}
**5**	14768092	rs6885132	C	G	0.904	1.07	1.7×10^{-8}
**5**	44682589	rs6884702	G	A	0.393	1.04	1.5×10^{-10}
**5**	51791225	rs17261179	T	C	0.517	1.04	1.3×10^{-8}
**5**	52100489	rs3811978	G	A	0.167	1.06	7.7×10^{-11}
**5**	52774510	rs62370480	A	G	0.22	1.04	2.0×10^{-6}
**5**	53271420	rs702634	A	G	0.69	1.05	7.7×10^{-14}
**5**	53412620	rs279744	C	A	0.691	1.04	3.1×10^{-8}
**5**	55808475	rs465002	T	C	0.742	1.11	$6.1\times10^{-3}8$
**5**	55848669	rs2431115	A	G	0.402	1.04	3.9×10^{-10}
**5**	55861595	rs9687832	A	G	0.198	1.08	1.7×10^{-20}
**5**	56196604	rs96844	G	A	0.262	1.04	5.4×10^{-8}
**5**	67714246	rs4976033	G	A	0.411	1.05	1.0×10^{-9}
**5**	75003678	rs2307111	T	C	0.605	1.05	2.1×10^{-16}

**5**	76424949	rs4457053	G	A	0.304	1.06	8.4×10^{-18}
**5**	78430607	rs1316776	C	A	0.648	1.05	2.6×10^{-12}
**5**	86577352	rs7719891	G	A	0.259	1.04	2.4×10^{-8}
**5**	133414622	rs244665	A	G	0.703	1.03	9.9×10^{-6}
**5**	133864599	rs329122	A	G	0.429	1.04	3.6×10^{-9}
**5**	157928196	rs3934712	C	T	0.206	1.05	3.2×10^{-8}
**6**	7035734	rs112498319	C	A	0.409	1.03	4.2×10^{-7}
**6**	7255650	rs9505097	C	T	0.799	1.05	8.6×10^{-10}
**6**	20679709	rs7756992	G	A	0.274	1.15	2.4×10^{-88}
**6**	32573415	rs601945	G	A	0.178	1.06	4.7×10^{-8}
**6**	34524698	rs2233632	T	C	0.688	1.04	5.3×10^{-7}
**6**	40409243	rs34298980	T	C	0.497	1.04	9.3×10^{-10}
**6**	43760327	rs11967262	G	C	0.486	1.04	8.8×10^{-10}
**6**	43814190	rs6458354	C	T	0.289	1.05	2.1×10^{-12}
**6**	50788778	rs3798519	C	A	0.184	1.06	2.6×10^{-12}
**6**	51180765	rs2465043	G	A	0.644	1.03	2.9×10^{-6}
**6**	107431688	rs4946812	G	A	0.674	1.04	8.2×10^{-9}
**6**	126792095	rs11759026	G	A	0.232	1.07	2.4×10^{-18}
**6**	127416930	rs2800733	A	G	0.717	1.05	6.0×10^{-11}
**6**	137300960	rs9494624	A	G	0.29	1.04	6.1×10^{-9}
**6**	140249466	rs616279	A	G	0.738	1.04	6.7×10^{-7}
**6**	160770312	rs474513	A	G	0.517	1.04	8.1×10^{-10}
**6**	164133001	rs4709746	C	T	0.868	1.06	5.8×10^{-9}
**7**	14898282	rs17168486	T	C	0.181	1.07	2.3×10^{-17}
**7**	15063569	rs10228066	T	C	0.537	1.07	1.1×10^{-28}
**7**	15206239	rs2908334	T	C	0.631	1.03	5.9×10^{-6}
**7**	23434606	rs78840640	G	C	0.022	1.11	2.8×10^{-6}
**7**	23512896	rs4279506	G	C	0.61	1.06	4.8×10^{-8}
**7**	28198677	rs1708302	C	T	0.512	1.1	1.1×10^{-48}
**7**	30728452	rs917195	C	T	0.77	1.05	4.2×10^{-11}
**7**	44255643	rs878521	A	G	0.245	1.06	1.9×10^{-13}
**7**	102486254	rs11496066	T	C	0.818	1.08	1.1×10^{-8}
**7**	102987583	rs62482405	G	T	0.082	1.05	6.9×10^{-6}
**7**	103444978	rs39328	T	C	0.433	1.04	3.7×10^{-8}
**7**	117495667	rs6976111	A	C	0.313	1.04	1.2×10^{-8}
**7**	130027037	rs2268382	C	A	0.327	1.03	7.4×10^{-6}
**7**	130457914	rs1562396	G	A	0.319	1.06	9.9×10^{-18}
**7**	150537635	rs62492368	A	G	0.308	1.05	1.1×10^{-10}
**7**	156930550	rs6459733	G	C	0.673	1.06	2.4×10^{-17}
**8**	9974824	rs17689007	G	A	0.533	1.04	2.5×10^{-9}
**8**	10808687	rs57327348	A	T	0.782	1.04	4.5×10^{-8}
**8**	30863938	rs10954772	T	C	0.314	1.04	1.8×10^{-9}
**8**	41509915	rs4736819	T	C	0.554	1.04	5.4×10^{-7}

**8**	110123183	rs12680028	C	G	0.534	1.04	2.5×10^{-8}
**8**	118185025	rs3802177	G	A	0.685	1.11	1.1×10^{-55}
**8**	128711742	rs17772814	G	A	0.915	1.08	5.4×10^{-10}
**8**	129568078	rs1561927	C	T	0.269	1.04	1.5×10^{-9}
**8**	145507304	rs4977213	C	T	0.375	1.05	9.1×10^{-14}
**8**	145879883	rs12719778	T	C	0.538	1.04	5.0×10^{-9}
**9**	3965689	rs510807	A	C	0.491	1.03	1.4×10^{-6}
**9**	4291928	rs10974438	C	A	0.357	1.05	1.5×10^{-14}
**9**	19067833	rs7022807	G	A	0.401	1.04	2.7×10^{-10}
**9**	20241069	rs7867635	C	T	0.412	1.04	4.0×10^{-8}
**9**	20662703	rs7847880	C	T	0.843	1.04	2.1×10^{-6}
**9**	22133773	rs76011118	A	G	0.034	1.11	1.4×10^{-7}
**9**	22134068	rs10811660	G	A	0.828	1.27	1.4×10^{-115}
**9**	22134172	rs10757283	T	C	0.43	1.11	1.7×10^{-41}
**9**	22157908	rs1333052	A	C	0.66	1.03	6.3×10^{-7}
**9**	28410683	rs1412234	C	T	0.323	1.04	1.9×10^{-10}
**9**	34074476	rs12001437	C	T	0.372	1.04	2.8×10^{-10}
**9**	81359113	rs11137820	C	G	0.575	1.04	2.9×10^{-8}
**9**	81905590	rs17791513	A	G	0.932	1.1	3.1×10^{-14}
**9**	84308948	rs2796441	G	A	0.592	1.07	4.4×10^{-24}
**9**	97001682	rs55653563	A	C	0.732	1.04	2.2×10^{-9}
**9**	136149229	rs505922	C	T	0.332	1.05	3.9×10^{-12}
**9**	139235606	rs78403475	G	C	0.896	1.06	1.2×10^{-6}
**9**	139241030	rs28505901	G	A	0.752	1.09	6.7×10^{-26}
**9**	139507212	rs11793035	C	T	0.331	1.04	2.2×10^{-7}
**10**	12307894	rs11257655	T	C	0.218	1.09	1.5×10^{-32}
**10**	71321279	rs177045	G	A	0.316	1.07	6.6×10^{-18}
**10**	71321658	rs61850200	C	G	0.277	1.04	7.3×10^{-6}
**10**	71466578	rs2642588	G	T	0.702	1.05	2.2×10^{-14}
**10**	80952826	rs703972	G	C	0.533	1.07	1.7×10^{-29}
**10**	81096589	rs1317617	G	A	0.798	1.04	1.8×10^{-6}
**10**	89769340	rs11202627	T	C	0.152	1.06	4.7×10^{-8}
**10**	93924663	rs7078559	T	C	0.578	1.03	4.1×10^{-7}
**10**	94462427	rs10882101	T	C	0.587	1.06	1.4×10^{-8}
**10**	94479107	rs1112718	A	G	0.598	1.06	5.0×10^{-7}
**10**	114703136	rs7918400	T	C	0.476	1.06	2.0×10^{-15}
**10**	114757956	rs78025551	C	G	0.851	1.05	1.6×10^{-7}
**10**	114758349	rs7903146	T	C	0.295	1.37	5.8×10^{-447}
**10**	114871594	rs34855922	A	G	0.716	1.05	5.5×10^{-12}
**10**	122915345	rs72631105	A	G	0.19	1.06	3.7×10^{-9}
**10**	124193181	rs2280141	T	G	0.516	1.05	1.4×10^{-13}
**11**	1704596	rs12802972	A	G	0.428	1.03	1.5×10^{-6}
**11**	2118860	rs11042596	G	T	0.665	1.04	2.0×10^{-8}

**11**	2197286	rs4929965	A	G	0.383	1.07	4.0×10^{-26}
**11**	2372356	rs4930091	C	T	0.759	1.04	3.7×10^{-6}
**11**	2579163	rs2283164	A	G	0.947	1.08	1.2×10^{-7}
**11**	2691500	rs231361	A	G	0.256	1.08	5.0×10^{-25}
**11**	2755548	rs2283220	A	G	0.69	1.05	1.4×10^{-9}
**11**	2850828	rs234853	G	A	0.248	1.08	6.8×10^{-16}
**11**	2857194	rs2237895	C	A	0.426	1.12	6.0×10^{-52}
**11**	2858546	rs2237897	C	T	0.954	1.23	8.4×10^{-32}
**11**	2908754	rs445084	G	A	0.361	1.03	1.7×10^{-6}
**11**	17408404	rs5213	C	T	0.362	1.07	3.5×10^{-27}
**11**	28534898	rs4923543	A	G	0.332	1.04	4.5×10^{-8}
**11**	32927778	rs145678014	G	T	0.957	1.11	2.0×10^{-10}
**11**	34642668	rs286925	A	G	0.182	1.04	5.0×10^{-6}
**11**	34982148	rs2767036	C	A	0.291	1.04	3.3×10^{-8}
**11**	43877934	rs1061810	A	C	0.288	1.05	6.0×10^{-13}
**11**	45912013	rs7115753	A	G	0.449	1.04	3.8×10^{-9}
**11**	47529947	rs7124681	A	C	0.41	1.04	5.1×10^{-9}
**11**	65294799	rs1783541	T	C	0.204	1.06	2.0×10^{-14}
**11**	68997225	rs61881115	G	A	0.838	1.05	4.1×10^{-7}
**11**	72460398	rs77464186	A	C	0.836	1.11	4.7×10^{-33}
**11**	92708710	rs10830963	G	C	0.277	1.1	4.8×10^{-43}
**11**	128042575	rs10893829	T	C	0.853	1.06	1.3×10^{-10}
**11**	128234144	rs10750397	A	G	0.282	1.05	8.3×10^{-13}
**11**	128398938	rs67232546	T	C	0.207	1.06	1.3×10^{-11}
**12**	4031104	rs10848958	C	T	0.804	1.04	1.5×10^{-7}
**12**	4300172	rs11063028	C	T	0.18	1.06	8.5×10^{-11}
**12**	4376089	rs4238013	C	T	0.209	1.06	3.2×10^{-11}
**12**	4399050	rs3217860	G	A	0.258	1.05	3.9×10^{-9}
**12**	26453283	rs718314	G	A	0.253	1.05	8.4×10^{-11}
**12**	27965150	rs10842994	C	T	0.805	1.08	4.1×10^{-20}
**12**	66221060	rs2258238	T	A	0.104	1.1	4.5×10^{-21}
**12**	66358347	rs1042725	T	C	0.49	1.05	1.8×10^{-13}
**12**	71522953	rs1796330	G	C	0.571	1.05	2.2×10^{-14}
**12**	97848775	rs77864822	A	G	0.932	1.08	1.1×10^{-8}
**12**	108629780	rs1426371	G	A	0.739	1.05	8.2×10^{-12}
**12**	118412373	rs34965774	A	G	0.144	1.06	2.0×10^{-9}
**12**	118489636	rs12578639	A	T	0.828	1.04	2.2×10^{-6}
**12**	121297815	rs11065299	A	G	0.075	1.06	5.8×10^{-7}
**12**	121432117	rs56348580	G	C	0.689	1.05	2.3×10^{-13}
**12**	123450765	rs4148856	C	G	0.781	1.05	1.7×10^{-10}
**12**	124468572	rs7978610	G	C	0.666	1.27	2.0×10^{-8}
**12**	124509177	rs825452	A	G	0.603	1.04	2.4×10^{-6}
**12**	133069698	rs12811407	A	G	0.331	1.05	1.7×10^{-12}

**13**	26776999	rs34584161	A	G	0.76	1.05	2.2×10^{-10}
**13**	31042452	rs11842871	G	T	0.735	1.04	1.2×10^{-8}
**13**	33554302	rs576674	G	A	0.169	1.05	8.3×10^{-10}
**13**	51096095	rs963740	A	T	0.713	1.04	2.1×10^{-8}
**13**	58366634	rs9537803	C	T	0.277	1.04	4.6×10^{-8}
**13**	58965435	rs9569864	C	T	0.825	1.05	8.7×10^{-8}
**13**	59077406	rs9563615	A	T	0.71	1.05	6.4×10^{-11}
**13**	80717156	rs1359790	G	A	0.72	1.09	2.4×10^{-31}
**13**	109947213	rs7987740	T	C	0.609	1.04	4.0×10^{-8}
**13**	110431626	rs4771648	G	A	0.669	1.04	8.9×10^{-8}
**14**	23288935	rs17122772	G	C	0.228	1.04	1.6×10^{-8}
**14**	33302882	rs17522122	T	G	0.474	1.04	3.2×10^{-9}
**14**	38848419	rs8017808	G	T	0.743	1.04	2.1×10^{-8}
**14**	79932041	rs17836088	C	G	0.217	1.06	6.7×10^{-14}
**14**	103894071	rs62007683	G	T	0.653	1.04	3.1×10^{-8}
**15**	38834033	rs8032939	C	T	0.246	1.06	3.5×10^{-14}
**15**	41809205	rs11070332	A	G	0.358	1.05	1.1×10^{-13}
**15**	53091553	rs2456530	T	C	0.127	1.06	5.4×10^{-9}
**15**	57456802	rs117483894	G	A	0.037	1.1	3.9×10^{-8}
**15**	62394264	rs8037894	G	C	0.566	1.05	2.6×10^{-13}
**15**	63871292	rs7178762	C	T	0.46	1.04	5.4×10^{-10}
**15**	68080886	rs4776970	A	T	0.641	1.04	5.0×10^{-9}
**15**	75932129	rs13737	G	T	0.759	1.05	5.6×10^{-10}
**15**	77818128	rs1005752	A	C	0.715	1.08	2.5×10^{-29}
**15**	90423293	rs4932265	T	C	0.267	1.07	4.2×10^{-20}
**15**	91511260	rs12910825	G	A	0.361	1.05	1.6×10^{-15}
**16**	295795	rs6600191	T	C	0.825	1.06	9.3×10^{-13}
**16**	3583173	rs3751837	T	C	0.22	1.04	1.4×10^{-8}
**16**	28915217	rs8046545	G	A	0.359	1.04	1.9×10^{-8}
**16**	53800954	rs1421085	C	T	0.415	1.13	3.1×10^{-84}
**16**	69651866	rs862320	C	T	0.578	1.04	3.9×10^{-11}
**16**	75234872	rs72802342	C	A	0.923	1.17	4.0×10^{-32}
**16**	75516534	rs3115960	G	C	0.37	1.03	2.8×10^{-6}
**16**	81534790	rs2925979	T	C	0.3	1.05	1.4×10^{-14}
**17**	3828086	rs1043246	G	C	0.157	1.05	7.9×10^{-7}
**17**	3860356	rs3826482	A	T	0.576	1.03	2.1×10^{-7}
**17**	4045440	rs1377807	C	G	0.312	1.05	4.2×10^{-13}
**17**	7549681	rs1641523	C	T	0.428	1.05	1.2×10^{-10}
**17**	9785187	rs7222481	C	G	0.324	1.04	1.4×10^{-8}
**17**	17661802	rs4925109	A	G	0.316	1.05	2.8×10^{-12}
**17**	36046451	rs10962	C	G	0.226	1.05	9.9×10^{-10}
**17**	36099952	rs10908278	T	A	0.481	1.08	6.4×10^{-3}6
**17**	40731411	rs34855406	C	G	0.277	1.05	2.3×10^{-12}

**17**	47060322	rs35895680	C	A	0.678	1.06	2.5×10^{-15}
**17**	61965043	rs2727301	T	C	0.754	1.04	1.3×10^{-6}
**17**	62203304	rs60276348	T	C	0.14	1.05	2.6×10^{-8}
**17**	65892507	rs61676547	C	G	0.192	1.06	2.9×10^{-11}
**18**	7070642	rs7240767	C	T	0.376	1.04	1.6×10^{-8}
**18**	53452144	rs28719468	C	T	0.159	1.04	1.9×10^{-6}
**18**	54675384	rs17684074	G	C	0.74	1.04	2.9×10^{-8}
**18**	56876228	rs9957145	G	A	0.829	1.05	8.1×10^{-9}
**18**	57848369	rs523288	T	A	0.238	1.05	7.6×10^{-13}
**18**	58056566	rs74452128	C	A	0.976	1.15	1.0×10^{-9}
**18**	60668270	rs10469140	G	A	0.485	1.03	6.6×10^{-6}
**18**	60845884	rs12454712	T	C	0.614	1.05	4.6×10^{-13}
**19**	4948862	rs7249758	A	G	0.204	1.05	3.4×10^{-9}
**19**	7240848	rs75253922	C	T	0.191	1.05	2.7×10^{-8}
**19**	7970635	rs4804833	A	G	0.39	1.05	7.7×10^{-13}
**19**	19388500	rs8107974	T	A	0.077	1.1	3.3×10^{-15}
**19**	33890838	rs10406327	C	G	0.523	1.04	3.8×10^{-8}
**19**	44938870		A	G	0.001	1.61	8.3×10^{-6}
**19**	45411941	rs429358	T	C	0.846	1.08	2.6×10^{-18}
**19**	46157019	rs10406431	A	G	0.563	1.05	9.6×10^{-14}
**19**	46178661	rs2238689	C	T	0.418	1.04	5.4×10^{-9}
**19**	47569003	rs3810291	A	G	0.673	1.05	8.9×10^{-12}
**20**	21466795	rs13041756	C	T	0.107	1.06	1.4×10^{-8}
**20**	32596704	rs2268078	A	G	0.657	1.04	2.3×10^{-10}
**20**	43001721	rs4810426	T	C	0.106	1.09	3.1×10^{-17}
**20**	43042364	rs1800961	T	C	0.035	1.18	2.3×10^{-22}
**20**	43233649	rs11696357	A	G	0.934	1.06	9.9×10^{-6}
**20**	45598564	rs6063048	G	A	0.725	1.05	2.2×10^{-11}
**20**	48832135	rs11699802	C	T	0.536	1.04	1.8×10^{-11}
**20**	51223594	rs34454109	A	T	0.771	1.04	7.1×10^{-9}
**20**	57394628	rs6070625	G	C	0.517	1.05	5.3×10^{-14}
**20**	62450664	rs6011155	T	C	0.63	1.04	6.3×10^{-6}
**20**	62693175	rs59944054	A	G	0.238	1.06	1.5×10^{-8}
**22**	30609554	rs6518681	G	A	0.914	1.09	1.1×10^{-12}
**22**	32348841	rs117001013	C	T	0.912	1.07	1.7×10^{-8}
**22**	41489920	rs5758223	A	G	0.717	1.04	3.8×10^{-8}
**22**	44324730	rs738408	T	C	0.226	1.05	1.4×10^{-10}
**22**	50356850	rs1801645	C	T	0.275	1.04	1.5×10^{-8}

[a] Chr is chromosome.
[b] Position is in base pairs (bp) on Genome Reference Consortium Human Build 37.
[c] A1 and a2 are the alleles from AGEN 2020, with the effect allele given first.
[d] A1f is the frequency of a1 in the DIAGRAM 2018 study population.
[e] OR is reported per copy of the effect allele.

Table S5. SNPs included in the PSs derived from various ancestry-specific GWAS meta-analyses in DIAMANTE 2022[9] (including the population-specific weight PS).

chr[a]	position[b]	SNP	a1/a2[c]	OR_comp[d]	OR_afr	OR_eas	OR_eur	OR_his	OR_sas	freq_pop[e]	OR_pop[f]	*p*-value
**1**	20729451	rs10916784	G/C	1.03	1.03	1.03	1.03	1.02	1.06	0.49	1.05	3.1×10^{-1}
**1**	39870793	rs3768301	T/C	1.07	1.08	1.06	1.08	1.04	1.05	0.30	1.06	2.6×10^{-1}
**1**	46358862	rs34444543	G/A	1.04	1.02	1.06	1.03	1.01	1.02	0.61	1.05	3.0×10^{-1}
**1**	64114429	rs11576729	G/T	1.05	1.08	1.06	1.04	1.09	1.03	0.50	1.04	3.6×10^{-1}
**1**	117532790	rs1127215	C/T	1.04	1.02	1.02	1.05	1.06	1.05	0.21	0.96	4.7×10^{-1}
**1**	120455586	rs835576	C/T	1.07	1.03	1.09	1.08	1.09	1.03	0.10	0.94	4.6×10^{-1}
**1**	177889025	rs539515	C/A	1.06	1.04	1.06	1.06	1.06	1.03	0.20	1.03	5.4×10^{-1}
**1**	200416099	rs10919928	A/G	1.03	0.77	1.04	1.15	1.14	0.98	0.13	1.13	9.1×10^{-2}
**1**	204539291	rs6689629	A/G	1.04	1.05	1.04	1.03	1.03	1.04	0.98	0.93	6.7×10^{-1}
**1**	205107793	rs12039805	A/G	1.03	1.03	1.02	1.04	1.06	1.01	0.39	1.03	6.0×10^{-1}
**1**	206600992	rs9429893	A/G	1.03	1.08	1.02	1.04	1.02	1.03	0.48	1.03	5.5×10^{-1}
**1**	214159256	rs340874	C/T	1.05	1.01		1.06	1.04	1.04	0.35	1.04	4.2×10^{-1}
**1**	219748818	rs2820446	C/G	1.05	1.06	1.03	1.06	1.02	1.03	0.38	1.06	1.8×10^{-1}
**1**	229672955	rs348330	G/A	1.05	1.02	1.06	1.05	1.03	1.02	0.68	1.01	8.0×10^{-1}
**2**	422144	rs62107261	T/C	1.11	1.11		1.11	1.12	0.97	0.99	1.35	1.9×10^{-1}
**2**	653874	rs10188334	C/T	1.06	1.11		1.05	1.07	1.10	0.85	1.07	3.2×10^{-1}
**2**	25533568	rs55928417	G/T	1.03	0.99	1.03	1.04	1.04	1.05	0.84	1.06	4.1×10^{-1}
**2**	27730940	rs1260326	C/T	1.06	1.07	1.06	1.07	1.03	1.08	0.90	1.07	5.3×10^{-1}
**2**	45192080	rs12712928	C/G	1.01	0.95	1.07	0.97	0.95	1.00	0.35	0.91	8.8×10^{-2}
**2**	60586707	rs243018	G/C	1.06	1.09	1.06	1.06	1.06	1.05	0.74	1.02	6.8×10^{-1}
**2**	65284231	rs2540949	A/T	1.03	1.03	1.02	1.04	1.03	1.02	0.79	1.10	1.3×10^{-1}
**2**	65666674	rs6752053	T/C	1.05	1.04	1.05	1.05	1.05	1.05	0.73	0.93	1.9×10^{-1}
**2**	121347612	rs11688682	G/C	1.06	1.02	1.01	1.06	1.05	1.06	0.99	1.47	3.0×10^{-1}
**2**	121440218	rs10864859	T/G	1.06	1.03	1.04	1.06	1.05	1.13	0.65	1.02	7.1×10^{-1}
**2**	158390468	rs7594480	T/C	1.08	1.07	0.99	1.08	1.10	1.09	0.84	0.98	7.7×10^{-1}
**2**	161144055	rs1020731	A/G	1.03	1.04	1.02	1.04	1.06	0.98	0.94	1.14	1.6×10^{-1}
**2**	163649480	rs12614955	T/C	1.03	1.05	1.03	1.03	1.01	1.04	0.60	0.96	3.5×10^{-1}

**2**	165508389	rs10184004	C/T	1.07	1.11	1.05	1.06	1.08	1.09	0.97	1.14	$3.8{\times}10^{-1}$
**2**	213818731	rs16849467	T/C	1.04	1.06	1.04	1.03	1.02	1.05	0.97	0.98	$8.5{\times}10^{-1}$
**2**	227100490	rs2943648	G/A	1.09	1.08	1.05	1.10	1.07	1.08	0.99	1.08	$7.3{\times}10^{-1}$
**3**	12329783	rs17036160	C/T	1.12	1.09	1.12	1.12	1.12	1.12	0.91	1.18	$4.6{\times}10^{-2}$*
**3**	12490951	rs4684855	T/C	1.04	1.11	1.03	1.05	1.03	1.01	0.40	1.07	$1.5{\times}10^{-1}$
**3**	23457080	rs13094957	T/C	1.07	1.02	1.11	1.07	1.05	1.08	0.95	0.99	$8.2{\times}10^{-1}$
**3**	23632174	rs76435632	G/C	1.10	1.21	1.11	1.11	1.08	1.08	0.12	1.07	$2.9{\times}10^{-1}$
**3**	50174197	rs2624847	G/T	1.03	0.97	1.04	1.04	1.04	1.00	0.13	1.07	$3.6{\times}10^{-1}$
**3**	58338809	rs12629058	T/C	1.04	1.13	1.04	1.03	1.05	1.06	0.16	1.04	$6.6{\times}10^{-1}$
**3**	63897215	rs2292662	C/T	1.06	1.02	1.09	1.07	1.01	1.01	0.98	1.01	$9.0{\times}10^{-1}$
**3**	64703394	rs66815886	G/T	1.04	1.05	1.01	1.05	1.03	1.03	0.96	0.84	$2.7{\times}10^{-1}$
**3**	114960798	rs1459513	C/A	1.05	0.98	1.06	1.06	1.06	1.01	0.16	1.00	$6.3{\times}10^{-1}$
**3**	123065778	rs11708067	A/G	1.10	1.13	1.18	1.09	1.13	1.11	0.54	1.03	$6.5{\times}10^{-1}$
**3**	124921457	rs9873519	T/C	1.04	1.05	1.04	1.04	1.01	1.05	0.29	1.01	$8.4{\times}10^{-1}$
**3**	151998053	rs1426385	A/G	1.04	1.05	1.02	1.04	1.02	1.05	0.42	1.03	$5.0{\times}10^{-1}$
**3**	152399693	rs10935897	A/G	1.03	1.04	1.03	1.04	1.06	1.01	0.18	0.93	$2.2{\times}10^{-1}$
**3**	152530027	rs75417759	C/T	1.07	1.07	0.96	1.11	1.11	1.06	0.98	1.07	$7.1{\times}10^{-1}$
**3**	170724883	rs8192675	T/C	1.06	0.97	1.06	1.07	1.01	1.05	0.76	1.08	$1.7{\times}10^{-1}$
**3**	185510613	rs7633675	G/T	1.12	1.13	1.14	1.12	1.16	1.12	0.18	1.07	$2.8{\times}10^{-1}$
**3**	186665645	rs3887925	T/C	1.05	1.03	1.04	1.06	1.05	1.05	0.14	1.09	$2.0{\times}10^{-1}$
**3**	186676455	rs9799068	A/C	1.04	1.03	1.03	1.06	1.03	1.03	0.15	1.01	$6.6{\times}10^{-1}$
**3**	195825077	rs74289356	T/C	1.05	1.07	1.06	1.04	1.06	1.01	0.41	0.96	$5.4{\times}10^{-1}$
**4**	1240299	rs730831	T/G	1.09	1.09	1.11	1.08	1.11	1.04	0.53	1.02	$7.6{\times}10^{-1}$
**4**	1784605	rs6831006	G/C	1.06	1.02	1.07	1.06	1.04	1.04	0.31	0.99	$7.4{\times}10^{-1}$
**4**	6293237	rs9998835	G/C	1.08	0.99	1.12	1.09	1.13	1.06	0.94	1.05	$6.5{\times}10^{-1}$
**4**	18047401	rs6855926	A/G	1.04	1.07	1.03	1.04	1.02	1.06	0.66	1.05	$4.1{\times}10^{-1}$
**4**	45175691	rs13130484	T/C	1.04	1.08	1.03	1.04	1.04	1.02	0.30	1.13	$1.8{\times}10^{-2}$*
**4**	71835822	rs7674402	A/G	1.06	1.04	1.07	1.06	1.06	1.06	0.15	1.03	$6.2{\times}10^{-1}$
**4**	83587562	rs10471048	G/C	1.03	1.05	1.02	1.04	1.01	1.04	0.23	0.96	$5.1{\times}10^{-1}$
**4**	102135363	rs2659518	A/G	1.04	1.06	1.03	1.05	1.07	1.03	0.40	1.07	$1.7{\times}10^{-1}$
**4**	103725894	rs223423	G/A	1.02	1.04	1.00	1.04	1.07	0.98	0.30	1.03	$5.6{\times}10^{-1}$

**4**	106048291	rs17035289	C/T	1.04	1.07	1.04	1.04	1.10	1.00	0.22	1.03	5.9×10^{-1}
**4**	153520475	rs6813195	C/T	1.06	1.08	1.07	1.06	1.08	1.06	0.53	1.10	5.3×10^{-2}
**4**	157725916	rs1425482	T/C	1.03	1.06	1.00	1.04	1.09	1.02	0.69	1.06	2.2×10^{-1}
**5**	14780521	rs30614	A/G	1.04	1.07		1.04	1.07	1.05	0.23	0.95	3.5×10^{-1}
**5**	44682589	rs6884702	G/A	1.03	0.99	1.02	1.04	1.06	1.02	0.52	1.00	8.4×10^{-1}
**5**	51791225	rs17261179	T/C	1.03	1.05	1.03	1.04	1.03	1.02	0.77	0.99	7.8×10^{-1}
**5**	53271420	rs702634	A/G	1.04	1.04	1.04	1.04	1.03	1.02	0.96	0.89	3.9×10^{-1}
**5**	53303595	rs6876198	C/T	1.04	1.07	1.02	1.04	1.07	1.04	0.24	1.00	8.9×10^{-1}
**5**	55810305	rs256904	T/A	1.08	1.11	1.09	1.09	1.07	1.05	0.77	1.17	8.7×10^{-3}*
**5**	55840633	rs42251	A/G	1.03	1.00	1.03	1.04	1.06	1.03	0.25	1.13	2.6×10^{-2}*
**5**	55860866	rs3936510	T/G	1.08	1.02	1.08	1.09	1.13	1.07	0.08	1.08	4.4×10^{-1}
**5**	67716793	rs57634870	G/T	1.04	1.04	1.04	1.04	1.04	1.05	0.23	0.99	7.3×10^{-1}
**5**	75003678	rs2307111	T/C	1.04	1.12	1.00	1.05	1.05	1.02	0.76	1.03	6.4×10^{-1}
**5**	76435004	rs7732130	G/A	1.06	1.15	1.08	1.06	1.04	1.03	0.35	1.11	4.1×10^{-2}*
**5**	78472599	rs10052346	G/T	1.04	1.08	1.02	1.04	1.07	1.02	0.47	0.96	4.5×10^{-1}
**5**	122704342	rs4267865	G/T	1.08	1.18	1.06	1.08	1.07	1.12	0.86	1.13	7.2×10^{-2}
**5**	133864599	rs329122	A/G	1.04	1.01	1.04	1.04	1.07	1.06	0.80	1.06	3.4×10^{-1}
**5**	176589585	rs244708	G/A	1.03	1.02	1.04	1.02	1.06	1.01	0.45	1.11	4.9×10^{-2}*
**6**	20680678	rs9348441	A/T	1.17	1.08	1.23	1.15	1.09	1.12	0.28	1.13	1.7×10^{-2}*
**6**	31139452	rs879882	C/T	1.04	0.97	1.05	1.04	1.07		0.60	1.02	6.2×10^{-1}
**6**	32373378	rs3806155	T/A	1.19	0.99	1.19	1.19	0.78		0.01	0.56	1.1×10^{-1}
**6**	32439077	rs7452864	C/T	1.04	1.14	1.00	1.05	1.02		0.97	1.10	6.6×10^{-1}
**6**	33524820	rs62405954	T/C	1.10	1.17	1.05	1.11	1.08		0.81	0.98	7.5×10^{-1}
**6**	34214670	rs4711389	A/G	1.08	1.30	1.13	1.05	1.07		0.51	1.06	2.9×10^{-1}
**6**	38992668	rs2281342	T/C	1.03	1.08	1.03	1.03	1.09	1.01	0.80	1.03	7.0×10^{-1}
**6**	39046644	rs742762	A/C	1.04	1.02	1.08	1.01	1.08	0.98	0.73	1.01	7.6×10^{-1}
**6**	39284184	rs3734618	G/A	1.04	1.05	1.07	1.03	1.05	1.05	0.15	1.17	2.6×10^{-2}*
**6**	40409243	rs34298980	T/C	1.03	1.01	1.02	1.04	1.07	1.02	0.41	1.06	2.8×10^{-1}
**6**	43758873	rs6905288	A/G	1.04	1.03	1.03	1.04	1.04	1.04	0.83	1.13	5.9×10^{-2}
**6**	43814190	rs6458354	C/T	1.04	1.03	1.04	1.05	1.05	0.99	0.06	1.01	8.2×10^{-1}
**6**	50788778	rs3798519	C/A	1.06	1.04	1.05	1.06	1.07	1.05	0.56	1.07	1.3×10^{-1}

**6**	107433400	rs1665901	A/T	1.04	1.00	1.04	1.04	1.02	1.03	0.66	0.98	6.7×10^{-1}
**6**	118011723	rs72951506	C/T	1.04	1.11	1.05	1.04	1.00	1.03	0.73	1.12	3.9×10^{-2}*
**6**	126792095	rs11759026	G/A	1.07	1.12	1.06	1.07	1.11	1.07	0.77	1.03	6.1×10^{-1}
**6**	127416930	rs2800733	A/G	1.05	1.04	1.07	1.05	1.05	1.04	0.84	1.03	6.5×10^{-1}
**6**	131954797	rs7739842	G/T	1.04	0.98	1.05	1.03	1.07	1.04	0.40	1.06	2.7×10^{-1}
**6**	137291281	rs6937795	A/C	1.04	0.98	1.04	1.05	1.01	1.02	0.63	1.02	6.6×10^{-1}
**6**	138855975	rs9376353	A/T	1.03	1.03	1.03	1.03	1.00	1.07	0.57	0.99	7.7×10^{-1}
**6**	143058692	rs6570526	G/C	1.03	1.03	1.03	1.03	1.04	1.02	0.49	0.98	7.1×10^{-1}
**6**	153438573	rs6932473	T/A	1.04	1.01	1.04	1.04	1.06	1.03	0.28	1.07	2.1×10^{-1}
**6**	160770360	rs539298	A/G	1.04	1.05	1.03	1.04	1.02	1.07	0.63	0.95	2.8×10^{-1}
**6**	164133001	rs4709746	C/T	1.05	1.02	1.05	1.06	1.04	0.99	0.50	1.09	6.1×10^{-2}
**7**	13887008	rs12154701	A/C	1.03	1.03	1.05	1.02	1.02	1.02	0.26	1.07	2.6×10^{-1}
**7**	14898282	rs17168486	T/C	1.06	1.01	1.07	1.07	1.05	1.05	0.83	1.05	5.1×10^{-1}
**7**	15062983	rs2215383	C/T	1.07	1.08	1.07	1.07	1.06	1.08	0.24	0.98	8.2×10^{-1}
**7**	28192280	rs849133	C/T	1.05	1.08		1.04	1.11	1.06	0.76	1.05	3.5×10^{-1}
**7**	28205303	rs552707	T/C	1.03	1.04		1.04	1.01	1.00	0.01	0.76	2.1×10^{-1}
**7**	30728452	rs917195	C/T	1.05	1.04	1.04	1.05	1.08	1.05	0.59	0.98	6.5×10^{-1}
**7**	44178829	rs882019	G/A	1.03	1.07	1.04	1.03	1.01	1.01	0.52	1.02	7.6×10^{-1}
**7**	44255643	rs878521	A/G	1.04	1.05	1.03	1.06	1.04	1.04	0.59	0.94	2.1×10^{-1}
**7**	50809085	rs13236710	G/A	1.05	1.05	1.06	1.05	1.09	0.97	0.93	1.02	8.8×10^{-1}
**7**	69055951	rs2533457	G/A	1.04	1.06	1.06	1.03	1.03	1.07	0.27	1.03	6.3×10^{-1}
**7**	89800241	rs6978118	A/T	1.03	1.05	1.07	1.02	1.04	1.01	0.39	1.07	1.9×10^{-1}
**7**	102481891	rs7781557	C/T	1.05	1.10	1.19	1.05	1.06	1.02	0.99	0.94	7.9×10^{-1}
**7**	130457914	rs1562396	G/A	1.04	1.01	1.03	1.06	1.04	1.00	0.33	1.06	2.6×10^{-1}
**7**	140631823	rs11983228	C/G	1.05	1.04	1.07	1.05	1.06	1.06	0.01	1.78	5.5×10^{-2}
**7**	150537635	rs62492368	A/G	1.03	1.00	1.03	1.04	1.03	1.01	0.53	0.97	6.0×10^{-1}
**7**	156794983	rs887609	A/G	1.03	1.01	1.06	1.02	1.10	0.99	0.25	1.01	7.8×10^{-1}
**7**	156992461	rs2366214	A/G	1.05	1.06	1.04	1.06	1.05	1.03	0.48	1.05	3.2×10^{-1}
**8**	10787612	rs4240673	T/C	1.04	1.03	1.00	1.04	1.02	1.05	0.89	0.99	8.5×10^{-1}
**8**	12618225	rs12680692	A/T	1.03	1.05	1.01	1.04	1.02	1.06	0.65	1.04	4.7×10^{-1}
**8**	36854711	rs10092900	G/T	1.04	1.06	1.04	1.03	1.03	1.06	0.16	1.05	4.3×10^{-1}

**8**	37397803	rs12680217	T/C	1.05	1.09	1.06	1.05	0.98	1.05	0.53	1.02	8.3×10^{-1}
**8**	41510260	rs12550613	C/G	1.04	1.07	1.05	1.03	1.01	1.04	0.72	0.96	4.6×10^{-1}
**8**	41522991	rs508419	G/A	1.05	1.03	1.07	1.05	1.06	1.06	0.92	0.98	7.7×10^{-1}
**8**	95965695	rs13257021	A/G	1.04	0.99	1.05	1.05	1.06	1.02	0.40	0.98	7.4×10^{-1}
**8**	116497173	rs800909	T/C	1.03	1.08	1.02	1.03	1.06	1.02	0.26	1.06	2.7×10^{-1}
**8**	118184783	rs13266634	C/T	1.12	1.08	1.13	1.11	1.10	1.11	0.87	1.15	5.9×10^{-2}
**8**	129569999	rs4733612	G/A	1.04	1.02	1.08	1.04	1.08	1.03	0.03	1.24	2.3×10^{-1}
**8**	145544720	rs3890400	A/G	1.04	1.04	1.04	1.05	1.04	1.04	0.11	1.03	6.8×10^{-1}
**8**	145972670	rs7014773	T/C	1.03	1.06	1.04	1.03	1.03	0.99	0.65	0.98	6.0×10^{-1}
**9**	4290085	rs4237150	C/G	1.05	1.01	1.06	1.04	1.09	1.07	0.68	1.12	2.8×10^{-2}*
**9**	4297892	rs4258054	T/C	1.03	1.00	1.03	1.04	1.03	1.01	0.24	0.91	9.6×10^{-2}
**9**	19074538	rs12380322	G/A	1.03	0.99	1.03	1.04	1.00	1.03	0.23	1.04	4.4×10^{-1}
**9**	21840834	rs7856455	G/T	1.04	0.98	1.04	1.10	1.01	1.03	0.76	1.03	6.8×10^{-1}
**9**	22133984	rs10757282	C/T	1.08	1.05	1.06	1.09	1.08	1.06	0.40	1.07	1.4×10^{-1}
**9**	22134094	rs10811661	T/C	1.17	1.08	1.16	1.19	1.15	1.20	0.92	1.22	3.2×10^{-2}*
**9**	28410683	rs1412234	C/T	1.03	1.07	1.01	1.04	1.02	1.06	0.22	0.98	6.5×10^{-1}
**9**	34074476	rs12001437	C/T	1.03	1.00	1.03	1.04	1.03	1.00	0.47	1.03	4.5×10^{-1}
**9**	81914978	rs13290396	C/T	1.10	1.12	1.12	1.10	1.09	1.10	0.70	1.09	8.6×10^{-2}
**9**	83998346	rs9332453	C/T	1.03	0.98	1.05	1.02	1.02	1.06	0.73	1.02	7.4×10^{-1}
**9**	84308948	rs2796441	G/A	1.07	1.01	1.08	1.07	1.06	1.05	0.45	1.03	4.9×10^{-1}
**9**	96971175	rs12345069	C/T	1.04	1.05	1.01	1.04	1.07	1.04	0.98	1.00	8.9×10^{-1}
**9**	98278413	rs113154802	C/T	1.05	1.05	1.07	1.03	1.24	1.08	0.97	1.12	3.8×10^{-1}
**9**	126015103	rs2416899	T/G	1.03	0.97	1.03	1.05	0.99	1.08	0.28	0.97	5.6×10^{-1}
**9**	136149229	rs505922	C/T	1.05	1.02	1.05	1.05	1.09	1.02	0.11	0.94	4.5×10^{-1}
**9**	139243334	rs28429551	A/T	1.09	1.08	1.16	1.08	1.08	1.10	0.72	1.14	1.9×10^{-2}*
**9**	139247229	rs74604683	C/T	1.05	1.23	1.06	1.05	1.08	1.03	0.87	0.97	7.8×10^{-1}
**10**	12307894	rs11257655	T/C	1.11	1.09	1.13	1.09	1.15	1.10	0.41	1.08	1.2×10^{-1}
**10**	26497704	rs7923442	A/G	1.04	1.04	1.04	1.03	1.03	1.05	0.81	1.07	2.2×10^{-1}
**10**	64974380	rs41274074	G/C	1.06	0.99	1.07	1.08	1.00	0.98	0.78	1.03	6.2×10^{-1}
**10**	71320943	rs190925	A/G	1.04	0.95	1.06	1.05	1.03	1.06	0.36	0.92	9.9×10^{-2}
**10**	71466578	rs2642588	G/T	1.05	0.99	1.16	1.05	1.01	1.07	0.70	1.00	8.6×10^{-1}

**10**	77244336	rs3012060	T/A	1.05	1.20	1.05	1.05	1.05	1.01	0.24	0.99	8.1×10^{-1}
**10**	80943841	rs703980	G/A	1.06	1.04	1.06	1.07	1.04	1.04	0.60	1.06	2.0×10^{-1}
**10**	89766368	rs10887775	A/G	1.04	1.01	1.04	1.04	1.02	1.07	0.29	1.02	6.9×10^{-1}
**10**	94460650	rs10882099	T/C	1.02	1.03	1.09	1.02	1.08	1.00	0.43	1.04	3.9×10^{-1}
**10**	94479107	rs1112718	A/G	1.02	0.99	1.07	1.01	1.01	1.02	0.40	1.04	5.0×10^{-1}
**10**	99056190	rs10748694	A/T	1.04	1.03	1.06	1.03	0.99	1.08	0.39	1.15	4.0×10^{-3}*
**10**	112621837	rs7067540	C/T	1.04	1.03	1.06	1.04	1.03	1.03	0.55	0.96	3.9×10^{-1}
**10**	114344288	rs12243296	G/A	1.04		1.22	1.03	1.09	1.00	0.00	0.69	5.6×10^{-1}
**10**	114381965	rs7100404	C/T	1.05		1.37	1.05	1.08	0.99	1.00	0.71	4.9×10^{-1}
**10**	114428364	rs2859885	C/T	1.05		1.01	1.07	0.97	1.00	0.60	1.04	4.5×10^{-1}
**10**	114552267	rs10787461	G/A	1.04		0.86	1.05	1.00	1.01	0.45	1.00	8.3×10^{-1}
**10**	114715598	rs2104598	G/A	1.04		1.21	1.04	1.11	1.00	0.67	1.07	2.0×10^{-1}
**10**	114758349	rs7903146	T/C	1.33		1.18	1.35	1.29	1.26	0.09	1.02	6.5×10^{-1}
**10**	114797893	rs7076754	G/A	1.07		1.14	1.06	1.14	1.04	0.97	0.92	6.2×10^{-1}
**10**	114859416	rs7081841	G/C	1.05		1.00	1.05	1.07	1.00	0.43	1.00	8.6×10^{-1}
**10**	115016408	rs12257761	T/C	1.06		1.03	1.09	1.00	1.02	0.70	1.10	5.5×10^{-2}
**10**	115069951	rs11196296	T/C	1.02		0.91	1.16	0.83	0.98	0.17	0.94	3.8×10^{-1}
**10**	115247447	rs11596522	T/G	1.05			1.07	0.86	1.01	0.02	1.03	8.2×10^{-1}
**10**	122834572	rs11199753	G/T	1.05	1.13	1.07	1.04	1.06	0.99	0.87	0.99	8.4×10^{-1}
**10**	122909625	rs2172073	A/C	1.05	1.06	1.04	1.07	1.07	1.04	0.72	1.06	3.7×10^{-1}
**10**	122968964	rs11592107	A/G	1.03	1.05	1.04	1.03	1.05	1.03	0.01	1.53	1.0×10^{-1}
**10**	124167512	rs2421016	C/T	1.04	0.96	1.04	1.05	1.07	1.04	0.09	1.02	8.3×10^{-1}
**11**	2077271	rs76547628	T/C	1.04	1.01	0.95	1.06	1.10	1.00	0.12	1.18	2.0×10^{-2}*
**11**	2194420	rs10770142	G/C	1.07	1.14	1.10	1.07	1.07	1.08	0.46	1.28	1.8×10^{-7}*
**11**	2235129	rs4930050	G/A	1.06	1.15	0.89	1.02	1.15	1.00	0.46	0.89	1.8×10^{-2}*
**11**	2364549	rs800125	A/C	1.01	0.91	0.99	1.03	0.94	1.00	0.52	1.04	4.6×10^{-1}
**11**	2375458	rs79495865	G/A	1.02	1.13	0.94	1.04	1.07	1.02	0.66	0.96	3.5×10^{-1}
**11**	2579163	rs2283164	A/G	1.09	1.16	1.08	1.11	1.01	1.10	0.99	0.86	6.8×10^{-1}
**11**	2681072	rs151215	G/A	1.04	1.04	0.96	1.06	1.06	1.05	0.05	1.08	4.6×10^{-1}
**11**	2691500	rs231361	A/G	1.09	1.12	1.04	1.10	1.12	1.08	0.66	0.91	9.7×10^{-2}
**11**	2799679	rs2237884	T/C	1.04	1.03	1.07	1.04	1.10	0.96	0.55	1.05	3.7×10^{-1}

**11**	2857897	rs234866	G/A	1.05	1.10	1.03	1.05	1.14	1.08	0.96	1.06	7.1×10^{-1}
**11**	2858546	rs2237897	C/T	1.15	1.15	1.12	1.21	1.23	1.20	0.55	1.32	2.2×10^{-8}*
**11**	2908754	rs445084	G/A	1.04	1.07	1.03	1.04	1.12	1.04	0.41	1.05	2.8×10^{-1}
**11**	8654528	rs10769936	C/T	1.03	1.02	1.03	1.04	1.06	1.00	0.79	1.00	8.4×10^{-1}
**11**	17408630	rs5215	C/T	1.08	1.05	1.09	1.07	1.07	1.07	0.39	1.06	2.2×10^{-1}
**11**	27683618	rs4923464	C/T	1.03	1.13	1.05	1.02	0.99	1.02	0.84	0.98	7.9×10^{-1}
**11**	32927778	rs145678014	G/T	1.10	1.20	0.51	1.11	1.07	0.95	0.99	1.98	4.4×10^{-2}*
**11**	43816200	rs6485462	C/T	1.03	1.02	1.00	1.05	1.04	1.02	0.53	1.00	8.5×10^{-1}
**11**	49477266	rs6485981	T/C	1.04	0.98	1.03	1.06	1.08	1.07	0.14	1.00	8.2×10^{-1}
**11**	65326154	rs12789028	A/G	1.06	1.06	1.04	1.06	1.03	1.08	0.10	1.22	1.6×10^{-2}*
**11**	72460398	rs77464186	A/C	1.11	1.07	1.15	1.11	1.09	1.09	0.95	1.13	2.8×10^{-1}
**11**	76156973	rs61894507	G/A	1.04	1.00	1.05	1.03	1.05	1.07	0.94	0.86	1.9×10^{-1}
**11**	92708710	rs10830963	G/C	1.08	1.15	1.06	1.11	1.11	1.09	0.14	1.06	4.5×10^{-1}
**11**	93131667	rs11020308	A/C	1.04	1.02	1.03	1.04	0.98	1.06	0.26	1.02	7.4×10^{-1}
**11**	128040810	rs10893827	A/G	1.04	1.03	1.03	1.06	1.06	1.05	0.65	1.03	6.4×10^{-1}
**11**	128235252	rs7104712	C/A	1.04	1.03	1.04	1.04	1.06	1.00	0.56	1.01	8.2×10^{-1}
**11**	128389391	rs11819995	T/C	1.05	1.03	1.04	1.05	1.02	1.05	0.15	1.12	1.2×10^{-1}
**12**	4033222	rs10848960	G/C	1.04	0.98	0.60	1.05	0.99	1.02	0.98	1.29	1.7×10^{-1}
**12**	4382324	rs3812821	G/C	1.05	0.96	1.21	1.05	1.07	1.04	0.59	1.14	3.6×10^{-3}*
**12**	26474867	rs10842708	G/A	1.04	1.07	1.03	1.05	1.01	1.04	0.69	1.01	8.5×10^{-1}
**12**	27964996	rs12578595	C/T	1.07	1.02	1.07	1.08	1.05	1.05	0.75	0.92	1.2×10^{-1}
**12**	33370406	rs6488140	A/G	1.04	1.09	1.01	1.05	1.06	1.03	0.69	1.08	1.4×10^{-1}
**12**	50263148	rs7132908	A/G	1.03	1.00	1.04	1.03	1.03	1.00	0.08	1.02	7.8×10^{-1}
**12**	66255005	rs343093	G/C	1.07	1.12	1.07	1.08	1.09	1.05	0.23	1.10	9.3×10^{-2}
**12**	66360164	rs7970350	T/C	1.04	1.00	1.04	1.05	1.01	1.03	0.82	0.99	7.7×10^{-1}
**12**	71449521	rs7313668	T/G	1.04	0.97	1.05	1.05	1.03	1.02	0.04	1.20	1.5×10^{-1}
**12**	97851611	rs7972074	C/T	1.04	1.06	1.04	1.02	1.10	1.01	0.82	1.06	2.9×10^{-1}
**12**	108629780	rs1426371	G/A	1.05	1.04	1.05	1.05	1.04	1.04	0.82	1.01	8.4×10^{-1}
**12**	118412373	rs34965774	A/G	1.06	1.05	1.07	1.06	1.06	1.05	0.32	1.03	6.5×10^{-1}
**12**	121456616	rs61953351	G/T	1.06	0.98	1.06	1.07	1.12	0.99	0.98	0.91	5.8×10^{-1}
**12**	123618544	rs1790116	T/G	1.04	1.03	1.03	1.04	1.03	1.07	0.55	1.00	7.1×10^{-1}

**12**	124545435	rs2451321	C/G	1.03	1.06	1.03	1.03	1.06	1.04	0.75	1.03	6.2×10^{-1}
**12**	133069698	rs12811407	A/G	1.05	1.04	1.06	1.05	1.07	1.01	0.10	1.17	4.6×10^{-2}*
**13**	23309382	rs314879	C/T	1.04	1.08	1.04	1.04	1.05	1.04	0.02	1.27	2.0×10^{-1}
**13**	26776999	rs34584161	A/G	1.05	1.00	1.06	1.05	1.02	1.04	0.34	1.06	2.8×10^{-1}
**13**	33554587	rs2858980	G/A	1.06	1.02	1.07	1.06	1.06	1.07	0.31	1.12	2.4×10^{-2}*
**13**	51096095	rs963740	A/T	1.04	1.03	1.04	1.04	1.04	1.02	0.90	1.12	1.8×10^{-1}
**13**	54107583	rs9568868	T/G	1.05	1.02	1.05	1.04	1.06	1.07	0.62	0.99	8.4×10^{-1}
**13**	80707429	rs1215468	A/G	1.08	1.06	1.09	1.08	1.11	1.08	0.61	1.07	1.6×10^{-1}
**13**	91942919	rs34165267	C/T	1.05	1.04	1.10	1.04	1.06	1.02	0.99	1.40	2.1×10^{-1}
**14**	33303540	rs12883788	T/C	1.04	1.00	1.04	1.04	1.04	1.03	0.39	1.03	5.5×10^{-1}
**14**	38803756	rs2183237	G/A	1.04	1.03	1.04	1.04	1.10	1.03	0.45	1.01	8.1×10^{-1}
**14**	79944099	rs8008910	A/G	1.06	1.06	1.26	1.06	1.07	1.03	0.31	1.02	6.8×10^{-1}
**14**	101124721	rs12878003	G/A	1.04	1.06	1.01	1.04	1.14	1.04	0.94	1.18	1.1×10^{-1}
**14**	101255172	rs73347525	A/G	1.05	1.05	1.07	1.05	1.07	1.02	0.63	1.10	7.9×10^{-2}
**14**	101301866	rs1053900	C/T	1.03	1.03	1.02	1.03	1.08	1.01	0.14	0.94	3.8×10^{-1}
**14**	103252270	rs11160699	A/G	1.04	1.03	1.04	1.04	1.08	1.05	0.54	1.09	8.2×10^{-2}
**15**	38843887	rs28582094	G/A	1.04	1.05	1.03	1.05	1.06	1.01	0.56	1.10	5.7×10^{-2}
**15**	40616742	rs3743140	A/G	1.05	1.04	1.09	1.03	1.07	1.02	0.26	1.00	8.3×10^{-1}
**15**	41818917	rs1473781	A/G	1.03	1.10	1.00	1.05	1.02	1.02	0.61	1.02	5.9×10^{-1}
**15**	52517714	rs3825801	C/T	1.05	1.00	1.04	1.05	0.95	1.08	0.96	0.92	4.7×10^{-1}
**15**	62391608	rs7163757	C/T	1.06	1.03	1.09	1.05	1.05	1.05	0.38	0.93	1.3×10^{-1}
**15**	63871292	rs7178762	C/T	1.04	1.02	1.04	1.04	1.06	1.03	0.79	0.95	3.9×10^{-1}
**15**	68080886	rs4776970	A/T	1.04	1.00	1.05	1.04	1.03	1.03	0.10	1.07	4.7×10^{-1}
**15**	75815758	rs11636031	T/C	1.05	1.04	1.05	1.05	1.07	1.03	0.86	1.13	9.1×10^{-2}
**15**	77776562	rs952472	C/A	1.08	1.06	1.08	1.08	1.08	1.06	0.65	1.07	2.4×10^{-1}
**15**	90379632	rs6496609	C/A	1.07	1.13	1.06	1.07	1.01	1.06	0.08	1.19	6.6×10^{-2}
**15**	91513157	rs2890156	A/T	1.07	1.06	1.08	1.07	1.05	1.08	0.39	1.01	7.7×10^{-1}
**15**	93832067	rs7167984	G/A	1.04	1.04	1.06	1.03	1.05	1.01	0.53	1.05	2.9×10^{-1}
**16**	295795	rs6600191	T/C	1.04	1.04	1.03	1.06	1.02	1.04	0.47	1.04	4.8×10^{-1}
**16**	3613126	rs12445430	T/C	1.04	1.06	1.04	1.04	1.06	1.03	0.18	1.10	1.6×10^{-1}
**16**	53809123	rs55872725	T/C	1.13	1.11	1.15	1.13	1.15	1.07	0.15	1.15	4.8×10^{-2}*

**16**	69651866	rs862320	C/T	1.04	1.02	1.03	1.04	1.02	1.01	0.88	0.88	7.3×10^{-2}
**16**	73100308	rs6416749	C/T	1.04	1.01	1.06	1.03	1.05	1.01	0.21	1.01	8.4×10^{-1}
**16**	75243657	rs72802358	G/C	1.09	0.99	1.10	1.12	1.07	1.10	0.95	1.21	8.3×10^{-2}
**16**	81534790	rs2925979	T/C	1.05	1.03	1.04	1.06	1.02	1.08	0.07	0.97	6.8×10^{-1}
**16**	88554480	rs9937296	C/T	1.04	0.99	1.04	1.05	1.07	1.02	0.53	1.02	8.0×10^{-1}
**17**	3828086	rs1043246	G/C	1.05	1.00	1.04	1.05	1.04	1.08	0.57	1.00	7.9×10^{-1}
**17**	3988451	rs8071043	C/T	1.04	1.07	0.99	1.05	1.03	1.02	0.66	0.97	5.7×10^{-1}
**17**	6953155	rs113748381	A/G	1.11	1.08	1.13	1.08	1.13	0.81	0.40	1.02	7.0×10^{-1}
**17**	17751478	rs1108646	A/G	1.04	0.99	1.04	1.04	1.05	1.04	0.64	1.01	8.4×10^{-1}
**17**	29704002	rs1048317	T/C	1.04	1.04	1.05	1.03	1.06	1.02	0.83	1.15	2.3×10^{-2}*
**17**	36043653	rs3094515	C/T	1.04	1.06	1.03	1.05	1.04	1.02	0.75	1.11	5.1×10^{-2}
**17**	36056076	rs12449654	C/G	1.05	1.04	1.08	1.04	1.12	1.01	0.45	1.05	4.0×10^{-1}
**17**	36099952	rs10908278	T/A	1.09	1.00	1.14	1.08	1.04	1.08	0.33	1.09	9.6×10^{-2}
**17**	40696915	rs684214	T/C	1.04	1.08	1.03	1.05	1.04	1.02	0.05	1.07	5.3×10^{-1}
**17**	47060322	rs35895680	C/A	1.05	1.04	1.04	1.06	1.01	1.02	0.74	1.02	7.3×10^{-1}
**17**	62203128	rs57676627	T/C	1.06	1.02	1.41	1.06	1.07	1.09	0.02	0.99	8.6×10^{-1}
**17**	65957568	rs9899520	A/G	1.04	0.97	1.04	1.05	1.01	1.04	0.38	1.06	2.7×10^{-1}
**17**	76792179	rs1044486	G/A	1.04	1.07	1.04	1.03	1.06	1.04	0.56	1.08	1.4×10^{-1}
**18**	7076836	rs9948462	T/C	1.04	0.99	1.05	1.04	1.03	1.05	0.72	0.98	7.2×10^{-1}
**18**	56876430	rs9957320	G/T	1.05	1.00	1.02	1.06	1.09	1.04	0.58	1.05	3.8×10^{-1}
**18**	57829135	rs6567160	C/T	1.07	1.08	1.09	1.06	1.13	1.06	0.03	0.84	3.3×10^{-1}
**18**	60845884	rs12454712	T/C	1.05	1.02	1.05	1.05	1.06	1.03	0.64	1.05	3.0×10^{-1}
**19**	4951064	rs262549	G/C	1.05	1.03	1.05	1.05	1.05	1.04	0.09	1.10	2.9×10^{-1}
**19**	7968168	rs2115107	A/G	1.05	1.07	1.05	1.05	1.04	1.02	0.33	1.01	8.2×10^{-1}
**19**	12509536	rs4804181	A/C	1.04	1.01	1.04	1.04	1.04	1.03	0.66	1.02	6.9×10^{-1}
**19**	19379549	rs58542926	T/C	1.07	1.05	1.03	1.09	1.12	1.04	0.02	1.07	7.2×10^{-1}
**19**	33890838	rs10406327	C/G	1.04	1.02	1.07	1.04	1.00	1.03	0.76	1.02	6.8×10^{-1}
**19**	45326768	rs1871045	T/C	1.03	1.04	1.04	1.03	1.04	1.05	0.38	1.08	1.1×10^{-1}
**19**	45411941	rs429358	T/C	1.06	1.04	1.03	1.07	1.05	1.04	0.85	0.88	7.1×10^{-2}
**19**	46157019	rs10406431	A/G	1.05	1.02	1.07	1.05	1.02	1.05	0.46	1.00	9.2×10^{-1}
**19**	46178661	rs2238689	C/T	1.04	1.00	1.04	1.04	1.03	1.03	0.42	1.06	2.3×10^{-1}

**19**	47569003	rs3810291	A/G	1.05	1.05	1.04	1.04	1.09	1.04	0.63	1.05	4.4×10^{-1}
**20**	22427370	rs2181063	C/G	1.03	1.03	1.05	1.03	0.98	1.02	0.83	1.05	4.5×10^{-1}
**20**	32674967	rs4911405	T/C	1.04	1.08	1.01	1.04	1.07	1.03	0.65	1.04	4.2×10^{-1}
**20**	42994812	rs12625671	C/T	1.08	1.14	1.08	1.08	1.08	1.11	0.81	1.05	5.1×10^{-1}
**20**	43042364	rs1800961	T/C	1.16	1.10	1.15	1.18	1.14	1.07	0.03	1.39	1.4×10^{-2}*
**20**	45596378	rs6063046	A/G	1.04	1.07	1.03	1.05	1.04	1.03	0.84	1.16	2.9×10^{-2}*
**20**	48832020	rs6091115	T/C	1.05	1.04	1.05	1.04	1.06	1.05	0.52	1.06	2.3×10^{-1}
**20**	57387352	rs736266	T/A	1.03	1.03	1.03	1.04	1.02	0.99	0.70	1.07	2.4×10^{-1}
**22**	30205572	rs36575	C/T	1.08	1.08	0.80	1.08	1.08	1.07	0.99	0.96	8.1×10^{-1}
**22**	32203334	rs75307421	A/G	1.10	1.06	1.06	1.11	1.16	1.08	0.21	1.15	2.3×10^{-2}*
**22**	44324730	rs738408	T/C	1.03	1.03	1.02	1.04	1.06	1.02	0.81	0.96	4.5×10^{-1}
**22**	50356302	rs28691713	C/T	1.05	1.06	1.08	1.04	1.00	1.03	0.60	1.03	5.6×10^{-1}

[a] Chr is chromosome.
[b] Position is in base pairs (bp) on Genome Reference Consortium Human Build 37.
[c] A1/a2 are the alleles from DIAMANTE 2022, with the effect allele given first.
[d] OR is reported per copy of the effect allele, with all but OR_pop calculated for participants within each ancestry group included in the DIAMANTE 2022 GWAS meta-analysis: OR_comp for the multi-ancestry composite PS, OR_afr for the multi-ancestry African PS, OR_eas for the multi-ancestry East Asian PS, OR_eur for the multi-ancestry European PS, OR_his for the multi-ancestry Latino/Hispanic PS, OR_sas for the multi-ancestry South Asian PS. OR is not reported for all SNPs across analyses for various ancestry groups: see DIAMANTE 2022[9] methodologies for details.
[e] Freq_pop is the frequency of a1 in the present Indigenous study population.
[f] OR_pop is the OR calculated and cross-validated for the population-specific weight score using the same set of variants as the multi-ancestry composite PS.
*Indicates nominally significant (*p*<0.05), and directionally consistent with multi-ancestry result, association in the current study population.

Table S6. SNPs included in the population-specific variant PS.

chr[a]	position[b]	SNP	subsets[c]	a1/a2[d]	a1f[e]	OR[f]	*p*-value
**1**	3637812	rs2181487	1>2	C/A	0.41	1.28	9.9×10^{-5}
**1**	3675959	rs12131045	1>2	G/T	0.32	1.37	5.0×10^{-6}
**1**	7532704	rs1474950912	1>2	C/G	0.94	1.62	2.5×10^{-4}
**1**	10201624	rs77567553	1>2	A/G	0.85	1.36	5.5×10^{-4}
**1**	16322282	rs1024537880	2>1	G/A	0.08	1.65	2.0×10^{-5}
**1**	18793386	rs12041192	2>1	A/G	0.07	1.55	4.4×10^{-4}
**1**	20610244	rs55928751	2>1	A/G	0.26	1.28	6.0×10^{-4}
**1**	38590813	rs898985	1>2	C/T	0.76	1.29	5.5×10^{-4}
**1**	39130160	rs4474214	1>2	G/T	0.84	1.36	4.1×10^{-4}
**1**	58273678	rs72664686	2>1	T/G	0.49	1.25	4.2×10^{-4}
**1**	63673963	rs147237461	2>1	A/G	0.25	1.33	8.0×10^{-5}
**1**	63722576	rs141607445	2>1	G/A	0.25	1.35	4.0×10^{-5}
**1**	66602990	rs551000	1>2	C/T	0.12	1.43	2.8×10^{-4}
**1**	67826180	rs114967702	2>1	A/G	0.76	1.35	3.4×10^{-5}
**1**	67983243	rs10789236	1>2	C/T	0.47	1.28	1.7×10^{-4}
**1**	68225671	rs554355	1>2	A/G	0.20	1.32	4.6×10^{-4}
**1**	69459021	rs199961703	1>2	I/D	0.86	1.38	5.6×10^{-4}
**1**	71093805	rs8179355	1>2	C/A	0.63	1.33	2.2×10^{-5}
**1**	71172305	rs76562719	1>2	G/A	0.66	1.31	5.7×10^{-5}
**1**	89948844	rs9427984	1>2	A/G	0.59	1.26	4.7×10^{-4}
**1**	91402075	rs358694	1>2	C/T	0.36	1.28	2.2×10^{-4}
**1**	91526198	rs2625760	1>2	A/G	0.40	1.32	2.3×10^{-5}
**1**	98141714	rs189939377	2>1	C/T	0.07	1.52	4.5×10^{-4}
**1**	111727851	chr1:111727851	2>1	A/C	0.96	1.86	6.1×10^{-5}
**1**	115721655	rs10858066	2>1	C/T	0.55	1.29	9.8×10^{-5}
**1**	116403499	rs975972	1>2	C/T	0.20	1.34	2.3×10^{-4}
**1**	164492700	rs6677872	1>2	T/G	0.93	1.60	1.8×10^{-4}
**1**	169455435	rs2056926	2>1	C/G	0.20	1.39	1.3×10^{-5}

**1**	169529973	rs6029	2>1	T/C	0.19	1.39	$3.0×10^{-5}$
**1**	183709624	rs12407737	1>2	C/A	0.95	1.67	$3.5×10^{-4}$
**1**	183747518	rs78151551	1>2	A/G	0.95	1.67	$3.3×10^{-4}$
**1**	187743426	rs115672901	1>2	T/G	0.90	1.45	$4.3×10^{-4}$
**1**	193504097	rs12034778	2>1	T/A	0.84	1.36	$3.1×10^{-4}$
**1**	194775338	rs6656153	2>1	T/C	0.08	1.47	$5.2×10^{-4}$
**1**	202114469	rs11581162	1>2	G/A	0.10	1.48	$3.3×10^{-4}$
**1**	221303867	rs10863588	1>2	C/T	0.12	1.44	$2.7×10^{-4}$
**1**	230011750	rs149517808	1>2	T/C	0.14	1.38	$5.3×10^{-4}$
**1**	230486870	rs74634891	1>2	G/A	0.88	1.55	$6.6×10^{-6}$
**1**	230619350	rs12048353	1>2	C/T	0.34	1.31	$9.2×10^{-5}$
**1**	237215582	chr1:237215582	1>2	G/T	0.04	1.78	$4.7×10^{-4}$
**1**	237326254	rs111689687	1>2	G/T	0.05	1.78	$9.3×10^{-5}$
**1**	247363038	rs12130823	1>2	G/T	0.25	1.29	$4.1×10^{-4}$
**2**	3457459	rs12619324	1>2	G/A	0.83	1.34	$5.4×10^{-4}$
**2**	5689487	rs2882275	1>2	T/C	0.28	1.28	$4.1×10^{-4}$
**2**	16277283	rs138932039	2>1	C/T	0.26	1.33	$7.8×10^{-5}$
**2**	16336621	rs144177256	2>1	C/G	0.21	1.37	$6.3×10^{-5}$
**2**	28819516	rs10194430	1>2	A/G	0.34	1.26	$6.4×10^{-4}$
**2**	33243179	rs75886200	1>2	G/C	0.07	1.64	$8.6×10^{-5}$
**2**	33267614	rs13393464	2>1	T/C	0.88	1.42	$1.3×10^{-4}$
**2**	33626885	rs78541119	1>2	G/A	0.04	1.90	$2.2×10^{-4}$
**2**	33626957	rs609277	2>1	T/G	0.38	1.25	$6.2×10^{-4}$
**2**	42114002	rs13028888	2>1	C/T	0.39	1.25	$4.6×10^{-4}$
**2**	45572181	rs13036196	1>2	T/C	0.76	1.31	$2.5×10^{-4}$
**2**	45668873	rs3770252	1>2	G/T	0.81	1.32	$5.7×10^{-4}$
**2**	49641556	rs17835319	2>1	A/G	0.20	1.34	$2.4×10^{-4}$
**2**	55090468	rs35459932	2>1	A/G	0.84	1.37	$2.2×10^{-4}$
**2**	98481711	rs139613671	1>2	C/T	0.23	1.33	$1.8×10^{-4}$
**2**	98647080	rs143198148	1>2	C/G	0.19	1.33	$5.2×10^{-4}$
**2**	100862903	rs6737502	1>2	C/T	0.78	1.35	$1.9×10^{-4}$

**2**	106401187	chr2:106401187	2>1	D/I	0.01	2.79	5.1×10^{-4}
**2**	110282530	chr2:110282530	2>1	T/G	0.95	1.71	5.7×10^{-4}
**2**	111413674	chr2:111413674	2>1	G/A	0.96	1.82	2.9×10^{-4}
**2**	111881704	chr2:111881704	2>1	C/T	0.96	1.83	2.2×10^{-4}
**2**	121084993	rs73951213	2>1	A/G	0.92	1.50	2.8×10^{-4}
**2**	129213748	rs10175448	2>1	T/C	0.41	1.24	5.7×10^{-4}
**2**	130178361	rs66638135	2>1	I/D	0.67	1.26	4.8×10^{-4}
**2**	170981031	rs4668198	1>2	C/T	0.62	1.26	4.5×10^{-4}
**2**	174267408	rs10194659	1>2	C/G	0.31	1.34	3.1×10^{-5}
**2**	180454209	rs79903308	1>2	A/G	0.07	1.63	1.2×10^{-4}
**2**	180543780	rs1515286	1>2	A/C	0.18	1.42	2.2×10^{-5}
**2**	198169130	rs12464356	1>2	T/C	0.88	1.46	1.3×10^{-4}
**2**	198298448	rs150021501	1>2	I/D	0.88	1.41	4.5×10^{-4}
**2**	198515254	rs116121900	1>2	G/A	0.86	1.40	2.7×10^{-4}
**2**	199679110	rs10189905	1>2	G/T	0.24	1.31	4.3×10^{-4}
**2**	207412722	rs3217266	2>1	I/D	0.80	1.37	4.2×10^{-5}
**2**	207518184	rs10186535	2>1	T/C	0.78	1.29	5.9×10^{-4}
**2**	208333760	chr2:208333760	2>1	C/T	0.95	1.67	4.3×10^{-4}
**2**	215243598	rs6705674	1>2	C/T	0.41	1.26	3.6×10^{-4}
**2**	215401934	rs77091556	1>2	T/G	0.27	1.33	8.1×10^{-5}
**2**	216023682	rs7419736	1>2	G/A	0.75	1.35	6.1×10^{-5}
**2**	216138939	rs112349663	1>2	I/D	0.80	1.39	4.6×10^{-5}
**2**	220821732	rs80349138	1>2	A/C	0.84	1.39	1.4×10^{-4}
**2**	221639854	rs6755421	2>1	A/G	0.64	1.26	4.3×10^{-4}
**2**	222027666	rs2019083	1>2	C/T	0.59	1.25	5.8×10^{-4}
**2**	225105625	chr2:225105625	1>2	G/C	0.96	1.79	3.2×10^{-4}
**2**	226360771	rs1963400	2>1	T/G	0.80	1.30	5.7×10^{-4}
**2**	226857138	rs138124232	1>2	C/A	0.87	1.39	5.9×10^{-4}
**2**	226979415	rs139975434	1>2	A/G	0.83	1.35	6.2×10^{-4}
**2**	227131174	rs41504645	1>2	T/C	0.86	1.39	3.8×10^{-4}
**2**	230284815	rs2396663	2>1	T/C	0.72	1.28	4.5×10^{-4}

**2**	232190769	rs56678553	1>2	A/G	0.65	1.32	3.8×10^{-5}
**2**	239775835	rs150530918	1>2	G/A	0.12	1.43	3.1×10^{-4}
**2**	242490658	rs13408032	1>2	T/C	0.47	1.25	4.6×10^{-4}
**2**	242577950	rs17140248	1>2	A/G	0.39	1.25	4.8×10^{-4}
**3**	1293504	rs3772339	1>2	A/C	0.29	1.29	3.3×10^{-4}
**3**	7589639	rs117534611	2>1	A/G	0.14	1.42	1.0×10^{-4}
**3**	9977244	rs3894571	2>1	T/C	0.30	1.30	9.0×10^{-5}
**3**	15382870	rs2103079	1>2	T/C	0.77	1.32	1.7×10^{-4}
**3**	39844963	rs62261638	1>2	G/A	0.33	1.31	1.0×10^{-4}
**3**	39966748	rs11717034	1>2	G/A	0.30	1.30	1.8×10^{-4}
**3**	57038055	rs183677269	2>1	C/A	0.86	1.36	4.8×10^{-4}
**3**	57151483	rs189430546	2>1	T/C	0.86	1.37	5.0×10^{-4}
**3**	58822664	rs79407372	1>2	G/A	0.87	1.40	4.6×10^{-4}
**3**	59777427	rs67777617	1>2	G/T	0.56	1.25	4.0×10^{-4}
**3**	60742058	rs13063937	1>2	C/A	0.73	1.31	1.8×10^{-4}
**3**	60833504	rs76349782	1>2	T/A	0.86	1.43	9.2×10^{-5}
**3**	62531625	rs17695565	1>2	T/C	0.82	1.38	1.3×10^{-4}
**3**	64835496	rs73119726	2>1	C/T	0.87	1.45	7.1×10^{-5}
**3**	65023127	rs4688534	2>1	C/A	0.24	1.29	4.8×10^{-4}
**3**	73701944	rs6781766	1>2	T/C	0.33	1.27	3.8×10^{-4}
**3**	73860347	rs61663855	1>2	D/I	0.13	1.39	5.4×10^{-4}
**3**	89994954	chr3:89994954	2>1	C/T	0.07	1.53	4.6×10^{-4}
**3**	96503627	rs75849815	1>2	T/A	0.10	1.45	5.8×10^{-4}
**3**	98562718	rs79834212	1>2	G/C	0.41	1.28	1.8×10^{-4}
**3**	98736435	rs80040807	1>2	A/C	0.38	1.27	3.3×10^{-4}
**3**	105005089	rs16851027	1>2	C/T	0.30	1.29	3.6×10^{-4}
**3**	105047616	rs10575418	1>2	I/D	0.63	1.26	5.0×10^{-4}
**3**	108652222	rs5851624	1>2	D/I	0.31	1.27	5.5×10^{-4}
**3**	112556904	rs6795521	2>1	G/C	0.31	1.30	9.0×10^{-5}
**3**	112914615	rs972554	2>1	A/G	0.32	1.25	6.1×10^{-4}
**3**	116317395	rs1518327	1>2	T/G	0.33	1.29	1.7×10^{-4}

**3**	131374802	rs9861011	1>2	G/A	0.73	1.29	4.6×10^{-4}
**3**	131835569	rs13074860	2>1	C/T	0.15	1.37	3.6×10^{-4}
**3**	133258597	rs4533654	1>2	T/G	0.73	1.32	8.2×10^{-5}
**3**	140296782	rs75476344	2>1	A/G	0.20	1.30	5.9×10^{-4}
**3**	141031298	rs79507063	2>1	T/G	0.82	1.34	3.7×10^{-4}
**3**	143806538	rs16854922	1>2	G/C	0.88	1.41	5.2×10^{-4}
**3**	148122713	rs4321499	2>1	G/T	0.21	1.32	3.9×10^{-4}
**3**	149050925	rs1526786	2>1	T/C	0.24	1.29	3.1×10^{-4}
**3**	167082105	rs79573605	2>1	G/C	0.10	1.44	4.4×10^{-4}
**3**	172707771	rs76050108	1>2	A/T	0.92	1.58	1.2×10^{-4}
**3**	174762060	rs9863629	2>1	T/C	0.86	1.40	1.6×10^{-4}
**3**	176927519	rs62296619	2>1	C/T	0.78	1.30	3.4×10^{-4}
**3**	181080833	rs118012541	2>1	G/A	0.84	1.37	2.5×10^{-4}
**3**	181505245	rs74909304	1>2	C/A	0.46	1.31	3.9×10^{-5}
**3**	184118600	rs7648852	1>2	G/A	0.40	1.30	6.9×10^{-5}
**3**	185680144	rs10937233	1>2	T/C	0.30	1.27	6.0×10^{-4}
**3**	185877745	rs138925570	1>2	G/A	0.93	1.61	1.0×10^{-4}
**3**	186346272	rs73061438	1>2	G/A	0.42	1.28	1.8×10^{-4}
**3**	188149586	rs7649407	2>1	A/G	0.87	1.38	4.8×10^{-4}
**3**	189048167	rs4687038	2>1	C/T	0.34	1.32	2.9×10^{-5}
**3**	194400812	rs9820108	1>2	A/G	0.33	1.27	5.7×10^{-4}
**4**	3682614	chr4:3682614	2>1	T/C	0.90	1.42	6.4×10^{-4}
**4**	8755903	rs140893109	2>1	G/A	0.94	1.62	2.4×10^{-4}
**4**	9902230	rs4235344	2>1	A/G	0.13	1.38	4.8×10^{-4}
**4**	10535135	rs117906214	2>1	G/A	0.20	1.30	5.4×10^{-4}
**4**	11572999	rs2191065	2>1	G/A	0.89	1.42	4.1×10^{-4}
**4**	14865904	rs2047203	2>1	A/T	0.19	1.32	6.6×10^{-4}
**4**	24280616	rs59580288	2>1	D/I	0.22	1.32	2.0×10^{-4}
**4**	33764638	rs144917101	1>2	G/A	0.88	1.44	2.1×10^{-4}
**4**	34075738	rs116018934	1>2	A/C	0.89	1.42	3.8×10^{-4}
**4**	42123860	rs137880547	1>2	T/C	0.91	1.50	5.0×10^{-4}

**4**	68091816	rs28528999	2>1	T/C	0.72	1.27	4.3×10^{-4}
**4**	78552172	rs28884780	1>2	A/G	0.34	1.32	4.3×10^{-5}
**4**	84052440	rs41332145	1>2	C/T	0.70	1.27	4.7×10^{-4}
**4**	90095095	rs180910763	1>2	C/T	0.80	1.34	2.4×10^{-4}
**4**	91765642	rs78416641	1>2	A/C	0.05	1.68	2.2×10^{-4}
**4**	121486977	rs36072791	1>2	A/G	0.82	1.32	6.2×10^{-4}
**4**	126112363	rs186374925	1>2	G/T	0.95	1.74	1.3×10^{-4}
**4**	136750399	rs13139177	2>1	G/A	0.59	1.24	6.3×10^{-4}
**4**	138486135	rs188206537	2>1	A/G	0.17	1.50	1.6×10^{-6}
**4**	138534031	rs141998354	2>1	T/C	0.19	1.45	4.6×10^{-6}
**4**	148175509	rs181321827	1>2	A/G	0.88	1.49	7.7×10^{-5}
**4**	166861524	rs28669377	2>1	T/A	0.90	1.50	2.0×10^{-4}
**4**	168716957	rs191684316	2>1	C/T	0.06	1.80	1.5×10^{-5}
**4**	178830486	rs188918690	2>1	G/C	0.11	1.41	5.9×10^{-4}
**4**	184835242	rs10866265	1>2	C/T	0.31	1.27	5.8×10^{-4}
**5**	2089792	rs139754007	2>1	D/I	0.38	1.24	6.3×10^{-4}
**5**	2902999	rs2860294	2>1	A/G	0.50	1.25	2.9×10^{-4}
**5**	18835357	rs114710558	1>2	T/C	0.12	1.40	5.4×10^{-4}
**5**	19488982	rs13170473	2>1	G/A	0.44	1.24	5.3×10^{-4}
**5**	27367354	rs59371379	1>2	I/D	0.87	1.42	1.4×10^{-4}
**5**	27432606	rs4367265	1>2	A/G	0.86	1.42	1.3×10^{-4}
**5**	27609865	rs55668326	1>2	T/G	0.85	1.45	4.0×10^{-5}
**5**	31660006	rs6861360	1>2	G/A	0.36	1.28	2.0×10^{-4}
**5**	67169005	rs141412809	2>1	C/G	0.10	1.45	4.6×10^{-4}
**5**	67950535	rs10076830	2>1	T/C	0.94	1.61	1.9×10^{-4}
**5**	73221549	rs6453032	2>1	G/T	0.47	1.27	1.1×10^{-4}
**5**	98388026	rs59910509	2>1	A/G	0.12	1.38	6.4×10^{-4}
**5**	109204107	rs2241690	2>1	T/G	0.66	1.26	4.4×10^{-4}
**5**	109947984	rs117569584	2>1	G/A	0.94	1.57	4.7×10^{-4}
**5**	118705545	rs32652	2>1	T/G	0.38	1.27	2.0×10^{-4}
**5**	128130002	rs62391237	2>1	C/T	0.93	1.61	6.9×10^{-5}

**5**	128313080	rs2577423	2>1	T/C	0.71	1.32	6.5×10^{-5}
**5**	129700024	rs73239262	2>1	T/C	0.95	1.66	1.6×10^{-4}
**5**	130810992	rs149151967	2>1	G/T	0.95	1.64	6.5×10^{-4}
**5**	135215554	rs2269927	2>1	C/T	0.92	1.49	4.1×10^{-4}
**5**	136932292	chr5:136932292	2>1	C/T	0.05	1.73	2.1×10^{-4}
**5**	150881537	rs58610768	2>1	G/A	0.86	1.36	5.6×10^{-4}
**5**	163661781	rs73799291	2>1	G/C	0.06	1.59	3.7×10^{-4}
**5**	173273681	rs3849720	2>1	T/C	0.23	1.39	1.6×10^{-5}
**6**	3030410	rs6596934	1>2	C/G	0.29	1.28	5.5×10^{-4}
**6**	9512507	rs2327189	1>2	T/C	0.29	1.29	3.4×10^{-4}
**6**	15936733	rs6936104	2>1	G/A	0.91	1.43	6.2×10^{-4}
**6**	17850142	rs676296	1>2	A/G	0.79	1.31	5.3×10^{-4}
**6**	21506530	rs6927607	2>1	T/C	0.75	1.29	4.1×10^{-4}
**6**	24270120	rs117831339	1>2	G/A	0.19	1.32	6.0×10^{-4}
**6**	31481299	rs2516399	1>2	A/G	0.84	1.35	4.6×10^{-4}
**6**	37649437	rs62398397	1>2	G/A	0.55	1.27	2.6×10^{-4}
**6**	40547677	rs115546979	1>2	T/C	0.94	1.64	2.6×10^{-4}
**6**	43233482	chr6:43233482	2>1	T/C	0.99	3.27	3.9×10^{-5}
**6**	43413575	rs369686699	2>1	G/A	0.99	3.26	4.5×10^{-5}
**6**	45404230	rs1997992	1>2	T/C	0.29	1.31	1.2×10^{-4}
**6**	45922402	rs3777591	1>2	G/A	0.23	1.30	5.2×10^{-4}
**6**	64732017	rs9344660	1>2	G/A	0.09	1.48	4.9×10^{-4}
**6**	70767320	rs3805997	1>2	G/A	0.25	1.31	3.2×10^{-4}
**6**	79912072	chr6:79912072	1>2	G/T	0.03	2.03	4.7×10^{-4}
**6**	80178863	rs2803183	1>2	T/C	0.42	1.26	4.6×10^{-4}
**6**	93467089	chr6:93467089	1>2	A/C	0.05	1.64	5.3×10^{-4}
**6**	101940762	rs2579944	1>2	A/G	0.74	1.32	1.5×10^{-4}
**6**	103295786	chr6:103295786	1>2	G/A	0.96	1.85	2.7×10^{-4}
**6**	117832836	rs9387485	1>2	C/T	0.10	1.52	8.2×10^{-5}
**6**	139039029	rs9389610	1>2	G/A	0.57	1.26	3.8×10^{-4}
**6**	139759781	rs3010312	2>1	C/A	0.63	1.25	4.4×10^{-4}

**6**	151353805	rs12183135	1>2	C/G	0.06	1.63	4.7×10^{-4}
**6**	154668204	rs4870278	2>1	T/C	0.82	1.34	2.8×10^{-4}
**6**	155315556	rs13199643	1>2	G/A	0.37	1.33	1.2×10^{-5}
**6**	156494674	rs7774516	1>2	C/G	0.46	1.30	3.5×10^{-5}
**6**	156549813	rs9384441	1>2	A/G	0.24	1.40	8.8×10^{-6}
**6**	158295488	rs181880963	2>1	T/A	0.94	1.57	4.5×10^{-4}
**6**	164360807	rs12193589	1>2	A/G	0.40	1.27	2.4×10^{-4}
**6**	166896330	rs6929010	1>2	T/C	0.46	1.25	5.4×10^{-4}
**7**	1632513	rs13235561	1>2	A/C	0.16	1.38	1.7×10^{-4}
**7**	3078035	rs35049983	1>2	A/G	0.44	1.25	4.4×10^{-4}
**7**	3252555	rs34932678	2>1	A/G	0.67	1.32	2.7×10^{-5}
**7**	4224116	chr7:4224116	2>1	A/G	0.02	2.35	4.4×10^{-4}
**7**	7187816	rs150130341	2>1	T/C	0.92	1.52	4.2×10^{-4}
**7**	8189620	rs3757524	1>2	C/T	0.21	1.32	3.7×10^{-4}
**7**	9619380	rs2259306	2>1	C/T	0.58	1.24	5.5×10^{-4}
**7**	13907827	rs251994	2>1	C/T	0.53	1.24	3.7×10^{-4}
**7**	13945127	rs2237295	2>1	C/T	0.41	1.25	3.8×10^{-4}
**7**	20021609	rs6956048	1>2	C/A	0.52	1.25	4.7×10^{-4}
**7**	23997775	rs57329175	2>1	C/T	0.93	1.52	5.8×10^{-4}
**7**	24958042	rs120	2>1	A/G	0.25	1.30	1.9×10^{-4}
**7**	26122034	rs17153193	2>1	T/C	0.70	1.30	1.3×10^{-4}
**7**	28485676	rs17156696	2>1	T/A	0.32	1.26	5.3×10^{-4}
**7**	28547048	rs12333835	1>2	C/T	0.23	1.33	1.8×10^{-4}
**7**	29668519	rs16875105	2>1	A/G	0.19	1.31	4.6×10^{-4}
**7**	38673833	rs9918553	2>1	A/G	0.67	1.25	6.7×10^{-4}
**7**	43272084	rs181157662	2>1	T/C	0.18	1.35	1.8×10^{-4}
**7**	45982233	chr7:45982233	2>1	A/G	0.06	1.59	4.2×10^{-4}
**7**	51682401	rs4947627	1>2	A/G	0.16	1.36	3.9×10^{-4}
**7**	73141464	rs2030922	1>2	C/T	0.47	1.29	5.8×10^{-5}
**7**	93816686	rs80145019	1>2	C/A	0.29	1.30	2.0×10^{-4}
**7**	95897827	rs142857687	1>2	A/T	0.18	1.33	5.5×10^{-4}

**7**	103023537	rs10268808	1>2	A/C	0.34	1.26	5.2×10^{-4}
**7**	103193651	rs2711879	1>2	C/T	0.40	1.29	7.2×10^{-5}
**7**	121304227	rs187383253	2>1	C/A	0.91	1.48	2.2×10^{-4}
**7**	130404776	rs4067228	1>2	A/G	0.50	1.25	4.7×10^{-4}
**7**	135575461	rs62489095	2>1	C/A	0.07	1.53	3.4×10^{-4}
**7**	143602779	rs6947163	1>2	G/T	0.15	1.38	3.7×10^{-4}
**7**	149988544	rs147486244	2>1	G/A	0.63	1.28	1.4×10^{-4}
**8**	2838945	rs656466	2>1	C/G	0.12	1.39	5.5×10^{-4}
**8**	3026578	rs10503198	2>1	C/G	0.28	1.32	6.4×10^{-5}
**8**	3106370	rs79693896	2>1	T/A	0.88	1.40	5.5×10^{-4}
**8**	3290516	rs2406295	2>1	C/G	0.90	1.45	2.4×10^{-4}
**8**	3341226	rs79915250	2>1	C/T	0.93	1.52	4.2×10^{-4}
**8**	3904192	rs3849818	1>2	T/G	0.20	1.32	6.2×10^{-4}
**8**	6042400	rs1834203	2>1	T/C	0.50	1.27	1.3×10^{-4}
**8**	6709922	rs2951864	2>1	C/G	0.46	1.25	2.8×10^{-4}
**8**	8328109	rs2921039	1>2	G/C	0.11	1.44	3.3×10^{-4}
**8**	11439961	rs11250149	1>2	C/G	0.25	1.35	6.0×10^{-5}
**8**	12786947	chr8:12786947	1>2	T/C	0.05	1.75	2.1×10^{-4}
**8**	13209750	rs76989997	1>2	C/T	0.52	1.25	5.4×10^{-4}
**8**	13243084	rs1481679	1>2	A/G	0.55	1.26	4.2×10^{-4}
**8**	14443650	rs10086949	1>2	T/G	0.41	1.28	1.7×10^{-4}
**8**	27769687	rs35857051	1>2	T/C	0.39	1.25	6.0×10^{-4}
**8**	35956929	chr8:35956929	1>2	A/T	0.06	1.61	4.9×10^{-4}
**8**	55308198	rs12546306	1>2	C/T	0.68	1.27	6.2×10^{-4}
**8**	62495263	rs6471961	1>2	T/C	0.77	1.30	3.8×10^{-4}
**8**	62634563	rs149447795	1>2	T/C	0.19	1.37	1.7×10^{-4}
**8**	62663272	rs199622822	1>2	D/I	0.20	1.37	1.1×10^{-4}
**8**	70653945	rs182136050	2>1	T/G	0.16	1.38	1.3×10^{-4}
**8**	70692868	rs138718121	2>1	T/A	0.17	1.37	1.1×10^{-4}
**8**	81365072	chr8:81365072	1>2	A/G	0.93	1.67	1.1×10^{-4}
**8**	87327440	rs148141053	1>2	T/G	0.12	1.42	3.8×10^{-4}

**8**	89008404	rs180694224	2>1	A/G	0.92	1.48	5.1×10^{-4}
**8**	93586384	rs2595612	1>2	T/C	0.06	1.63	2.8×10^{-4}
**8**	99455167	rs201599771	1>2	D/I	0.88	1.47	7.2×10^{-5}
**8**	100002032	rs79434662	1>2	G/A	0.88	1.49	3.5×10^{-5}
**8**	100709154	rs140502344	1>2	A/G	0.87	1.41	2.9×10^{-4}
**8**	109912830	rs4734184	2>1	A/G	0.83	1.32	6.6×10^{-4}
**8**	111025983	rs55893742	1>2	A/G	0.03	2.08	5.5×10^{-5}
**8**	124987478	rs7465584	2>1	C/T	0.37	1.29	8.5×10^{-5}
**8**	136671060	rs4243847	2>1	A/G	0.41	1.25	4.8×10^{-4}
**8**	136682479	rs4075568	2>1	C/T	0.54	1.30	2.7×10^{-5}
**9**	538401	rs10975042	2>1	C/T	0.19	1.35	1.2×10^{-4}
**9**	4375454	rs2039194	1>2	G/C	0.82	1.33	6.0×10^{-4}
**9**	5498562	rs34719356	2>1	D/I	0.34	1.25	6.5×10^{-4}
**9**	8726153	rs1473822	2>1	C/T	0.66	1.28	1.6×10^{-4}
**9**	8924538	rs1368684	2>1	C/T	0.26	1.34	3.0×10^{-5}
**9**	9028285	rs324496	2>1	T/C	0.74	1.28	5.5×10^{-4}
**9**	9173304	rs968079	2>1	G/A	0.23	1.31	2.6×10^{-4}
**9**	9191639	rs62529522	2>1	T/C	0.68	1.30	7.5×10^{-5}
**9**	11876599	rs192161934	1>2	T/G	0.08	1.51	4.5×10^{-4}
**9**	25335125	rs113501731	1>2	A/C	0.15	1.42	7.5×10^{-5}
**9**	28393919	rs143327236	2>1	G/A	0.04	1.76	1.8×10^{-4}
**9**	29789814	rs950316	2>1	G/A	0.52	1.25	3.0×10^{-4}
**9**	32194564	rs188065424	2>1	G/A	0.75	1.31	2.2×10^{-4}
**9**	32320727	chr9:32320727	2>1	T/C	0.96	1.72	4.9×10^{-4}
**9**	80709044	rs62573096	2>1	G/A	0.26	1.29	4.5×10^{-4}
**9**	81158113	rs7027911	1>2	G/A	0.55	1.27	1.8×10^{-4}
**9**	87132349	rs35342431	2>1	I/D	0.78	1.30	4.8×10^{-4}
**9**	90719044	rs56257218	1>2	G/A	0.07	1.54	5.7×10^{-4}
**9**	98252899	chr9:98252899	1>2	D/I	0.86	1.38	5.0×10^{-4}
**9**	101490087	rs337570	2>1	C/T	0.62	1.25	4.8×10^{-4}
**9**	105695436	rs79931339	1>2	A/C	0.79	1.32	5.2×10^{-4}

**9**	113085232	rs17807186	2>1	G/A	0.35	1.28	1.5×10^{-4}
**9**	129468586	rs13299536	2>1	A/G	0.32	1.29	1.1×10^{-4}
**9**	135277259	rs186468471	1>2	T/C	0.99	2.78	3.1×10^{-4}
**9**	137514801	rs57187152	1>2	C/T	0.08	1.54	1.8×10^{-4}
**9**	138995207	rs59623632	1>2	A/G	0.28	1.31	1.4×10^{-4}
**9**	140143728	rs201094187	2>1	D/I	0.14	1.40	2.7×10^{-4}
**10**	8277297	rs192871141	2>1	A/C	0.91	1.44	5.0×10^{-4}
**10**	9034011	rs12776100	2>1	C/A	0.73	1.28	4.9×10^{-4}
**10**	13861399	rs72771044	1>2	A/T	0.55	1.29	1.0×10^{-4}
**10**	14134826	rs789767	1>2	T/G	0.68	1.30	1.3×10^{-4}
**10**	14818870	rs12256790	1>2	G/A	0.72	1.35	3.1×10^{-5}
**10**	14991430	rs7906967	1>2	C/A	0.32	1.33	5.1×10^{-5}
**10**	23217727	rs10828375	2>1	G/T	0.30	1.28	2.9×10^{-4}
**10**	23342159	rs4237359	2>1	G/A	0.60	1.28	1.0×10^{-4}
**10**	25528562	rs4749015	2>1	A/G	0.82	1.34	2.6×10^{-4}
**10**	32549517	rs76220129	1>2	C/G	0.07	1.59	2.8×10^{-4}
**10**	72276664	chr10:72276664	2>1	C/G	0.01	2.47	5.2×10^{-4}
**10**	72494629	rs4747092	1>2	G/A	0.45	1.31	2.4×10^{-5}
**10**	73334219	rs144584204	1>2	I/D	0.10	1.46	4.2×10^{-4}
**10**	73398722	rs61650587	1>2	G/A	0.78	1.32	2.3×10^{-4}
**10**	73483645	rs3802713	1>2	T/C	0.18	1.35	3.8×10^{-4}
**10**	78670661	rs1907746	1>2	G/A	0.81	1.34	2.4×10^{-4}
**10**	78773064	rs74948619	1>2	G/A	0.77	1.32	2.3×10^{-4}
**10**	87300091	rs1912299	2>1	T/C	0.74	1.28	4.7×10^{-4}
**10**	89474072	rs2302404	1>2	C/T	0.90	1.44	4.9×10^{-4}
**10**	89593380	rs151154388	1>2	T/C	0.87	1.44	1.2×10^{-4}
**10**	92756381	rs35945428	2>1	G/T	0.64	1.26	3.3×10^{-4}
**10**	95982217	rs4918159	2>1	A/G	0.48	1.24	4.1×10^{-4}
**10**	97484887	rs34826225	2>1	I/D	0.70	1.31	6.3×10^{-5}
**10**	97603178	rs3181129	2>1	T/G	0.72	1.28	2.8×10^{-4}
**10**	97734022	rs10509696	2>1	T/C	0.65	1.25	4.4×10^{-4}

**10**	101366135	rs5787355	1>2	D/I	0.39	1.28	1.7×10^{-4}
**10**	101433770	rs55714089	1>2	D/I	0.40	1.25	4.4×10^{-4}
**10**	101558746	rs2756109	1>2	G/T	0.55	1.27	2.2×10^{-4}
**10**	114272761	rs12780297	1>2	T/G	0.19	1.32	6.3×10^{-4}
**10**	121029044	rs61874482	2>1	A/G	0.10	1.46	2.8×10^{-4}
**10**	123804177	rs192412818	2>1	G/A	0.90	1.47	2.2×10^{-4}
**10**	131059102	rs12573583	2>1	C/G	0.92	1.61	2.4×10^{-5}
**11**	2189185	rs4074905	2>1	G/A	0.21	1.31	4.1×10^{-4}
**11**	2206387	rs11564707	1>2	G/C	0.43	1.39	2.4×10^{-7}
**11**	2286243	rs739543	1>2	A/G	0.44	1.26	3.3×10^{-4}
**11**	2857194	rs2237895	1>2	C/A	0.49	1.40	1.9×10^{-7}
**11**	2868868	rs79788804	2>1	A/G	0.36	1.30	6.2×10^{-5}
**11**	2931981	rs450208	1>2	T/G	0.69	1.33	3.3×10^{-5}
**11**	5463425	rs16931041	2>1	T/C	0.81	1.32	3.0×10^{-4}
**11**	11205233	rs2722772	2>1	A/G	0.50	1.28	6.5×10^{-5}
**11**	12309002	rs3736304	1>2	C/T	0.57	1.28	1.5×10^{-4}
**11**	20388097	rs7928503	1>2	G/A	0.73	1.29	3.8×10^{-4}
**11**	22781497	rs12422017	2>1	T/C	0.63	1.26	3.8×10^{-4}
**11**	27526304	rs114388342	2>1	T/C	0.04	1.72	5.2×10^{-4}
**11**	43255059	rs140092128	1>2	G/C	0.13	1.40	4.6×10^{-4}
**11**	61183329	rs17156014	1>2	C/T	0.25	1.30	4.9×10^{-4}
**11**	62528410	chr11:62528410	1>2	I/D	0.98	2.18	3.8×10^{-4}
**11**	63017480	rs118020373	1>2	C/A	0.73	1.31	2.4×10^{-4}
**11**	63215235	rs116986544	1>2	G/T	0.81	1.32	5.9×10^{-4}
**11**	68833565	rs189122876	2>1	C/T	0.14	1.39	2.6×10^{-4}
**11**	69448373	rs654240	2>1	T/C	0.40	1.27	1.6×10^{-4}
**11**	70518864	rs7104178	1>2	A/G	0.73	1.30	4.6×10^{-4}
**11**	70968452	rs117835161	1>2	A/G	0.22	1.46	1.3×10^{-6}
**11**	71019766	rs1660861	1>2	T/C	0.61	1.26	3.7×10^{-4}
**11**	77999865	chr11:77999865	2>1	G/A	0.05	1.78	7.2×10^{-5}
**11**	79415407	rs2105527	2>1	T/C	0.07	1.62	8.0×10^{-5}

**11**	81582466	rs149635064	2>1	C/T	0.07	1.53	5.9×10^{-4}
**11**	86628005	rs4944640	2>1	C/T	0.36	1.31	3.5×10^{-5}
**11**	110693356	rs184443	1>2	G/A	0.33	1.26	5.9×10^{-4}
**11**	112353922	chr11:112353922	1>2	A/T	0.96	1.75	2.9×10^{-4}
**11**	112476540	rs4291707	1>2	G/T	0.94	1.71	4.5×10^{-5}
**11**	113964043	rs77660877	2>1	C/T	0.85	1.35	5.4×10^{-4}
**11**	115658805	rs74369070	1>2	G/A	0.86	1.43	9.5×10^{-5}
**11**	119239689	rs680268	2>1	T/C	0.86	1.37	4.0×10^{-4}
**11**	126285301	rs7949566	1>2	G/A	0.53	1.28	1.1×10^{-4}
**11**	126601540	rs10893557	1>2	G/T	0.31	1.28	3.6×10^{-4}
**12**	3352183	rs77587	2>1	G/C	0.53	1.25	2.9×10^{-4}
**12**	4150894	rs78555230	2>1	C/T	0.06	1.65	8.5×10^{-5}
**12**	12823025	rs143606772	2>1	C/T	0.63	1.25	4.7×10^{-4}
**12**	17571193	rs183764686	1>2	G/A	0.05	1.71	3.6×10^{-4}
**12**	43990838	rs60167591	2>1	I/D	0.61	1.25	4.2×10^{-4}
**12**	44023884	rs1478618	2>1	A/G	0.58	1.24	5.2×10^{-4}
**12**	57856493	rs78179158	1>2	G/A	0.19	1.37	1.3×10^{-4}
**12**	58197231	rs60780489	1>2	T/G	0.24	1.30	5.2×10^{-4}
**12**	63450335	rs3913041	2>1	A/G	0.60	1.26	2.8×10^{-4}
**12**	81141539	rs1603216	1>2	G/A	0.86	1.38	4.7×10^{-4}
**12**	107743313	rs76454120	1>2	C/T	0.69	1.27	6.2×10^{-4}
**12**	117815549	rs526159	2>1	G/A	0.86	1.37	2.6×10^{-4}
**12**	128537287	rs7975535	1>2	T/C	0.34	1.28	2.3×10^{-4}
**12**	128709513	rs11059588	2>1	G/T	0.55	1.33	9.1×10^{-6}
**12**	129704082	rs78134285	2>1	T/G	0.06	1.56	5.7×10^{-4}
**13**	32569815	rs121046	2>1	G/A	0.46	1.24	5.5×10^{-4}
**13**	39279459	rs56255874	2>1	A/G	0.64	1.27	1.9×10^{-4}
**13**	40803985	rs71425795	1>2	C/T	0.32	1.28	2.8×10^{-4}
**13**	46342822	rs4942430	2>1	C/A	0.82	1.33	4.0×10^{-4}
**13**	54432416	rs1841058	1>2	C/T	0.79	1.32	4.0×10^{-4}
**13**	92982848	chr13:92982848	2>1	D/I	0.08	1.54	2.9×10^{-4}

**13**	93363027	chr13:93363027	2>1	G/C	0.08	1.60	6.9×10^{-5}
**13**	93586124	chr13:93586124	2>1	C/T	0.07	1.59	1.1×10^{-4}
**13**	93955178	chr13:93955178	2>1	T/C	0.08	1.58	1.0×10^{-4}
**13**	94070190	chr13:94070190	2>1	C/T	0.08	1.52	3.5×10^{-4}
**13**	94634966	chr13:94634966	2>1	A/G	0.08	1.55	1.5×10^{-4}
**13**	98833517	rs7986669	2>1	C/T	0.93	1.55	3.2×10^{-4}
**13**	100390236	rs17577153	2>1	A/G	0.88	1.47	5.4×10^{-5}
**13**	100456122	rs2390357	2>1	A/G	0.16	1.37	2.2×10^{-4}
**13**	101064282	rs12429310	1>2	G/A	0.85	1.38	2.8×10^{-4}
**13**	105339826	rs701565	1>2	A/C	0.44	1.25	5.2×10^{-4}
**13**	105898262	rs16966166	2>1	C/T	0.62	1.26	2.3×10^{-4}
**14**	33131189	rs12895513	1>2	A/G	0.58	1.24	5.9×10^{-4}
**14**	33152745	rs11156756	1>2	A/G	0.45	1.26	2.8×10^{-4}
**14**	41767938	rs1778367	1>2	T/G	0.11	1.43	3.4×10^{-4}
**14**	77396193	rs112854307	2>1	D/I	0.20	1.32	3.4×10^{-4}
**14**	78205694	rs185044034	1>2	C/T	0.91	1.47	5.2×10^{-4}
**14**	80185987	rs178388	2>1	A/C	0.25	1.34	5.6×10^{-5}
**14**	82431921	rs74963973	2>1	C/T	0.08	1.50	2.4×10^{-4}
**14**	103463561	rs7157813	1>2	A/G	0.87	1.44	2.0×10^{-4}
**15**	26153832	rs1877249	2>1	A/G	0.39	1.24	5.9×10^{-4}
**15**	26186978	rs71463472	2>1	A/C	0.10	1.44	4.6×10^{-4}
**15**	26273281	rs72625124	1>2	T/G	0.89	1.44	3.9×10^{-4}
**15**	32337237	rs4779563	2>1	T/C	0.63	1.29	7.7×10^{-5}
**15**	39163687	rs11632328	1>2	T/C	0.49	1.27	1.6×10^{-4}
**15**	39641049	rs74634178	2>1	C/T	0.96	1.87	8.3×10^{-5}
**15**	53879272	rs4447369	1>2	C/T	0.72	1.29	2.8×10^{-4}
**15**	54757794	rs57385376	2>1	A/C	0.88	1.39	4.9×10^{-4}
**15**	58132503	rs6493957	1>2	G/T	0.58	1.26	2.7×10^{-4}
**15**	58838440	rs1973024	1>2	T/C	0.37	1.26	4.0×10^{-4}
**15**	63595545	rs72212605	1>2	D/I	0.71	1.28	4.8×10^{-4}
**15**	74408580	rs117223627	1>2	C/T	0.90	1.48	1.6×10^{-4}

**15**	74903614	rs140284162	1>2	G/C	0.96	1.81	1.5×10^{-4}
**15**	79488593	rs7170146	1>2	A/G	0.56	1.30	4.9×10^{-5}
**15**	80818730	rs147006180	1>2	G/C	0.15	1.38	3.3×10^{-4}
**15**	82391732	rs11073033	1>2	A/G	0.14	1.39	3.6×10^{-4}
**15**	86103176	rs16941432	1>2	G/A	0.29	1.32	7.4×10^{-5}
**15**	87333293	rs16977987	1>2	G/A	0.74	1.28	5.7×10^{-4}
**15**	87422428	rs57116355	1>2	C/T	0.77	1.30	4.6×10^{-4}
**15**	88044080	rs34297582	2>1	A/G	0.76	1.30	2.9×10^{-4}
**15**	89180915	rs10152159	1>2	C/T	0.45	1.26	3.2×10^{-4}
**15**	91990598	rs34901612	2>1	G/A	0.16	1.34	5.9×10^{-4}
**15**	98701022	rs977832	2>1	T/C	0.89	1.48	9.2×10^{-5}
**16**	322345	rs74003728	2>1	C/T	0.95	1.71	1.9×10^{-4}
**16**	370443	rs117116749	1>2	A/G	0.02	2.45	3.0×10^{-4}
**16**	4060661	rs2239310	2>1	A/G	0.26	1.28	4.6×10^{-4}
**16**	6328022	rs2160166	2>1	T/C	0.17	1.36	2.1×10^{-4}
**16**	6916942	chr16:6916942	2>1	C/A	0.04	1.81	3.1×10^{-4}
**16**	7700265	rs184543795	1>2	G/A	0.12	1.43	3.7×10^{-4}
**16**	13355924	rs78178451	1>2	C/T	0.10	1.45	4.3×10^{-4}
**16**	17842398	rs12598358	2>1	G/A	0.89	1.44	2.4×10^{-4}
**16**	18863882	rs74337241	1>2	A/G	0.95	1.72	4.8×10^{-4}
**16**	24325877	chr16:24325877	2>1	T/A	0.96	1.71	5.2×10^{-4}
**16**	51106209	rs4785498	2>1	T/C	0.65	1.33	1.6×10^{-5}
**16**	52065724	rs1345322	2>1	G/A	0.57	1.25	3.8×10^{-4}
**16**	52169755	rs4785094	2>1	G/A	0.69	1.31	4.4×10^{-5}
**16**	52261784	rs13337467	2>1	G/T	0.65	1.33	1.2×10^{-5}
**16**	52424157	rs187680	2>1	A/C	0.46	1.30	2.4×10^{-5}
**16**	53606724	chr16:53606724	1>2	G/C	0.04	1.78	5.9×10^{-4}
**16**	53987525	chr16:53987525	1>2	T/A	0.04	2.00	2.4×10^{-5}
**16**	56223793	rs3859112	1>2	C/T	0.17	1.36	3.8×10^{-4}
**16**	56308431	rs12600108	1>2	A/C	0.27	1.31	1.3×10^{-4}
**16**	56944947	rs75109156	1>2	A/G	0.76	1.28	6.3×10^{-4}

**16**	61291839	rs16963175	1>2	A/G	0.86	1.44	6.1×10^{-5}
**16**	68867456	rs1801026	2>1	T/C	0.26	1.32	9.1×10^{-5}
**16**	73375719	rs4346228	2>1	G/A	0.54	1.24	5.5×10^{-4}
**16**	74062203	rs2716591	2>1	C/T	0.87	1.50	1.3×10^{-5}
**16**	79061586	chr16:79061586	2>1	T/C	0.95	1.67	5.8×10^{-4}
**16**	85822447	chr16:85822447	1>2	T/C	0.95	1.76	3.4×10^{-4}
**16**	86119528	rs9935120	2>1	T/C	0.17	1.33	4.2×10^{-4}
**16**	86120025	rs4843903	1>2	T/C	0.44	1.25	5.6×10^{-4}
**17**	2295633	rs2429919	2>1	C/A	0.70	1.27	4.7×10^{-4}
**17**	5846281	rs967787	2>1	A/G	0.41	1.24	6.6×10^{-4}
**17**	12562530	rs12603774	1>2	T/C	0.43	1.29	7.8×10^{-5}
**17**	48457722	rs77309724	2>1	G/C	0.90	1.42	6.7×10^{-4}
**17**	52943191	rs16955263	2>1	A/G	0.61	1.26	5.0×10^{-4}
**17**	55604097	rs74401513	2>1	T/C	0.95	1.65	5.8×10^{-4}
**17**	57556414	rs9890999	2>1	C/T	0.51	1.25	3.8×10^{-4}
**17**	58149745	rs3744375	2>1	A/G	0.67	1.34	1.1×10^{-5}
**17**	58212091	rs345171	2>1	T/C	0.77	1.31	3.5×10^{-4}
**17**	58749017	chr17:58749017	2>1	C/G	0.96	1.80	3.1×10^{-4}
**17**	58965766	chr17:58965766	2>1	C/T	0.96	1.81	3.0×10^{-4}
**17**	59214727	chr17:59214727	2>1	A/G	0.95	1.74	3.1×10^{-4}
**17**	63477337	rs187812044	1>2	G/A	0.15	1.37	3.4×10^{-4}
**17**	63722528	rs4346239	1>2	G/A	0.53	1.28	9.0×10^{-5}
**17**	71943095	rs145834533	2>1	C/T	0.36	1.25	4.9×10^{-4}
**17**	74336545	rs11077813	1>2	T/C	0.82	1.37	1.7×10^{-4}
**17**	75215103	rs7215278	1>2	G/T	0.64	1.27	2.6×10^{-4}
**17**	77831802	chr17:77831802	2>1	C/T	0.02	2.04	6.7×10^{-4}
**18**	5377643	rs34657168	1>2	T/G	0.85	1.36	4.3×10^{-4}
**18**	7127805	rs9965559	1>2	A/G	0.17	1.42	2.7×10^{-5}
**18**	7689006	rs4121621	2>1	G/A	0.24	1.31	1.9×10^{-4}
**18**	11694187	rs8090294	1>2	G/T	0.10	1.48	4.5×10^{-4}
**18**	26291497	rs16945199	2>1	G/A	0.87	1.43	1.1×10^{-4}

**18**	27562746	rs8097943	1>2	C/T	0.20	1.32	5.9×10^{-4}
**18**	32076237	rs8092794	1>2	A/G	0.73	1.31	1.5×10^{-4}
**18**	37496313	rs138230935	2>1	D/I	0.15	1.34	5.8×10^{-4}
**18**	43856724	rs77784260	2>1	G/A	0.77	1.40	4.9×10^{-6}
**18**	45034793	rs7506616	2>1	T/C	0.37	1.25	5.3×10^{-4}
**18**	45059898	rs12604167	2>1	A/G	0.76	1.28	6.0×10^{-4}
**18**	45546445	rs12955249	1>2	C/T	0.49	1.30	3.5×10^{-5}
**18**	60111809	rs11152345	1>2	C/A	0.15	1.42	9.6×10^{-5}
**18**	61540993	rs184075750	2>1	A/G	0.94	1.60	3.6×10^{-4}
**18**	64832417	rs7235347	1>2	C/T	0.52	1.25	5.5×10^{-4}
**18**	69308225	rs144134528	2>1	D/I	0.10	1.55	3.4×10^{-5}
**19**	1027797	rs7253254	1>2	C/A	0.15	1.38	3.4×10^{-4}
**19**	1138540	rs189976016	1>2	T/C	0.16	1.41	6.8×10^{-5}
**19**	10552211	chr19:10552211	2>1	T/C	0.96	1.82	4.0×10^{-4}
**19**	11411889	chr19:11411889	2>1	G/A	0.98	2.11	3.3×10^{-4}
**19**	11558451	rs111813645	2>1	A/G	0.95	1.69	1.2×10^{-4}
**19**	13876841	chr19:13876841	1>2	C/T	0.98	2.54	9.9×10^{-5}
**19**	13949434	chr19:13949434	1>2	G/A	0.98	2.56	8.4×10^{-5}
**19**	15529989	rs145008437	2>1	G/T	0.07	1.55	3.6×10^{-4}
**19**	29790947	rs79231989	2>1	T/C	0.65	1.27	2.7×10^{-4}
**19**	29998033	rs34950765	2>1	I/D	0.86	1.36	6.2×10^{-4}
**19**	30897578	rs79725917	2>1	C/T	0.82	1.34	3.6×10^{-4}
**19**	35757250	rs916147	1>2	A/G	0.30	1.28	2.7×10^{-4}
**19**	40094528	rs1014206	2>1	G/A	0.57	1.24	6.6×10^{-4}
**19**	41172997	chr19:41172997	2>1	G/C	0.98	2.32	3.1×10^{-4}
**19**	42985831	rs16975834	2>1	G/A	0.09	1.49	1.7×10^{-4}
**19**	44953196	rs73935677	2>1	A/G	0.90	1.43	5.9×10^{-4}
**19**	45202027	rs12150984	2>1	A/G	0.22	1.29	6.7×10^{-4}
**19**	45379516	rs412776	2>1	A/G	0.18	1.33	3.8×10^{-4}
**19**	46262673	chr19:46262673	1>2	A/G	0.01	5.22	4.3×10^{-4}
**19**	47774666	rs138370983	1>2	D/I	0.36	1.26	4.3×10^{-4}

**19**	49263886	rs78627671	1>2	A/G	0.10	1.52	5.6×10^{-5}
**19**	53482311	rs145685675	1>2	I/D	0.20	1.34	2.7×10^{-4}
**19**	54333563	rs71363373	1>2	A/C	0.20	1.32	4.6×10^{-4}
**20**	292327	rs6076049	2>1	C/T	0.34	1.25	4.5×10^{-4}
**20**	364747	rs7265169	2>1	C/A	0.54	1.24	6.4×10^{-4}
**20**	550815	rs2665782	2>1	A/C	0.57	1.26	2.3×10^{-4}
**20**	2375262	rs2076405	1>2	A/G	0.03	1.98	2.4×10^{-4}
**20**	3780962	chr20:3780962	2>1	D/I	0.01	3.94	1.6×10^{-4}
**20**	22732845	rs17827832	2>1	G/T	0.17	1.34	4.6×10^{-4}
**20**	44577244	rs189615511	2>1	C/T	0.96	1.81	1.1×10^{-4}
**20**	46410015	rs6012265	1>2	C/T	0.24	1.36	5.3×10^{-5}
**20**	52468966	rs4811476	2>1	T/C	0.42	1.25	3.7×10^{-4}
**20**	57155037	rs185637455	2>1	C/T	0.96	1.77	3.3×10^{-4}
**20**	62772637	rs2983435	1>2	C/T	0.54	1.25	5.3×10^{-4}
**21**	22436984	rs62207566	1>2	G/A	0.89	1.50	7.6×10^{-5}
**21**	25536983	rs186705241	1>2	C/T	0.07	1.52	5.7×10^{-4}
**21**	27397380	rs149868148	2>1	C/T	0.07	1.53	5.0×10^{-4}
**21**	33782887	rs2211789	2>1	T/C	0.54	1.27	1.1×10^{-4}
**21**	34423899	rs2834078	2>1	T/G	0.65	1.25	6.5×10^{-4}
**21**	36469037	rs984657	1>2	C/T	0.63	1.26	4.2×10^{-4}
**21**	39738517	rs34613855	2>1	A/G	0.85	1.36	3.3×10^{-4}
**21**	39788503	rs2836379	2>1	A/C	0.91	1.48	2.9×10^{-4}
**21**	41580503	rs117371768	2>1	G/A	0.06	1.61	3.2×10^{-4}
**21**	41808578	rs76272754	2>1	T/G	0.08	1.46	6.2×10^{-4}
**21**	41877887	rs7282270	2>1	A/G	0.18	1.31	6.1×10^{-4}
**21**	43142343	rs55711022	1>2	G/C	0.55	1.24	6.0×10^{-4}
**21**	43144340	rs7283002	2>1	C/T	0.75	1.34	5.8×10^{-5}
**21**	43530575	rs220124	2>1	T/C	0.32	1.31	5.7×10^{-5}
**22**	17428896	rs148856575	1>2	T/G	0.10	1.50	2.4×10^{-4}
**22**	26469229	rs9613096	2>1	A/G	0.77	1.30	4.6×10^{-4}
**22**	27424318	rs12483907	1>2	A/G	0.13	1.41	3.2×10^{-4}

**22**	36133739	rs5001172	2>1	A/G	0.70	1.30	8.0×10^{-5}
**22**	39939913	rs12628516	2>1	A/G	0.90	1.41	6.4×10^{-4}
**22**	43006541	rs182783110	2>1	T/C	0.08	1.54	2.0×10^{-4}
**22**	43867177	rs8142313	1>2	G/C	0.51	1.27	2.5×10^{-4}
**22**	46266351	rs117192130	1>2	A/G	0.05	1.69	1.8×10^{-4}
**22**	47372330	rs4439	1>2	T/C	0.38	1.27	4.4×10^{-4}
**X**	16675963	rs6632861	2>1	G/A	0.73	1.22	5.7×10^{-4}
**X**	19785191	rs148543908	2>1	G/A	0.05	1.48	5.0×10^{-4}
**X**	19942966	rs7060796	2>1	A/G	0.06	1.45	3.6×10^{-4}
**X**	30119777	rs6628506	1>2	G/A	0.54	1.21	2.3×10^{-4}
**X**	32934766	rs5928113	2>1	A/G	0.09	1.38	3.6×10^{-4}
**X**	35847989	rs1536848	2>1	G/A	0.25	1.23	3.3×10^{-4}
**X**	44226172	rs5953389	2>1	A/G	0.40	1.23	5.5×10^{-5}
**X**	84534059	rs6617041	1>2	A/C	0.18	1.29	1.7×10^{-4}
**X**	111659846	rs6643034	2>1	T/C	0.21	1.26	2.4×10^{-4}
**X**	125060265	rs5932947	2>1	C/T	0.66	1.20	4.6×10^{-4}
**X**	125583629	rs5931011	2>1	G/A	0.62	1.22	1.2×10^{-4}
**X**	135769607	rs141122592	2>1	T/C	0.93	1.43	2.8×10^{-4}
**X**	150396570	rs760105	2>1	A/G	0.24	1.23	5.4×10^{-4}

[a] Chr is chromosome.
[b] Position is in base pairs (bp) on Genome Reference Consortium Human Build 37.
[c] Subsets represent, which of the two randomly assigned groups is the discovery group (listed before the arrow) or the target group (listed after the arrow). The frequency, OR and p-value is reported for the discovery group.
[d] A1/a2 are the alleles with the effect allele given first.
[e] A1f is the frequency of the effect allele.
[f] OR is reported per copy of the effect allele.

Table S7. Results of survival, area under the ROC curve (AUC), and net reclassification improvement (NRI) analyses for the adult cohort with DIAGRAM 2018 T2D PS[a], with the inclusion of lipids traits.

	Models in Adult Cohort (age ≥ 20 years; N=1936)						Models in Youth Cohort (age 5-19 years, N-1802)					
	Clinical Variables[a] (AUC=0.744)			Clinical Variables + PS (AUC=0.751) (ΔAUC=0.007)			Clinical Variables (AUC=0.825)			Clinical Variables + PS (AUC=0.831) (ΔAUC=0.006)		
	**HR (95% CI)**	***p*-value**	**NRI**	**HR (95% CI)**	***p*-value**	**NRI**	**HR (95% CI)**	***p*-value**	**NRI**	**HR (95% CI)**	***p*-value**	**NRI**
Age (decades)	1.01 (0.91-1.11)	0.8786	0.092	1.03 (0.93-1.14)	0.5270	0.105	0.86 (0.51-1.45)	0.5709	-0.078	1.00 (0.59-1.70)	0.9873	0.116
Sex (F/M)	1.16 (0.95-1.42)	0.1456	0.098	1.16 (0.95-1.41)	0.1603	0.101	1.27 (0.89-1.81)	0.1907	-0.016	1.22 (0.86-1.75)	0.2653	-0.019
Mother Diabetic/NonDb	1.40 (1.08-1.81)	0.0610	0.125	1.34 (1.03-1.73)	0.1840	0.081	2.39 (1.67-3.43)	8.2×10^{-8}	0.593	2.16 (1.50-3.11)	1.4×10^{-5}	0.544
Mother Unknown/NonDb	1.27 (0.97-1.97)			1.22 (0.93-1.60)			1.51 (0.85-2.70)			1.48 (0.83-2.63)		
Father Diabetic/NonDb	1.29 (0.91-1.83)			1.21 (0.85-1.72)			1.92 (1.19-3.12)			1.72 (1.06-2.79)		
Father Unknown/NonDb	1.17 (0.86-1.58)			1.14 (0.84-1.54)			0.87 (0.59-1.29)			0.90 (0.60-1.33)		
BMI (kg/m^2 or ZSD)	1.02 (1.01-1.03)	0.0007	0.094	1.02 (1.01-1.04)	0.0001	0.143	1.44 (1.27-1.67)	3.6×10^{-8}	0.455	1.47 (1.29-1.67)	1.2×10^{-8}	0.495
Fasting Glucose (mmol/l)	2.14 (1.76-2.60)	2.6×10^{-14}	0.219	2.07 (1.70-2.53)	4.3×10^{-13}	0.170	3.24 (2.12-4.94)	5.0×10^{-8}	0.304	2.97 (1.95-4.55)	4.9×10^{-7}	0.205
HbA1c (mmol/mol)	1.09 (1.07-1.12)	3.5×10^{-16}	0.383	1.09 (1.07-1.11)	3.1×10^{-15}	0.368	1.06 (1.01-1.10)	0.0090	0.037	1.05 (1.01-1.09)	0.0189	0.006
Serum HDL cholesterol (mmol/l)	0.64 (0.45-0.92)	0.0158	0.179	0.65 (0.45-0.94)	0.0200	0.164	0.62 (0.27-1.41)	0.2510	0.147	0.59 (0.26-1.34)	0.2052	0.147
Serum Triglycerides (mmol/l)	1.00 (0.90-1.11)	0.9831	0.020	0.97 (0.87-1.08)	0.5529	0.095	1.24 (0.97-1.58)	0.0891	0.143	1.17 (0.91-1.51)	0.2131	0.033
Polygenic Score (SD)				1.25 (1.13-1.38)	9.1×10^{-6}	0.235				1.54 (1.28-1.86)	5.9×10^{-6}	0.311

[a] The weighted DIAGRAM 2018 PS is the sum of the number of T2D effect alleles multiplied by effect size across the 293 SNPs in the DIAGRAM 2018 PS (Table S4).

[b] HR is expressed per kg/m^2 in the adult cohort and per SD Z-score in the youth group. Results in the adult cohort are based on 450 events in 11,900.5 person-years, and results in the youth cohort are based on 139 events in 12,257.1 person-years

Table S8. Results of survival, area under the ROC curve (AUC), and net reclassification improvement (NRI) analyses for adult and youth cohorts with DIAGRAM 2018 T2D PS, without inclusion of HbA_{1c}.

A. Models in Adult Cohort (age ≥ 20 years; N=2783)

	Clinical Variables[a] (AUC=0.715)			Clinical Variables + PS (AUC=0.726)			Clinical Variables + 2hPG (AUC=0.742)			Clinical Variables + 2hPG + PS (AUC=0.750)		
	HR (95% CI)	*p*-value	NRI	HR (95% CI)	*p*-value	NRI	HR (95% CI)	*p*-value	NRI	HR (95% CI)	*p*-value	NRI
Age (decades)	1.04 (0.97, 1.12)	0.31	0.065	1.06 (0.98, 1.14)	0.13	0.065	1.01 (0.93, 1.08)	0.89	0.027	1.03 (0.95, 1.11)	0.51	0.056
Sex (F/M)	1.28 (1.11, 1.47)	6.53×10^{-4}	0.148	1.26 (1.09, 1.45)	1.47×10^{-3}	0.147	1.09 (0.94, 1.26)	0.24	0.147	1.08 (0.93, 1.24)	0.31	0.147
Mother Diabetic/NonDb	1.34 (1.11, 1.61)	4.25×10^{-4}	0.171	1.29 (1.07, 1.56)	2.24×10^{-3}	0.144	1.27 (1.05, 1.54)	2.88×10^{-3}	0.134	1.23 (1.01, 1.48)	9.77×10^{-3}	0.145
Mother Unknown/NonDb	1.34 (1.12, 1.61)			1.29 (1.07, 1.55)			1.27 (1.06, 1.53)			1.23 (1.02, 1.48)		
Father Diabetic/NonDb	1.45 (1.11, 1.90)			1.43 (1.10, 1.87)			1.43 (1.10, 1.87)			1.42 (1.09, 1.86)		
Father Unknown/NonDb	1.22 (0.98, 1.52)			1.21 (0.97, 1.50)			1.22 (0.98, 1.52)			1.21 (1.03, 1.50)		
BMI (kg/m²)	1.04 (1.03, 1.05)	1.96×10^{-19}	0.281	1.04 (1.03, 1.05)	6.90×10^{-23}	0.319	1.04 (1.03, 1.04)	2.79×10^{-15}	0.272	1.04 (1.03, 1.05)	3.65×10^{-18}	0.326
Fasting Glucose (mmol/l)	2.53 (2.21, 2.91)	1.21×10^{-40}	0.487	2.43 (2.12, 2.79)	2.03×10^{-37}	0.474	1.76 (1.52, 2.04)	2.71×10^{-14}	0.269	1.71 (1.48, 1.98)	3.86×10^{-13}	0.262
2-hour Glucose (mmol/l)							1.36 (1.30, 1.43)	1.10×10^{-35}	0.418	1.35 (1.28, 1.41)	1.69×10^{-33}	0.417
Polygenic Score (SD)				1.28 (1.19, 1.36)	1.78×10^{-12}	0.310				1.25 (1.16, 1.33)	1.25×10^{-10}	0.299

B. Models in Youth Cohort (age 5-19 years; N=2943)

	Clinical Variables (AUC=0.771)			Clinical Variables + PS (AUC=0.783)			Clinical Variables + 2hPG (AUC=0.787)			Clinical Variables + 2hPG + PS (AUC=0.796)		
	HR (95% CI)	*p*-value	NRI	HR (95% CI)	*p*-value	NRI	HR (95% CI)	*p*-value	NRI	HR (95% CI)	*p*-value	NRI
Age (decades)	1.29 (1.00, 1.05)	0.07	0.171	1.39 (1.06, 1.82)	0.02	0.202	1.24 (0.94, 1.63)	0.13	0.243	1.31 (0.99, 1.73)	0.06	0.254
Sex (F/M)	1.39 (1.15, 1.69)	8.62×10^{-4}	0.236	1.39 (1.14, 1.68)	1.10×10^{-3}	0.236	1.22 (1.00, 1.49)	0.05	0.218	1.24 (1.02, 1.51)	0.03	0.233
Mother Diabetic/NonDb	2.10 (1.679, 2.64)	1.33×10^{-12}	0.458	1.95 (1.55, 2.45)	8.32×10^{-10}	0.396	2.15 (1.71, 2.69)	3.92×10^{-13}	0.433	2.00 (1.59, 2.50)	2.12×10^{-10}	0.410
Mother Unknown/NonDb	1.67 (1.31, 2.11)			1.69 (1.33, 2.15)			1.67 (1.31, 2.11)			1.69 (1.33, 2.14)		
Father Diabetic/NonDb	1.73 (1.29, 2.33)			1.54 (1.14, 2.07)			1.75 (1.30, 2.35)			1.57 (1.17, 2.12)		
Father Unknown/NonDb	1.24 (1.00, 1.55)			1.20 (0.97, 1.50)			1.24 (1.00, 1.55)			1.20 (0.97, 1.50)		
Modified BMI Z-score	1.41 (1.33, 1.50)	2.93×10^{-30}	0.710	1.42 (1.34, 1.50)	8.84×10^{-32}	0.707	1.35 (1.26, 1.43)	5.48×10^{-21}	0.588	1.36 (1.28, 1.45)	3.24×10^{-23}	0.576
Fasting Glucose (mmol/l)	2.32 (1.79, 3.00)	1.77×10^{-10}	0.349	2.09 (1.61, 2.70)	2.02×10^{-8}	0.342	1.61 (1.23, 2.11)	5.68×10^{-4}	0.108	1.51 (1.15, 1.98)	2.69×10^{-3}	0.056
2-hour Glucose (nmol/l)							1.35 (1.25, 1.46)	1.45×10^{-14}	0.494	1.31 (1.21, 1.41)	1.64×10^{-11}	0.456
Polygenic Score (SD)				1.42 (1.29, 1.57)	7.06×10^{-12}	0.233				1.36 (1.23, 1.51)	2.90×10^{-9}	0.188

The weighted DIAGRAM 2018 PS is the sum of the number of T2D effect alleles multiplied by effect size across the 293 SNPs in the DIAGRAM 2018 PS (Table S4).

[b] HR is expressed per kg/m² in the adult cohort and per SD Z-score in the youth group. Results in the adult cohort are based on 903 events in 25,110 person-years, and results in the youth cohort are based on 447 events in 30,893 person-years

Table S9. Cox regressions comparing associations of the DIAGRAM 2018 PS (N=293 SNPs) with T2D incidence after adjustment for the first 10 genetic principal components[a]: birth, youth, and adult cohorts.

Cohort	HR[b] (95% HR CI) for the PS	AUC (and ΔAUC) for clinical model including the PS	NRI for the PS
Birth cohort (N=2894)[c]	1.43 (1.30, 1.57)	0.673 (0.060)	0.299
Youth cohort (N=2229)[d]	1.44 (1.24, 1.67)	0.811 (0.006)	0.246
Adult cohort (N=2333)[e]	1.24 (1.14, 1.35)	0.733 (0.005)	0.231

[a] Association assessed with residual T2D PS after adjusting for 1st 10 genetic PCs, estimated in each target cohort separately
[b] Only HRs and 95% HR confidence intervals (CIs) for PSs, not for clinical variables, are specified across PSs and cohorts in this table.
[c] Model in birth cohort was adjusted for clinical variables: sex, parental diabetes, and birth weight.
[c] Model in youth cohort was adjusted for clinical variables: age, sex, parental diabetes, modified BMI *z* score, fasting plasma glucose and Hb_{A1c}.
[d] Model in adult cohort was adjusted for clinical variables: age, sex, parental diabetes, BMI, fasting plasma glucose and Hb_{A1c}.

Table S10. Comparing analyses of PS constructions in birth, youth, and adult cohorts.

Table S10a. Cox regression, AUC, and NRI analyses results: birth cohort (N=2894).

PS Construction	HR (95% CI)[a,b]	ΔAUC[c] (95% CI)	NRI[d] (95% CI)
**AGEN 2020 (125 SNPs)**	1.27 (1.15, 1.39)	0.037 (0.013, 0.060)	0.164 (0.025, 0.303)
**DIAGRAM 2018 (293 SNPs)**	1.48 (1.35, 1.63)	0.072 (0.045 0.099)	0.362 (0.222, 0.502)
**DIAMANTE 2022 multi-ancestry composite (287 SNPs)**	1.45 (1.32, 1.60)	0.073 (0.046, 0.010)	0.259 (0.115, 0.403)
**DIAMANTE 2022 multi-ancestry African (276 SNPs)**	1.28 (1.16, 1.41)	0.042 (0.019, 0.065)	0.231 (0.091, 0.376)
**DIAMANTE 2022 multi-ancestry East Asian (280 SNPs)**	1.33 (1.22, 1.46)	0.052 (0.026, 0.077)	0.176 (0.029, 0.323)
**DIAMANTE 2022 multi-ancestry European (287 SNPs)**	1.47 (1.33, 1.62)	0.075 (0.048, 0.102)	0.328 (0.183, 0.474)
**DIAMANTE 2022 multi-ancestry Latino/Hispanic (287 SNPs)**	1.40 (1.28, 1.55)	0.072 (0.046, 0.099)	0.277 (0.125,0.428)
**DIAMANTE 2022 multi-ancestry South Asian (282 SNPs)**	1.32 (1.20, 1.45)	0.054 (0.031, 0.077)	0.201 (0.057, 0.306)
**DIAMANTE 2022 population-specific weight (287 SNPs)**	1.32 (1.20, 1.45)	0.057 (0.032, 0.081)	0.222 (0.068, 0.376)
**Population-specific variant (287 SNPs)**	1.41 (1.28, 1.54)	0.069 (0.044, 0.093)	0.227 (0.088, 0.366)

[a] Model in birth cohort was additionally adjusted for clinical variables: sex, parental diabetes, and birth weight.
[b] Only HRs and 95% HR confidence intervals (CIs) for PSs, not for clinical variables, are specified across PSs and cohorts in this table.
[c] ΔAUC is the difference in AUC between the model containing the PS and that containing clinical variables alone, which had AUC=0.614.
[d] NRI is calculated comparing the model with the PS to that containing clinical variables alone.

Table S10b. Cox regression, AUC, and NRI analyses results: youth cohort (N=2229).

PS Construction	HR (95% CI)[e,f]	ΔAUC[g] (95% CI)	NRI[h] (95% CI)
**AGEN 2020 (125 SNPs)**	1.28 (1.12, 1.46)	0.005 (-0.003, 0.012)	0.331 (0.141, 0.521)
**DIAGRAM 2018 (293 SNPs)**	1.49 (1.29, 1.72)	0.007 (-0.003, 0.017)	0.268 (0.072, 0.464)
**DIAMANTE 2022 multi-ancestry composite (287 SNPs)**	1.34 (1.17, 1.54)	0.004 (-0.001, 0.009)	0.314 (0.116, 0.512)
**DIAMANTE 2022 multi-ancestry African (276 SNPs)**	1.21 (1.06, 1.38)	0.005 (-0.001, 0.010)	0.185 (-0.017, 0.388)
**DIAMANTE 2022 multi-ancestry East Asian (280 SNPs)**	1.19 (1.04, 1.36)	0.001 (-0.004, 0.007)	0.182 (-0.019, 0.383)
**DIAMANTE 2022 multi-ancestry European (287 SNPs)**	1.37 (1.19, 1.58)	0.008 (-0.001, 0.017)	0.296 (0.090, 0.502)
**DIAMANTE 2022 multi-ancestry Latino/Hispanic (287 SNPs)**	1.32 (1.15, 1.51)	0.006 (-0.001, 0.014)	0.150 (-0.054, 0.354)
**DIAMANTE 2022 multi-ancestry South Asian (282 SNPs)**	1.35 (1.18, 1.56)	0.002 (-0.007, 0.012)	0.302 (0.089, 0.515)
**DIAMANTE 2022 population-specific weight (287 SNPs)**	1.24 (1.08, 1.41)	0.007 (0.000, 0.013)	0.345 (0.157, 0.533)
**Population-specific variant (287 SNPs)**	1.44 (1.26, 1.64)	0.017 (0.006, 0.028)	0.469 (0.274, 0.663)

[e] Only HRs and 95% HR confidence intervals (CIs) for PSs, not for clinical variables, are specified across PSs and cohorts in this table.
[f] Model in youth cohort was additionally adjusted for clinical variables: age, sex, parental diabetes, modified BMI *z* score, fasting plasma glucose and Hb_{A1c}. Only HRs and 95% HR confidence intervals (CIs) for PSs, not for clinical variables, are specified across PSs and cohorts in this table.
[g] ΔAUC is the difference in AUC between the model containing the PS and that containing clinical variables alone, which had AUC=0.805.
[h] NRI is calculated comparing the model with the PS to that containing clinical variables alone.

Table S10c. Cox regression, AUC, and NRI analyses results: adult cohort (N=2333).

PS Construction	HR (95% CI)[i,j]	ΔAUC[k] (95% CI)	NRI[l] (95% CI)
**AGEN 2020 (125 SNPs)**	1.14 (1.06, 1.24)	0.001 (-0.003,0.005)	0.115 (-0.014, 0.244)
**DIAGRAM 2018 (293 SNPs)**	1.27 (1.17, 1.38)	0.007 (0.001, 0.014)	0.270 (0.149, 0.392)
**DIAMANTE 2022 multi-ancestry composite (287 SNPs)**	1.20 (1.10, 1.30)	0.004 (-0.001, 0.009)	0.216 (0.094, 0.338)
**DIAMANTE 2022 multi-ancestry African (276 SNPs)**	1.13 (1.04, 1.22)	0.003 (0.000, 0.007)	0.181 (0.047, 0.316)
**DIAMANTE 2022 multi-ancestry East Asian (280 SNPs)**	1.13 (1.04, 1.23)	0.001 (-0.002, 0.005)	0.115 (-0.014, 0.244)
**DIAMANTE 2022 multi-ancestry European (287 SNPs)**	1.21 (1.12, 1.32)	0.005 (0.000, 0.011)	0.277 (0.156, 0.397)
**DIAMANTE 2022 multi-ancestry Latino/Hispanic (287 SNPs)**	1.18 (1.09, 1.29)	0.004 (-0.001, 0.008)	0.219 (0.097, 0.340)
**DIAMANTE 2022 multi-ancestry South Asian (282 SNPs)**	1.19 (1.09, 1.29)	0.004 (-0.001, 0.009)	0.181(0.054, 0.308)
**DIAMANTE 2022 population-specific weight (287 SNPs)**	1.14 (1.05, 1.24)	0.002 (-0.001, 0.006)	0.083 (-0.043, 0.208)
**Population-specific variant (287 SNPs)**	1.17 (1.09, 1.27)	0.002 (-0.003, 0.007)	0.108 (-0.011, 0.228)

[i] Only HRs and 95% HR confidence intervals (CIs) for PSs, not for clinical variables, are specified across PSs and cohorts in this table.
[j] Model in adult cohort was additionally adjusted for clinical variables: age, sex, parental diabetes, BMI, fasting plasma glucose and Hb_{A1c}. Only HRs and 95% HR confidence intervals (CIs) for PSs, not for clinical variables, are specified across PSs and cohorts in this table
[k] ΔAUC is the difference in AUC between the model containing the PS and that containing clinical variables alone, which had AUC=0.728.
[l] NRI is calculated comparing the model with the PS to that containing clinical variables alone.

Table S11. Cox regressions, AUC and NRI analyses of the multi-ancestry composite DIAMANTE 2022 PS: adult cohort (N=2333).

	Clinical Variables[a] (AUC=0.728)			Clinical Variables + PS (AUC=0.732)			Clinical Variables + 2hPG (AUC=0.760)			Clinical Variables + 2hPG + PS (AUC=0.764)		
	**HR (95% CI)**	***p*-value**	**NRI[b]**	**HR (95% CI)**	***p*-value**	**NRI**	**HR (95% CI)**	***p*-value**	**NRI**	**HR (95% CI)**	***p*-value**	**NRI**
**Age (decades)**	1.01 (0.93, 1.10)	0.869	0.011	1.04 (0.95, 1.13)	0.385	0.050	1.00 (0.92, 1.09)	0.992	-0.045	1.03 (0.94, 1.12)	0.531	0.098
**Sex (F/M)**	1.35 (1.14, 1.59)	5.48×10^{-4}	0.115	1.31 (1.10, 1.55)	1.83×10^{-3}	0.113	1.13 (0.953, 1.34)	0.157	0.110	1.10 (0.927, 1.31)	0.271	0.096
**Mother Diabetic/NonDb**	1.52 (1.21, 1.90)	2.75×10^{-4}	0.161	1.51 (1.21, 1.89)	3.12×10^{-4}	0.130	1.43 (1.14, 1.80)	2.60×10^{-3}	0.178	1.42 (1.14, 1.78)	2.88×10^{-3}	0.116
**Mother Unknown/NonDb**	1.45 (1.15, 1.82)			1.41 (1.12, 1.77)			1.39 (1.10, 1.74)			1.36 (1.08, 1.71)		
**Father Diabetic/NonDb**	1.45 (1.06, 1.96)			1.45 (1.07, 1.97)			1.38 (1.02, 1.88)			1.40 (1.03, 1.90)		
**Father Unknown/NonDb**	1.20 (0.921, 1.56)			1.17 (0.901, 1.52)			1.20 (0.925, 1.56)			1.18 (0.905, 1.53)		
**BMI (kg/m^2)**	1.02 (1.01, 1.03)	1.22×10^{-4}	0.158	1.02 (1.01, 1.03)	1.33×10^{-5}	0.201	1.02 (1.01, 1.03)	2.11×10^{-5}	0.242	1.03 (1.01, 1.04)	2.94×10^{-6}	0.257
**Fasting Glucose (mM)**	1.99 (1.70, 2.33)	2.78×10^{-17}	0.303	1.96 (1.90, 2.78)	3.27×10^{-16}	0.300	1.44 (1.22, 1.71)	2.51×10^{-5}	0.176	1.44 (1.21, 1.71)	2.90×10^{-5}	0.186
**HbA$_{1c}$ (mmol/mol)**	1.08 (1.06, 1.10)	1.18×10^{-18}	0.272	1.08 (1.06, 1.10)	1.13×10^{-17}	0.257	1.06 (1.05, 1.08)	9.64×10^{-13}	0.215	1.06 (1.05, 1.08)	3.72×10^{-12}	0.204
**2-hour Glucose (mM)**							1.32 (1.25, 1.39)	3.74×10^{-24}	0.420	1.31 (1.24, 1.39)	2.92×10^{-23}	0.427
**Polygenic Score (SD)**				1.20 (1.10, 1.30)	2.43×10^{-5}	0.216				1.17 (1.08, 1.28)	1.85×10^{-4}	0.223

Table S12. Cox regressions, AUC and NRI analyses of the population-specific variant PS: adult cohort (N=2333).

	Clinical Variables (AUC=0.728)			Clinical Variables + PS (AUC=0.730)			Clinical Variables + 2hPG (AUC=0.760)			Clinical Variables + 2hPG + PS (AUC=0.762)		
	**HR (95% CI)**	***p*-value**	**NRI**	**HR (95% CI)**	***p*-value**	**NRI**	**HR (95% CI)**	***p*-value**	**NRI**	**HR (95% CI)**	***p*-value**	**NRI**
**Age (decades)**	1.01 (0.93, 1.10)	0.869	0.011	1.03 (0.94, 1.12)	0.543	0.041	1.00 (0.92, 1.09)	0.992	-0.045	1.02 (0.93, 1.11)	0.702	0.084
**Sex (F/M)**	1.35 (1.14, 1.59)	5.48×10^{-4}	0.115	1.34 (1.13, 1.58)	7.51×10^{-4}	0.116	1.13 (0.953, 1.34)	0.157	0.110	1.14 (0.956, 1.35)	0.149	0.110
**Mother Diabetic/NonDb**	1.52 (1.21, 1.90)	2.75×10^{-4}	0.161	1.47 (1.18, 1.84)	3.48×10^{-3}	0.139	1.43 (1.14, 1.80)	2.60×10^{-3}	0.178	1.40 (1.12, 1.76)	0.0142	0.132
**Mother Unknown/NonDb**	1.45 (1.15, 1.82)			1.42 (1.13, 1.79)			1.39 (1.10, 1.74)			1.37 (1.09, 1.72)		
**Father Diabetic/NonDb**	1.45 (1.06, 1.96)			1.29 (0.947, 1.77)			1.38 (1.02, 1.88)			1.27 (0.930, 1.73)		
**Father Unknown/NonDb**	1.20 (0.921, 1.56)			1.12 (0.859, 1.46)			1.20 (0.925, 1.56)			1.14 (0.872, 1.48)		
**BMI (kg/m^2)**	1.02 (1.01, 1.03)	1.22×10^{-4}	0.158	1.02 (1.01, 1.03)	3.09×10^{-5}	0.178	1.02 (1.01, 1.03)	2.11×10^{-5}	0.242	1.02 (1.01, 1.03)	5.05×10^{-6}	0.289
**Fasting Glucose (mmol/l)**	1.99 (1.70, 2.33)	2.78×10^{-17}	0.303	1.97 (1.68, 2.31)	6.84×10^{-17}	0.291	1.44 (1.22, 1.71)	2.51×10^{-5}	0.176	1.45 (1.22, 1.71)	2.09×10^{-5}	0.191
**HbA$_{1c}$ (mmol/mol)**	1.08 (1.06, 1.10)	1.18×10^{-18}	0.272	1.08 (1.06, 1.10)	8.57×10^{-18}	0.271	1.06 (1.05, 1.08)	3.74×10^{-24}	0.215	1.06 (1.04, 1.08)	5.31×10^{-12}	0.226
**2-hour Glucose (mmol/l)**							1.32 (1.25, 1.39)	9.64×10^{-13}	0.420	1.31 (1.24, 1.39)	3.34×10^{-23}	0.419
**Polygenic Score (SD)**				1.17 (1.09, 1.27)	7.13×10^{-5}	0.108				1.15 (1.06, 1.25)	5.93×10^{-4}	0.117

[a] 'Clinical variables' for adult cohort refers to all the following variables: age, sex, parental diabetes, BMI, Hb$_{A1c}$ and FPG.
[b] NRI values were calculated for each variable by assessing its contribution to other variables (e.g., clinical variables and/or 2hPG), as specified by column headers

Table S13. AUC analyses for additional genotypes with DIAGRAM 2018 PS: adult cohort

Table S13a. AUC analyses for combinations of clinical variables, *ABCC8* rs1272388614 (R1420H) genotype and DIAGRAM 2018 PS, in adult cohort (N=2333).

Parameters in model	AUC
**age, sex, *ABCC8* genotype**	0.590
**age, sex, *ABCC8* genotype, PS**	0.620
**age, sex, *ABCC8* genotype, parental diabetes, FPG, HbA_{1c}**	0.728
**age, sex, ABCC8 genotype, parental diabetes, FPG, HbA_{1c}, PS**	0.735
**age, sex, *ABCC8* genotype, parental diabetes, FPG, 2hPG, HbA_{1c}**	0.760
**age, sex, *ABCC8* genotype, parental diabetes, FPG, 2hPG, HbA_{1c}, PS**	0.765

Table S13b. AUC analyses for combinations of clinical variables, *KCNQ1* rs2237895 genotype and DIAGRAM 2018 PS, in adult cohort (N=2333).

Parameters in model	AUC
**age, sex, maternally-derived *KCNQ1* risk allele, paternally-derived *KCNQ1* risk allele**	0.598
**age, sex, maternally-derived *KCNQ1* risk allele, paternally-derived *KCNQ1* risk allele, PS**	0.619
**age, sex, maternally-derived *KCNQ1* risk allele, paternally-derived *KCNQ1* risk allele, parental diabetes, FPG, HbA_{1c}**	0.723
**age, sex, maternally-derived *KCNQ1* risk allele, paternally-derived *KCNQ1* risk allele, parental diabetes, FPG, HbA_{1c}, PS**	0.727
**age, sex, maternally-derived *KCNQ1* risk allele, paternally-derived *KCNQ1* risk allele, parental diabetes, FPG, 2hPG, HbA_{1c}**	0.756
**age, sex, maternally-derived *KCNQ1* risk allele, paternally-derived *KCNQ1* risk allele, parental diabetes, FPG, 2hPG, HbA_{1c}, PS**	0.758

Table S13c. AUC analyses for combinations of clinical variables, *ABCC8* rs1272399614 (R1420H), *KCNQ1* rs2237895 genotypes and DIAGRAM 2018 PS, in adult cohort (N=2333).

Parameters in model	AUC
**age, sex, *ABCC8* genotype, maternally-derived *KCNQ1* risk allele, paternally-derived *KCNQ1* risk allele**	0.598
**age, sex, *ABCC8* genotype, maternally-derived *KCNQ1* risk allele, paternally-derived *KCNQ1* risk allele, PS**	0.619
**age, sex, *ABCC8* genotype, maternally-derived *KCNQ1* risk allele, paternally-derived *KCNQ1* risk allele, parental diabetes, FPG, HbA_{1c},**	0.730
**age, sex, *ABCC8* genotype, maternally-derived *KCNQ1* risk allele, paternally-derived *KCNQ1* risk allele, parental diabetes, FPG, HbA_{1c}, PS**	0.735
**age, sex, *ABCC8* genotype, maternally-derived *KCNQ1* risk allele, paternally-derived *KCNQ1* risk allele, parental diabetes, FPG, 2hPG, HbA_{1c}**	0.763
**age, sex, *ABCC8* genotype, maternally-derived *KCNQ1* risk allele, paternally-derived *KCNQ1* risk allele, parental diabetes, FPG, 2hPG, HbA_{1c}, PS[a]**	0.765

[a] Net reclassification improvement (NRI) values for each genetic variable in the bottommost row of Table S6c: 0.021 for *ABCC8* genotype, 0.148 for the maternally-derived and paternally-derived *KCNQ1* risk alleles, and 0.250 for the PS.

Supplementary appendix references

1. Piaggi P, Masindova I, Muller YL, et al. A genome-wide association study using a custom genotyping array identifies variants in GPR158 associated with reduced energy expenditure in American Indians. In: *Diabetes*. Vol 66. American Diabetes Association Inc.; 2017:2284-2295. doi:10.2337/db16-1565

2. Howie BN, Donnelly P, Marchini J. A Flexible and Accurate Genotype Imputation Method for the Next Generation of Genome-Wide Association Studies. Schork NJ, ed. *PLoS Genet*. 2009;5(6):e1000529. doi:10.1371/journal.pgen.1000529

3. Almasy L, Blangero J. Multipoint quantitative-trait linkage analysis in general pedigrees. *Am J Hum Genet*. 1998;62(5):1198-1211. doi:10.1086/301844

4. Browning BL, Browning SR. A fast, powerful method for detecting identity by descent. *Am J Hum Genet*. 2011;88(2):173-182. doi:10.1016/j.ajhg.2011.01.010

5. Haggstrom GW. Logistic Regression and Discriminant Analysis by Ordinary Least Squares. *J Bus Econ Stat*. 1983;1(3):229. doi:10.2307/1391344

6. Scheinost D, Noble S, Horien C, et al. Ten simple rules for predictive modeling of individual differences in neuroimaging. *Neuroimage*. 2019;193:35-45. doi:10.1016/j.neuroimage.2019.02.057

7. Spracklen CN, Horikoshi M, Kim YJ, et al. Identification of type 2 diabetes loci in 433,540 East Asian individuals. *Nature*. 2020;582(7811):240-245. doi:10.1038/s41586-020-2263-3

8. DIAbetes Genetics Replication And Meta-analysis (DIAGRAM) Consortium, Asian Genetic Epidemiology Network Type 2 Diabetes (AGEN-T2D) Consortium, South Asian Type 2 Diabetes (SAT2D) Consortium, et al. Genome-wide trans-ancestry meta-analysis provides insight into the genetic architecture of type 2 diabetes susceptibility. *Nat Genet*. 2014;46(3):234-244. doi:10.1038/ng.2897

9. Mahajan A, Spracklen CN, Zhang W, et al. Multi-ancestry genetic study of type 2 diabetes highlights the power of diverse populations for discovery and translation. *Nat Genet 2022 545*. 2022;54(5):560-572. doi:10.1038/s41588-022-01058-3
